# Dual Active Forms of Milstein’s Catalyst for Ester Hydrogenation

**DOI:** 10.1021/acs.organomet.6c00183

**Published:** 2026-08-05

**Authors:** Haitian Ye, Jason M. Keith, Anthony R. Chianese

**Affiliations:** Department of Chemistry, 3719 Colgate University, 13 Oak Drive, Hamilton, New York 13346, United States

## Abstract

In this article, we report that Milstein’s ruthenium-pincer catalyst for ester hydrogenation, previously shown to convert to a highly active form through release of ethane, is also moderately active in its original form prior to ethane release, provided an alcohol cocatalyst is present. We describe a detailed experimental and computational study of ester hydrogenation catalyzed by the catalyst’s original form. The reaction rate is first-order in ester and ruthenium catalyst, zero-order in hydrogen, and exhibits a saturation dependence on the concentration of the product alcohol. We establish that an equilibrium between two potential resting states, a dihydride complex and a hydridoalkoxide complex, is shifted nearly completely toward the dihydride under catalytically relevant conditions. We report a thorough computational analysis of the mechanism, which is consistent with the observed kinetics and catalyst resting speciation. In the minimum-energy pathway, ester reduction proceeds through dechelation of the pincer’s diethylamino group, and the CH_2_ linkers of the pincer ligand are not involved in catalytic turnover. Two alternative pathways involving H/OR metathesis or pincer ligand dearomatization proceed with free-energy barriers that are 1.1 and 1.5 kcal/mol higher, respectively; these computationally identified pathways are not fully consistent with the kinetic data.

## Graphical abstract

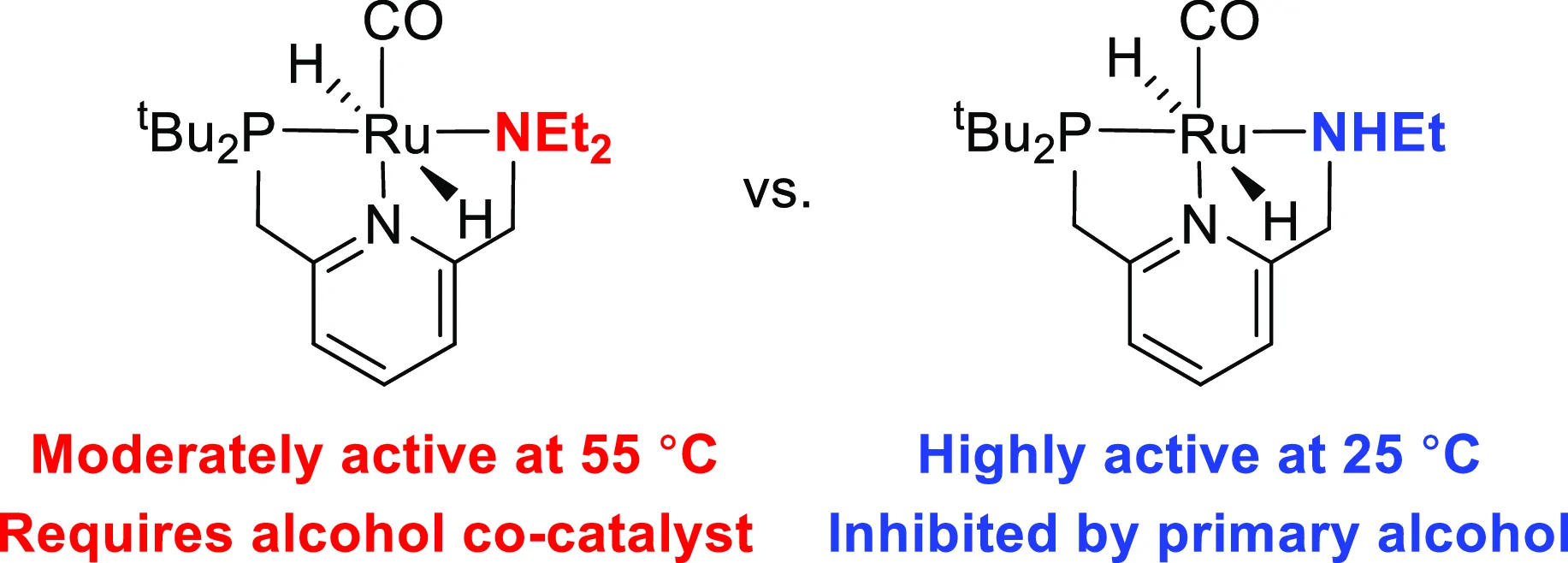

## Introduction

The field of metal–ligand-cooperative hydrogenation and dehydrogenation, initiated by the development of ruthenium catalysts for ketone hydrogenation by the groups of Noyori^1^ and Shvo,^2^ was advanced greatly by Milstein’s reports of the acceptorless dehydrogenative coupling of alcohols^3^ and its reverse, ester hydrogenation,^4^ catalyzed by the PNN-pincer complex **Ru-de** (Scheme 1). Milstein’s catalyst **Ru-de** was subsequently employed for a variety of related reactions, including the hydrogenation of carbon dioxide^5^ and carbonate esters,^6^ the dehydrogenative coupling of alcohols and amines to amides,^7^ and the hydrogenation of conjugated imines.^8^ **Ru-de** reacts reversibly reacts with hydrogen giving **Ru-H**, where the two hydrogen atoms are transferred to the ruthenium center and a carbon linker on the pincer ligand (Scheme 1). This novel bifunctional activation of hydrogen was proposed as a key step in the hydrogenation and dehydrogenation reactions catalyzed by **Ru-de**, and has become a new mechanistic paradigm in metal–ligand-cooperative catalysis.^9^

**Scheme 1:**
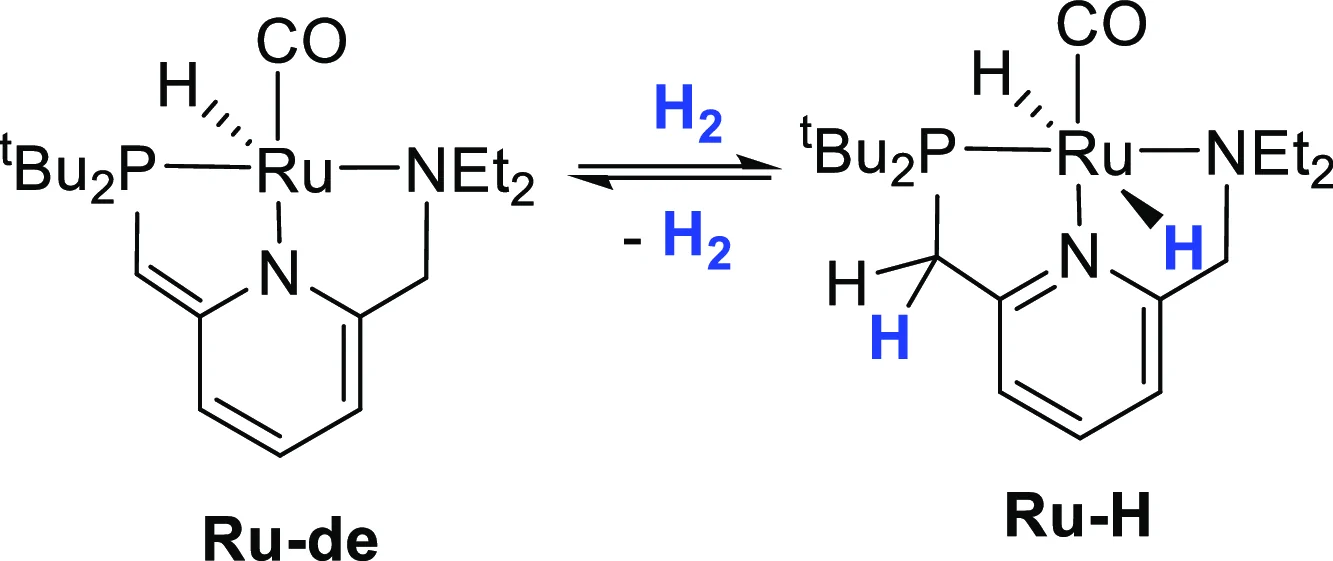
Reversible Addition of Dihydrogen to **Ru-de**

In their original work on ester hydrogenation, Milstein and co-workers proposed that hydrogen activation proceeds through the direct, concerted reaction of H_2_ with **Ru-de** to give **Ru-H**, and that ester reduction involves dechelation of the ligand NEt_2_ group followed by inner-sphere 1,2-insertion of C=O into Ru–H.^4^ Because of its broad catalytic utility, Milstein’s catalyst **Ru-de** has been the subject of many computational studies, including for the dehydrogenative formation of amides from alcohols and esters,^10^ the hydrogenation of carbonate esters,^11^ and the light-induced formation of O_2_ from H_2_O.^12^ We are aware of four computational studies that address the mechanism of ester hydrogenation catalyzed by **Ru-de**, which will be briefly summarized here.

In 2011, Wang and co-workers reported a DFT study of the dehydrogenative coupling of amines and alcohols to give amides.^10a^ To understand the selectivity for amide formation over ester formation from the coupling of two alcohol molecules, the authors studied the mechanism of ester formation, which is the microscopic reverse of ester hydrogenation. Considering this mechanism in the ester-hydrogenation direction, Wang and co-workers’ calculations indicated that hydrogen activation proceeds by the direct conversion of **Ru-de** to **Ru-H** through concerted heterolytic cleavage of H_2_ as predicted by Milstein. In Wang’s study, the minimum-energy pathway for ester reduction proceeds through a stepwise bifunctional double hydrogen transfer pathway, where **Ru-H** first transfers a hydride from Ru to the carbonyl carbon, and then transfers a proton from the PCH_2_ linker to the carbonyl oxygen, releasing a hemiacetal and regenerating **Ru-H**. The authors found that Milstein’s proposed inner-sphere insertion pathway involving dechelation of NEt_2_ had a higher overall barrier.

In 2013, Hasanayn and Baroudi described an alternative H/OR metathesis mechanism for ester reduction, where **Ru-H** mediates the concerted exchange of the alkoxy group on the ester with hydride, resulting in the direct release of an aldehyde and a ruthenium-alkoxide complex.^13^

In 2017, Zhang and co-workers described a DFT study of ester hydrogenation catalyzed by **Ru-de**.^14^ Their calculations were largely in agreement with the prior work of Wang and co-workers, in that hydrogen activation was found to proceed by the reaction of **Ru-de** with H_2_ to give **Ru-H**, and ester reduction in nonpolar solvents involved the stepwise bifunctional double hydrogen transfer mechanism. Zhang and co-workers found that the barrier for hydrogen activation was lower when a product alcohol molecule was included as a proton shuttle. Notably, Wang and co-workers had previously found that inclusion of a proton shuttle resulted in an increased barrier for hydrogenation activation.^10a^

In 2020, Gusev published a revised mechanism for ester hydrogenation and the reverse reaction catalyzed by **Ru-de**, based on a combined experimental and computational study.^15^ Experimentally, Gusev observed that **Ru-H** reacts rapidly and reversibly with ethanol to form **Ru-OEt** as shown in Scheme 2, where exchange between the ligand CH_2_ linkers, H_2_, the and alcohol OH and OCH_2_ hydrogens occurs rapidly on the NMR time scale. Notably, **Ru-de** was not observed in these reaction mixtures, indicating that reactions to form **Ru-H** and/or **Ru-OEt** from **Ru-de** are thermodynamically favored. Computationally, Gusev’s work provided important new insights. First, in contrast with the above reports from the groups of Wang^10a^ and Zhang^14^ favoring heterolytic activation of H_2_ by **Ru-de**, Gusev identified a low-barrier pathway where ruthenium-coordinated H_2_ is deprotonated by exogenous ethoxide, requiring an explicit molecule of ethanol but without involvement of the ligand CH_2_ linkers. A pathway proceeding through **Ru-de** was found to have a slightly higher barrier. For reduction of the ester by **Ru-H**, Gusev found that Hasanayn and Baroudi’s hydride/alkoxide metathesis pathway^13^ was preferred. Importantly, Gusev identified a complete, plausible catalytic cycle for ester hydrogenation without any necessary involvement of the ligand CH_2_ linkers or the intermediacy of **Ru-de**. In 2024, we showed that epoxide hydrogenolysis catalyzed by **Ru-de** similarly proceeds with no involvement of the CH_2_ linkers, with hydrogen activation occurring by the mechanism Gusev proposed.^16^

**Scheme 2:**
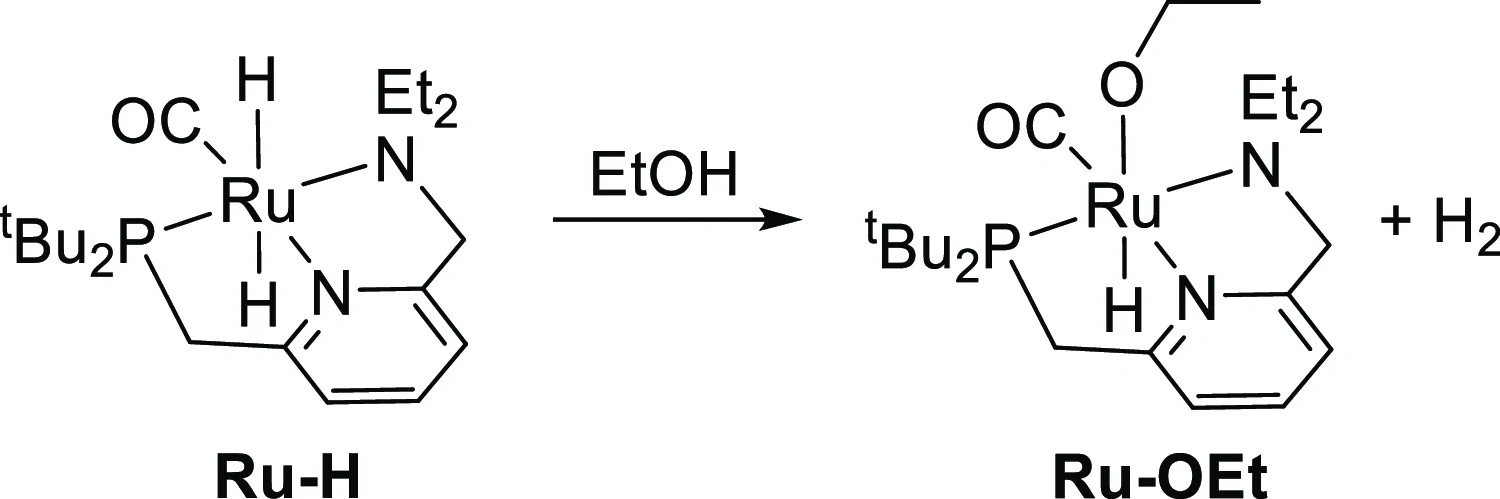
Displacement of Hydrogen by Ethanol in **Ru-H**, as Previously Observed by Gusev

In 2019, we demonstrated^17^ that **Ru-de** is inactive under the originally reported conditions for ester hydrogenation,^4^ and converts to a highly active form concomitant with release of ethane from the catalyst, which occurs over 1–2 h under catalytic conditions at 85 °C. When **Ru-de** is heated in the presence of PCy_3_, it releases an equivalent of ethane, giving the five-coordinate, 18-electron ruthenium(0) complex **Ru-imine** (Scheme 3). **Ru-imine** is an extremely active precatalyst for ester hydrogenation, giving more than 10,000 turnovers at room temperature for a range of ester substrates with no need for a basic additive.^17^ In this work, we showed that **Ru-imine** activates at room temperature by reaction with two equivalents of H_2_ to give the Noyori-type dihydride complex **Ru-NH** (Scheme 3, right). In a subsequent experimental/computational study, we determined the complete mechanism for ester hydrogenation catalyzed by **Ru-NH**, and showed that the nascent N–H group plays a key role in both hydrogen activation and carbonyl-group reduction.^18^ We also recently showed that the conversion of **Ru-imine** to **Ru-NH** is autocatalytic, with **Ru-NH** mediating the hydrogenation of the ligand C=N bond in **Ru-imine**.^19^

**Scheme 3:**
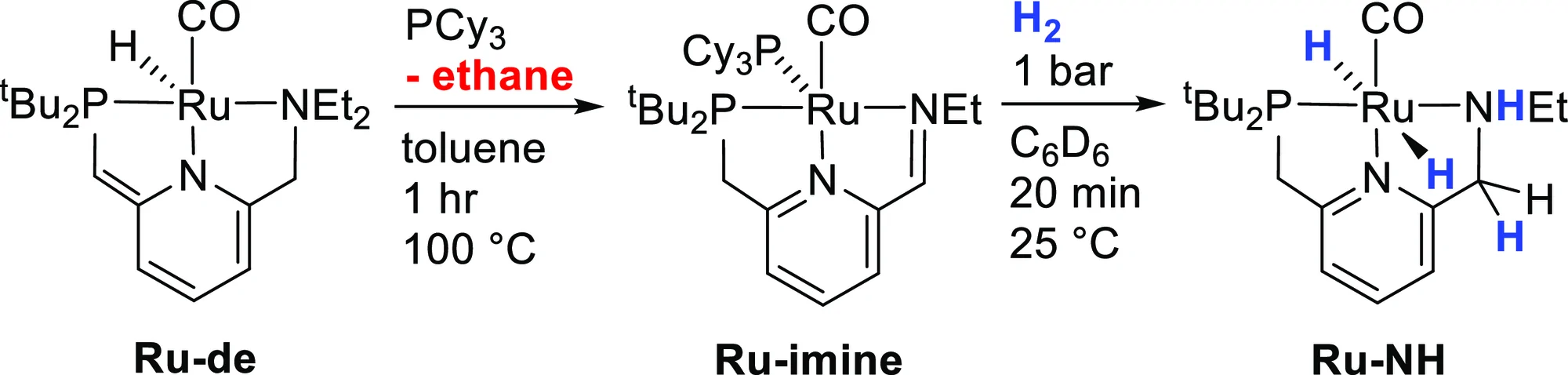
Conversion of **Ru-de** to **Ru-NH** by Ethane Release Followed by Reaction with H_2_

Because we demonstrated that Milstein’s catalyst **Ru-de** is inactive for ester hydrogenation prior to ethane release under the originally reported conditions, we hypothesized that ethane release might also be a necessary step in catalyst activation for related reactions catalyzed by **Ru-de**, and we set out to test this hypothesis. Although we observed the expected induction period for the hydrogenation of carbonate esters, we were surprised to find that the acceptorless dehydrogenation of primary alcohols to give esters–the microscopic reverse of ester hydrogenation–showed no induction period. This spurred us to reinvestigate the hydrogenation of esters catalyzed by **Ru-de** at 55 °C, a temperature low enough that ethane release to give the highly active form **Ru-NH** is not observed. We were surprised to discover that under these conditions, **Ru-de** is an active catalyst for ester hydrogenation, but only in the presence of an alcohol as cocatalyst. In this article, we describe a detailed experimental and computational study of the mechanism of ester hydrogenation catalyzed by **Ru-de**, with alcohol as a necessary cocatalyst.

## Results

### Carbonate Ester Hydrogenation

In our previous report,^17^ we observed an induction period in the hydrogenation of hexyl hexanoate catalyzed by **Ru-de**, concomitant with the release of ethane gas by the catalyst. In our initial experiments, we sought to determine whether a similar result is observed with related hydrogenation and dehydrogenation reactions catalyzed by **Ru-de**. In each case, we compared the activity of **Ru-de** with the activated form **Ru-imine**, which is known to convert to **Ru-NH** under a hydrogen atmosphere.^17^ We first turned to the hydrogenation of the carbonate ester dimethyl carbonate, which was previously described by Milstein and co-workers.^6^ In Milstein’s report, the hydrogenation was conducted at 145 °C in 1,4-dioxane, THF, or with no solvent, and turnover numbers up to 4400 were recorded. At this temperature, the dehydroalkylation reaction (Scheme 3) would likely be too fast for an induction period to be easily observed, so we conducted the reaction at 80 °C. We chose toluene as the solvent for consistency with our prior kinetic studies.^17,18^ We simultaneously monitored the reaction solution for consumption of dimethyl carbonate, and the reaction headspace for production of ethane gas. Figure 1 shows the result of this experiment.

**Figure 1:**
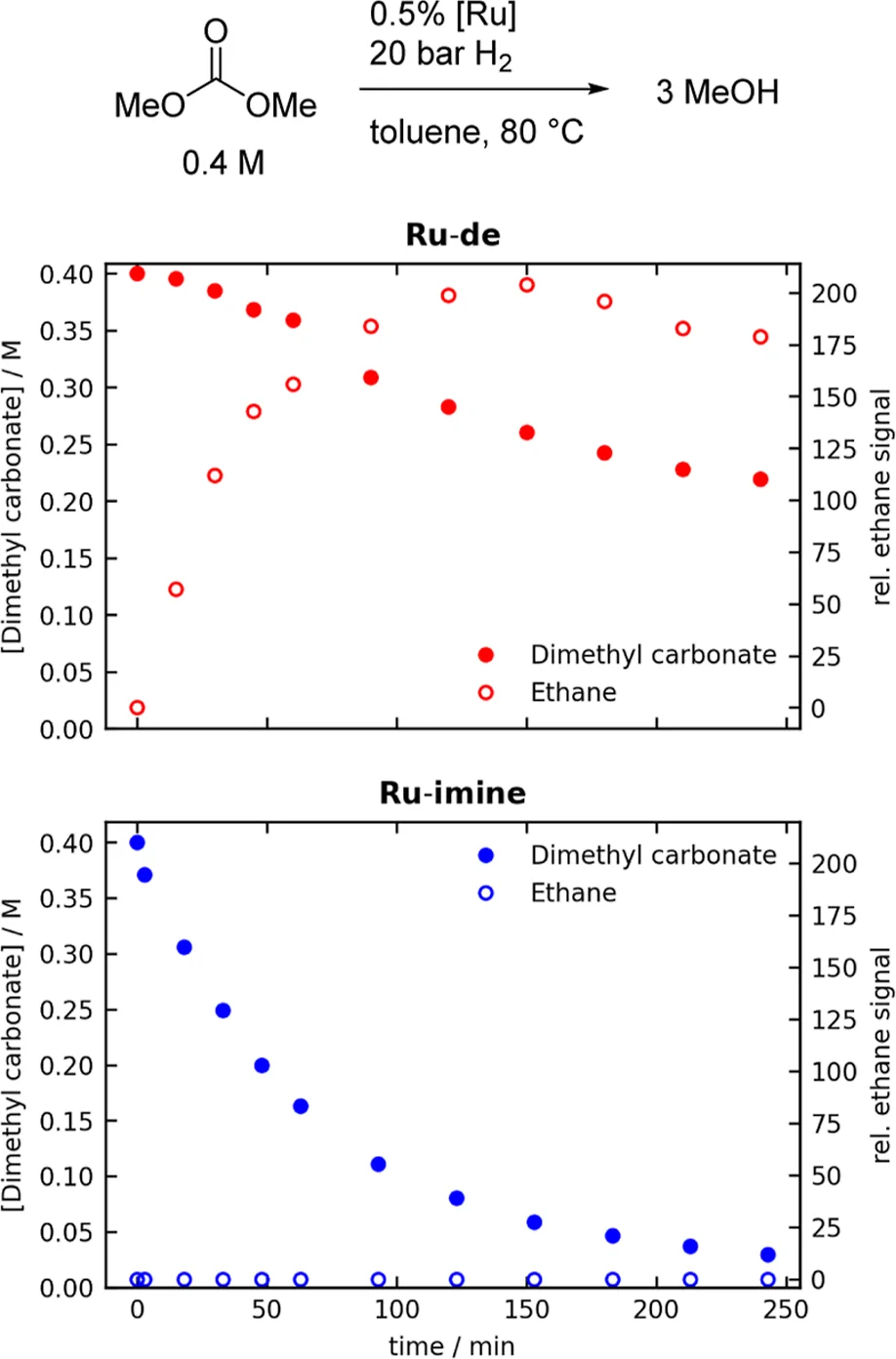
Hydrogenation of dimethyl carbonate catalyzed by **Ru-de** (top) and **Ru-imine** (bottom). Filled circles represent the concentration of dimethyl carbonate and open circles represent the relative ethane signal, measured by GC. No ethane was observed when **Ru-imine** was used as the catalyst.

In the hydrogenation of dimethyl carbonate catalyzed by **Ru-de** (top), the time course shows a clear induction period, concomitant with the release of ethane. This is consistent with **Ru-de** being inactive under these conditions, converting to an active form (likely **Ru-NH**) as ethane is released. In contrast, the time course shows no induction period when **Ru-imine**, known to rapidly react with hydrogen to form **Ru-NH**, is used as the catalyst. No ethane release was observed with **Ru-imine**. These results are consistent with our prior observations for ester hydrogenation catalyzed by **Ru-de** and **Ru-imine**.^17^

### Acceptorless Dehydrogenation of a Primary Alcohol

Next, we examined the dehydrogenation of 1-decanol to give the ester decyl decanoate. **Ru-de** is known to catalyze the dehydrogenation of primary alcohols to esters,^3^ and this reaction is expected to follow the microscopic reverse of the mechanism for ester hydrogenation. Acceptorless dehydrogenation reactions are typically conducted under reflux in an open system under inert gas flow, to continually purge the generated hydrogen gas and drive the equilibrium to the ester. To simultaneously monitor the conversion of the alcohol in solution and observe ethane released into the headspace, we conducted these reactions in a closed autoclave (25 mL reaction solution, 100 mL internal reactor volume) pressurized with argon (4 bar). The argon pressure facilitates removal of gas and liquid aliquots. Because the hydrogen gas product builds up in the reactor, chemical equilibrium is reached at approximately 50% conversion of the alcohol under these conditions. Figure 2 shows the results of this experiment.

**Figure 2:**
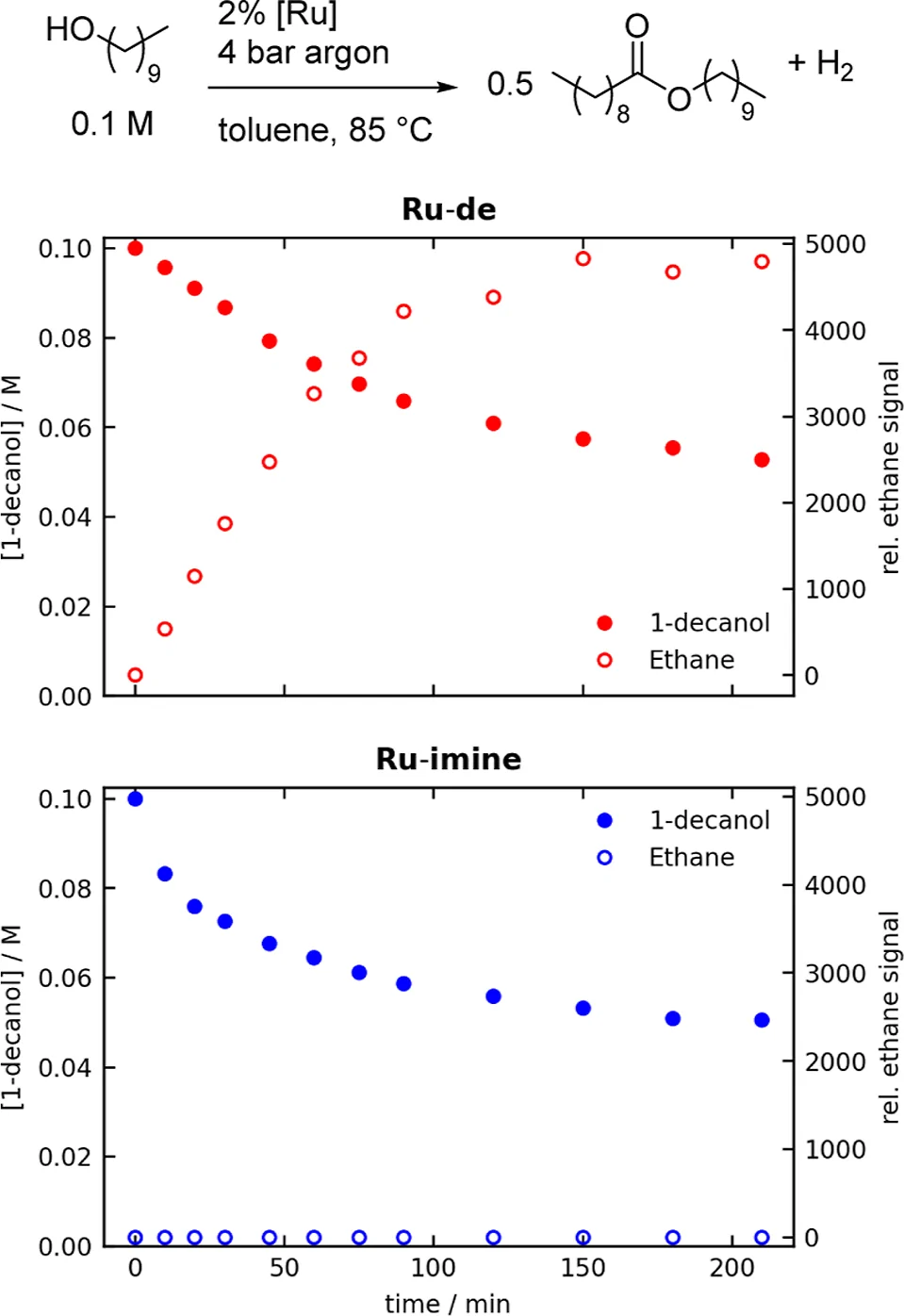
Dehydrogenation of 1-decanol catalyzed by **Ru-de** (top) and **Ru-imine** (bottom). Filled circles represent the concentration of 1-decanol and open circles represent the relative ethane signal, measured by GC. No ethane was observed when **Ru-imine** was used as the catalyst.

In keeping with the above results, **Ru-de** (top) releases ethane over the first 2 h of reaction, while **Ru-imine** (bottom) does not. However, no induction period is observed for either catalyst, indicating that both are active at the start of the reaction under these conditions. The observation that **Ru-de** does not show an induction period was particularly surprising, given that the same catalyst shows a clear induction period for the reverse reaction, ester hydrogenation.^17^

### Ester Hydrogenation

To understand why **Ru-de** shows an induction period for ester hydrogenation but not for the reverse reaction, we revisited our previous study^17^ of ester hydrogenation catalyzed by **Ru-de**. As in our previous study, we conducted the hydrogenation of hexyl hexanoate at 85 °C, under 20 bar of hydrogen, in toluene solvent. Figure 3 shows the results of these experiments.

**Figure 3:**
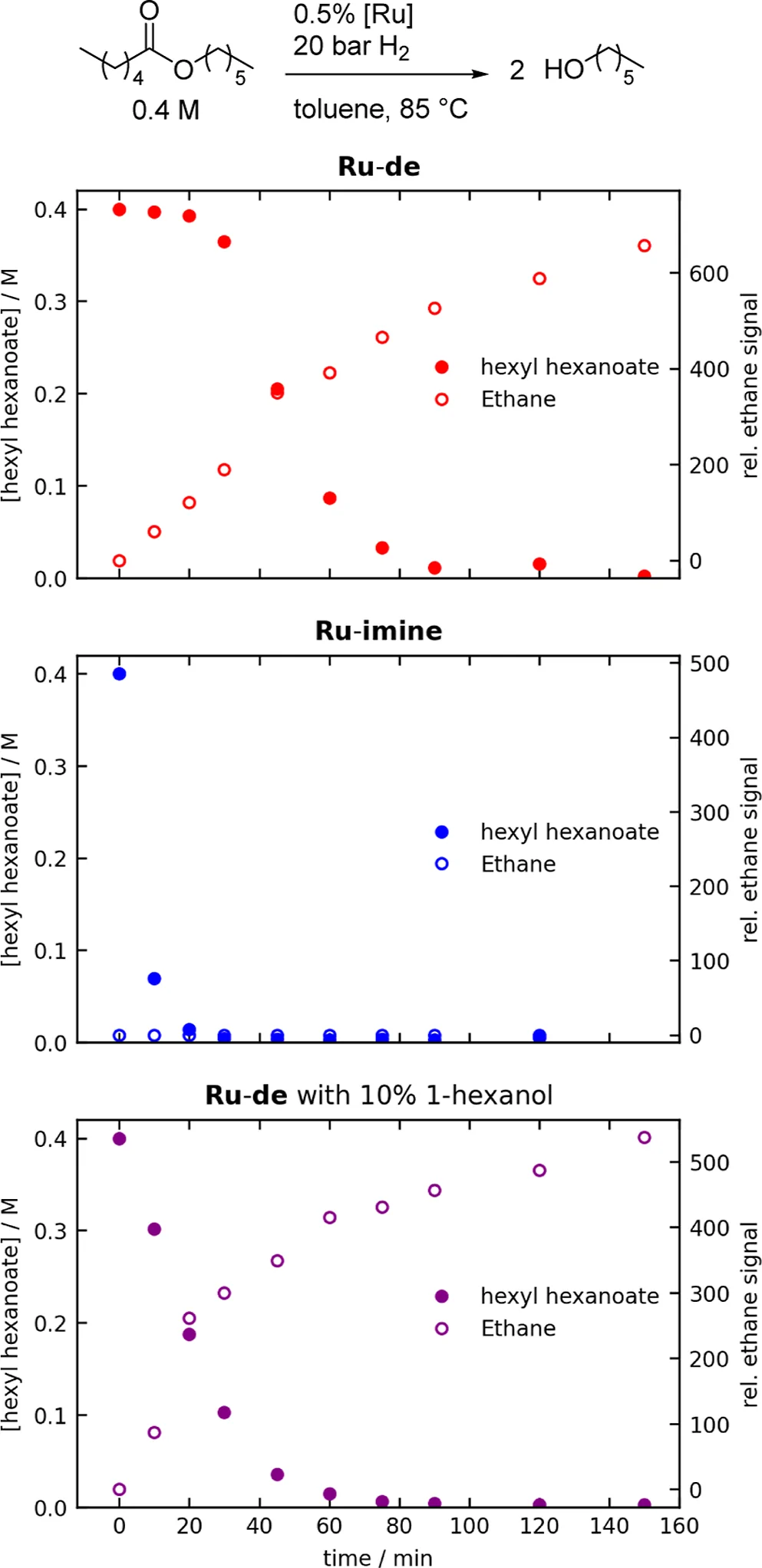
Hydrogenation of hexyl hexanoate catalyzed by **Ru-de** (top), **Ru-imine** (middle), and **Ru-de** with 10% added 1-hexanol (bottom). Filled circles represent the concentration of hexyl hexanoate and open circles represent the relative ethane signal, measured by GC. No ethane was observed when **Ru-imine** was used as the catalyst.

When **Ru-de** is used as the catalyst (top), the reaction proceeds with a clear induction period, and catalysis accelerates as ethane builds up in the headspace. With **Ru-imine** as catalyst (middle), the reaction is complete within 30 min, and no ethane release is observed. Both of these results are consistent with our prior study.^17^ We then reasoned that a key difference between the conditions for ester hydrogenation and alcohol dehydrogenation is the initial presence of an alcohol in the former reaction. Alcohols, as well as water, are well-known to aid in in the heterolytic activation of hydrogen^20^ by serving as a proton shuttle,^21^ and could conceivably serve as a cocatalyst in these reactions.

When we repeated the hydrogenation of hexyl hexanoate catalyzed by **Ru-de** with the addition of 10 mol % 1-hexanol (Figure 3, bottom), the reaction proceeded to completion within 1 h with no observable induction period. This result was truly surprising, as it indicated that **Ru-de** is an active catalyst both before and after the release of ethane, which results in conversion of the NEt_2_ group of the pincer ligand to an NHEt group under hydrogenation conditions. We previously completed a comprehensive experimental and computational study of the mechanism of ester hydrogenation catalyzed by **Ru-NH** (using **Ru-imine** as the precatalyst).^18^ **Ru-NH** catalyzes ester hydrogenation with an overall experimentally determined free-energy barrier of 17.4 kcal/mol at 25 °C, without the need for an alcohol cocatalyst, and the ligand N–H group is intimately involved in the mechanisms of hydrogen activation and carbonyl-group reduction. To understand the mechanism of ester hydrogenation catalyzed by **Ru-de**, which lacks the ligand N–H group, we conducted a mechanistic study under conditions where ethane release does not occur, as described below.

### Kinetic Study

Our first goal was to establish the empirical rate law for the hydrogenation of hexyl hexanoate catalyzed by **Ru-de**. We conducted the kinetic experiments described below at 55 °C, where ethane release–and the resulting catalysis by **Ru-NH**–is negligible. We monitored each ester hydrogenation reaction for 2.5 h, which resulted in conversion between 2% and 55%, sufficient for an accurate determination of the initial rates. We employed the standard conditions shown in Figure 4, and systematically varied the initial concentrations of **Ru-de**, hexyl hexanoate, and 1-hexanol, as well as the hydrogen pressure. Figure 4 shows the effect of each of these variables on the initial rate. The time course data for these experiments is included in Table S4.

**Figure 4:**
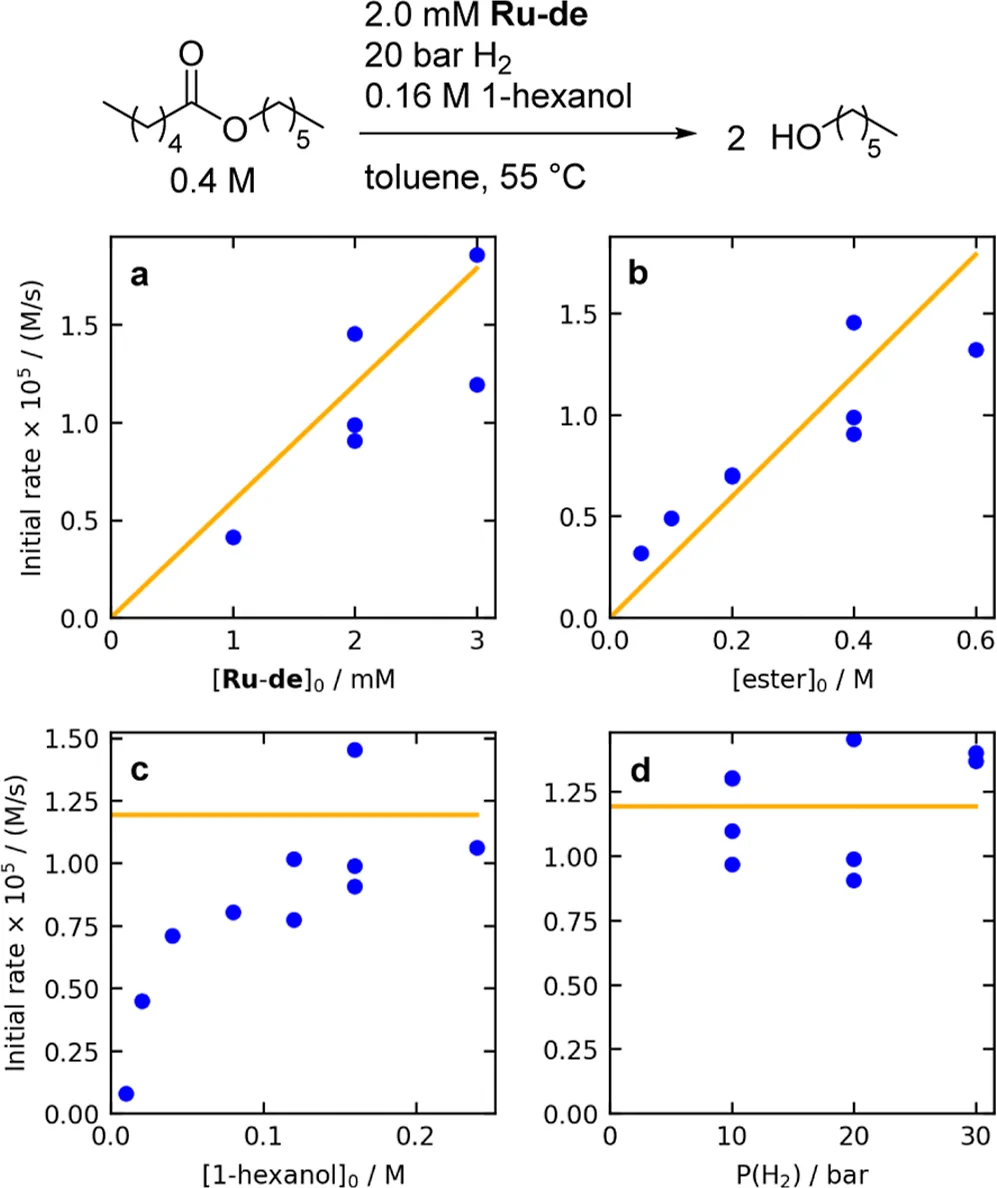
Initial rates for the hydrogenation of hexyl hexanoate catalyzed by **Ru-de**. (a) Variation of [**Ru-de**]_0_ from 1 to 3 mM; (b) variation of [ester]_0_ from 0.05 to 0.6 M; (c) variation of [1-hexanol]_0_ from 0 to 0.24 M; (d) variation of the hydrogen pressure from 10 to 30 bar. The blue points represent individual experiments and the orange lines represent the global kinetic model under conditions where the effect of [1-hexanol] on the rate is saturated.

As Figure 4 shows, the initial rate shows a first-order dependence on [**Ru-de**]_0_ and [ester]_0_. The initial rate does not vary as the hydrogen pressure is changed, indicating a zero-order dependence. No reaction is observed when 1-hexanol is not added, but as the initial 1-hexanol concentration is increased, the initial rate rapidly increases to a point of saturation around 0.1 M 1-hexanol. In accordance with the energetic span model,^22^ these data are consistent with a catalytic cycle involving one molecule of **Ru-de**, where the ester is incorporated along the sequence between the turnover frequency-determining intermediate (TDI) and the turnover frequency-determining transition state (TDTS). The lack of dependence of the rate on the hydrogen pressure is consistent with hydrogen being incorporated outside of the sequence from the TDI to the TDTS. The saturation dependence on the initial 1-hexanol concentration is consistent with the necessary involvement of 1-hexanol outside of the sequence from the TDI to the TDTS. The kinetic data presented in Figure 4 clearly contain a high level of run-to-run variability, which we believe results from the significant thermal instability of the precatalyst **Ru-de**. Despite this variability, we believe the key kinetic conclusions described above are sound based on the available data.

For comparison with the reaction kinetics predicted through computation (see below), we have determined the experimental rate law under conditions where the effect of [1-hexanol]_0_ on the rate is saturated. Using the 21 experiments where [1-hexanol]_0_ is at least 0.080 M, we find the following empirical rate law

$$rate=k\left[Ru‐de\right]\left[ester\right],k=0.015\pm 0.005\;M^{-1}\cdot s^{-1}$$ 1

Using the Eyring equation, we find that this rate constant corresponds to an activation free energy at 55 °C of 22.0 ± 0.2 kcal/mol. This overall activation barrier represents the experimental determination of the energetic span, or the free energy difference between the TDI and TDTS.^22^

### NMR Study of Catalyst Speciation

According to the energetic span model, the TDI is expected to be the observable catalyst resting state.^22^ **Ru-de** is known to react reversibly with hydrogen to form **Ru-H** (Scheme 1),^4,15^ and **Ru–H** reacts with alcohols to form hydridoalkoxide complexes with release of H_2_.^15,16^ To establish the resting catalyst speciation under our reaction conditions, we examined the reaction of **Ru-de** in toluene-d_8_ in the presence of varying concentrations of 1-hexanol (0.44 to 1.39 M) and varying pressures of hydrogen gas (1.0 to 1.7 bar). At 55 °C, a rapid equilibrium is established between **Ru-H** and **Ru-alkoxide**, as shown in Figure 5. Importantly, **Ru-de** is not observed under these conditions. A plot of [**Ru-H**]/[**Ru-alkoxide**] vs [hydrogen]/[1-hexanol] is well-modeled by a line with an intercept of zero, the slope of which gives the equilibrium constant of 25.8 ± 0.8 for this reaction. This equilibrium constant corresponds to a free-energy change Δ*G*° of −2.12 ± 0.02 kcal/mol at 55 °C.^23^

**Figure 5:**
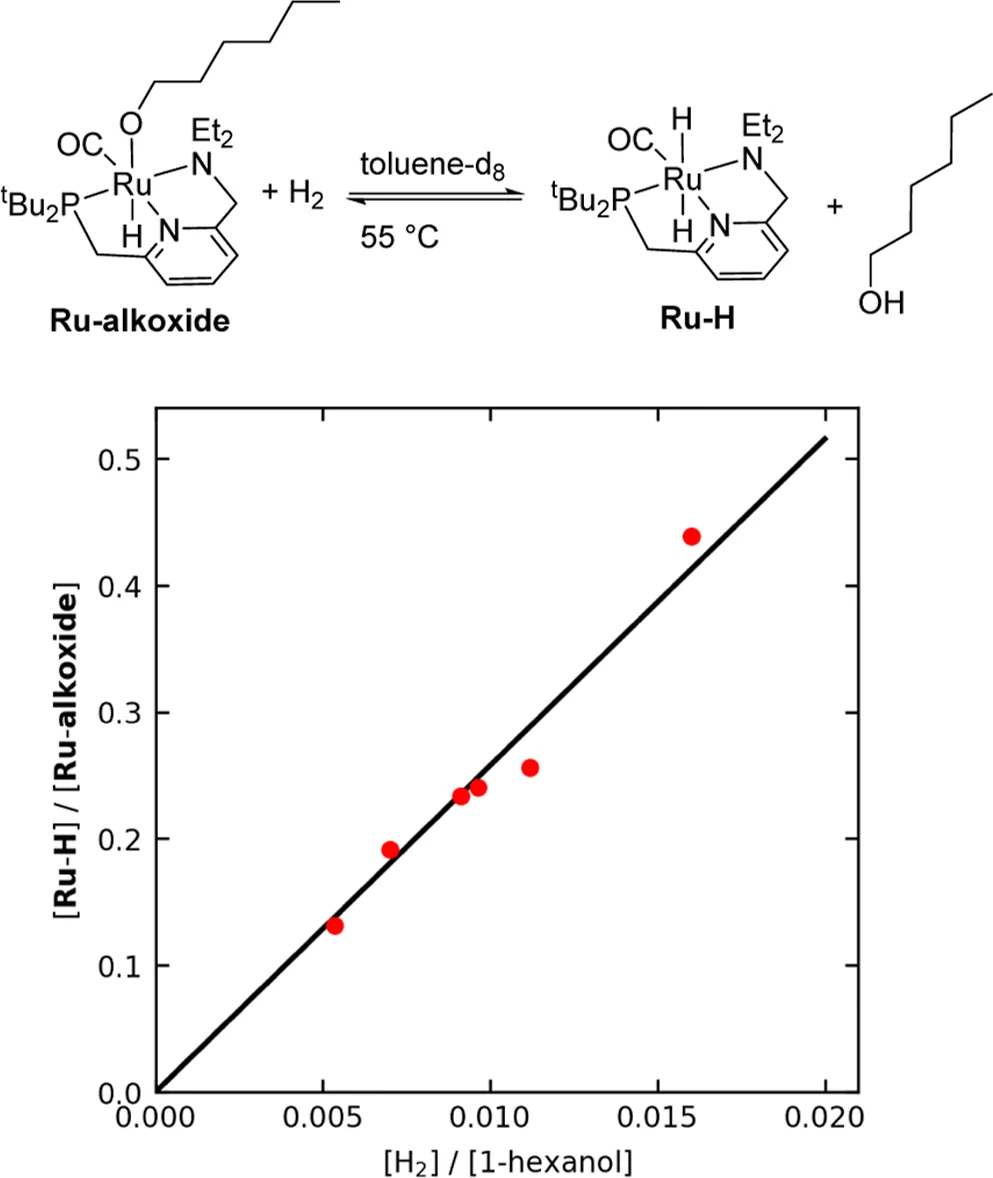
Determination of the equilibrium constant for the reaction of **Ru-alkoxide** with H_2_ to give **Ru-H** and 1-hexanol, in toluene-d_8_ at 55 °C. Red points represent individual experiments, and the black line shows the best fit with an intercept of zero.

At the low hydrogen pressures of 1.0 to 1.7 bar used in the NMR experiments, **Ru-H** represents 12% to 30% of the total ruthenium in solution. Under the conditions of the above kinetic studies where the hydrogen pressure varies from 10 to 30 bar, **Ru-H** is expected to comprise 89% to 96% of the total ruthenium species, indicating that it is the predominant resting state (TDI) in our kinetic experiments.

Although we have not observed additional ruthenium species in our experiments, we cannot exclude the possibility that additional, unidentified complexes form under the catalytic conditions. For example, Khaskin, Gusev, and co-workers characterized a range of ruthenium species formed under hydrogenation conditions for a closely analogous PNN-pincer-ruthenium complex with a bipyridine backbone, including products of pyridine ring hydrogenation and hydride-bridged dimers.^24^ In a subsequent detailed study, the authors showed that catalysis likely occurs through the action of species with a hydrogenated pyridine ring, which is capable of a Noyori-type mechanism involving a nascent N–H group.^25^ While we have not observed species of this nature, we cannot exclude the possibility that they form at low concentrations.

### Computational Analysis

As described in the Introduction, Milstein’s catalyst **Ru-de** has been the subject of many DFT studies,^10–16^ including four^10a,13–15^ that directly address ester hydrogenation. Our goal in this work was to identify a minimum-energy pathway (MEP) consistent with the above experimental data. Our NMR study of catalyst speciation indicates that the catalyst resting state (the TDI) is **Ru-H**. Our kinetic study indicates that an ester molecule is incorporated along the sequence from the TDI to the TDTS, with an overall energetic span of approximately 22.0 kcal/mol at 55 °C. Because the rate does not depend on the hydrogen pressure, incorporation of hydrogen should occur outside of this sequence. The MEP should also require the presence of exogenous alcohol, without its net incorporation in the sequence from the TDI to the TDTS. To exhaustively consider the prior computational studies^10a,13–15^ on equal footing, we began by recalculating all intermediates and transition states reported in those articles using the same high-level, well-benchmarked^26^ method (ωB97M-V^27^/def2-QZVPPD^28^//r^2^SCAN-3c^29^), where toluene solvent was modeled using the COSMO-RS^30^ method and free energies were calculated at 55 °C using a 1.0 M standard state for all species, including molecular hydrogen. To ensure that the lowest-energy conformers of intermediates and transition states were identified, we conducted conformational searches using CREST.^31^ Although our experimental studies focused on the hydrogenation of hexyl hexanoate, we have studied the simplified substrate ethyl acetate in computations, to minimize complications from conformations of the alkyl chains. The catalyst structure was modeled fully in all calculations. Because, as described below, the competing mechanisms for ester hydrogenolysis have similar barriers, we carefully considered multiple diastereomeric pathways for this sequence.

Broadly, ester hydrogenation proceeds by the bicyclic mechanism shown in Scheme 4. As described below, we have considered the incorporation of explicit ethanol molecules in potentially rate-determining intermediates and transition states. We find that explicit solvent has a pivotal role in the non-rate-determining activation of hydrogen and the hydrogenation of the intermediate aldehyde, but does not lower the free-energy barrier for the rate-determining ester hydrogenolysis sequence, in agreement with the kinetic data described above.

**Scheme 4:**
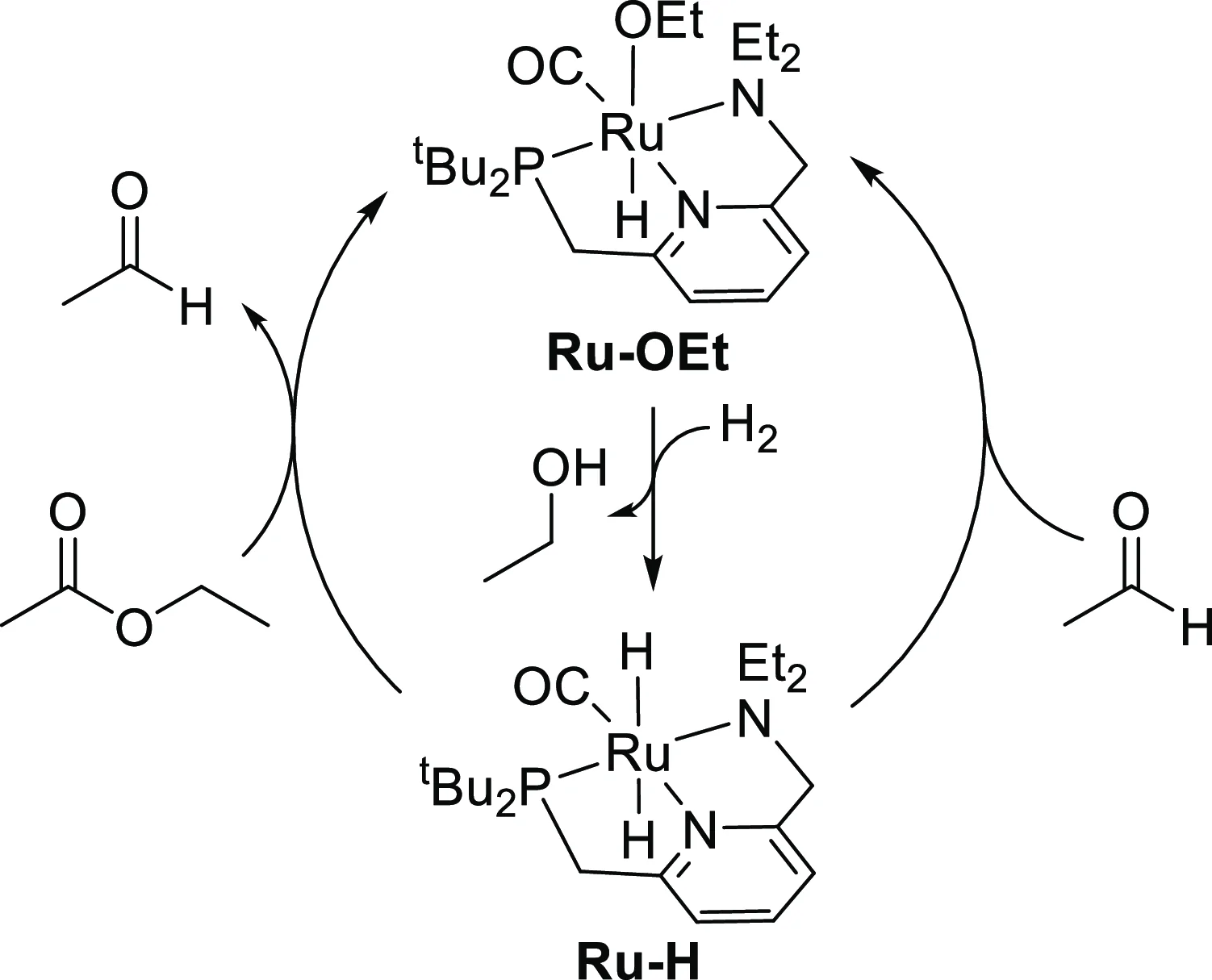
Overall Bicyclic Mechanism for Ester hydrogenation

### Examination of Potential Resting States

First, we compared the thermodynamic stability of plausible catalyst resting states. We considered the known complexes **Ru-H** and **Ru-OEt**, as well as the precatalyst **Ru-de**. In each case we examined the effect of adding one and two explicit ethanol molecules on the free energy. Figure 6 shows the results of these calculations. In a convention that we use throughout this article, a suffix of “-e” indicates one explicit ethanol molecule, and “-2e” indicates two explicit ethanol molecules. In agreement with the experimental result described above, we find that the most stable species overall is **Ru-H** with no explicit ethanol. **Ru-OEt-e** with one explicit ethanol is the next most stable species, with a free energy 2.0 kcal/mol higher than **Ru-H**. This compares well with the above experimental study, which showed that **Ru-alkoxide** was 2.1 kcal/mol higher in free energy than **Ru-H**. Consistent with our experimental observations and Gusev’s prior work,^15^ the dearomatized complex **Ru-de** is significantly less stable, 8.0 kcal/mol higher than **Ru-H**. In all computational results presented below, free energies are reported relative to the resting state **Ru-H**, along with the organic reactants ethyl acetate and hydrogen.

**Figure 6:**
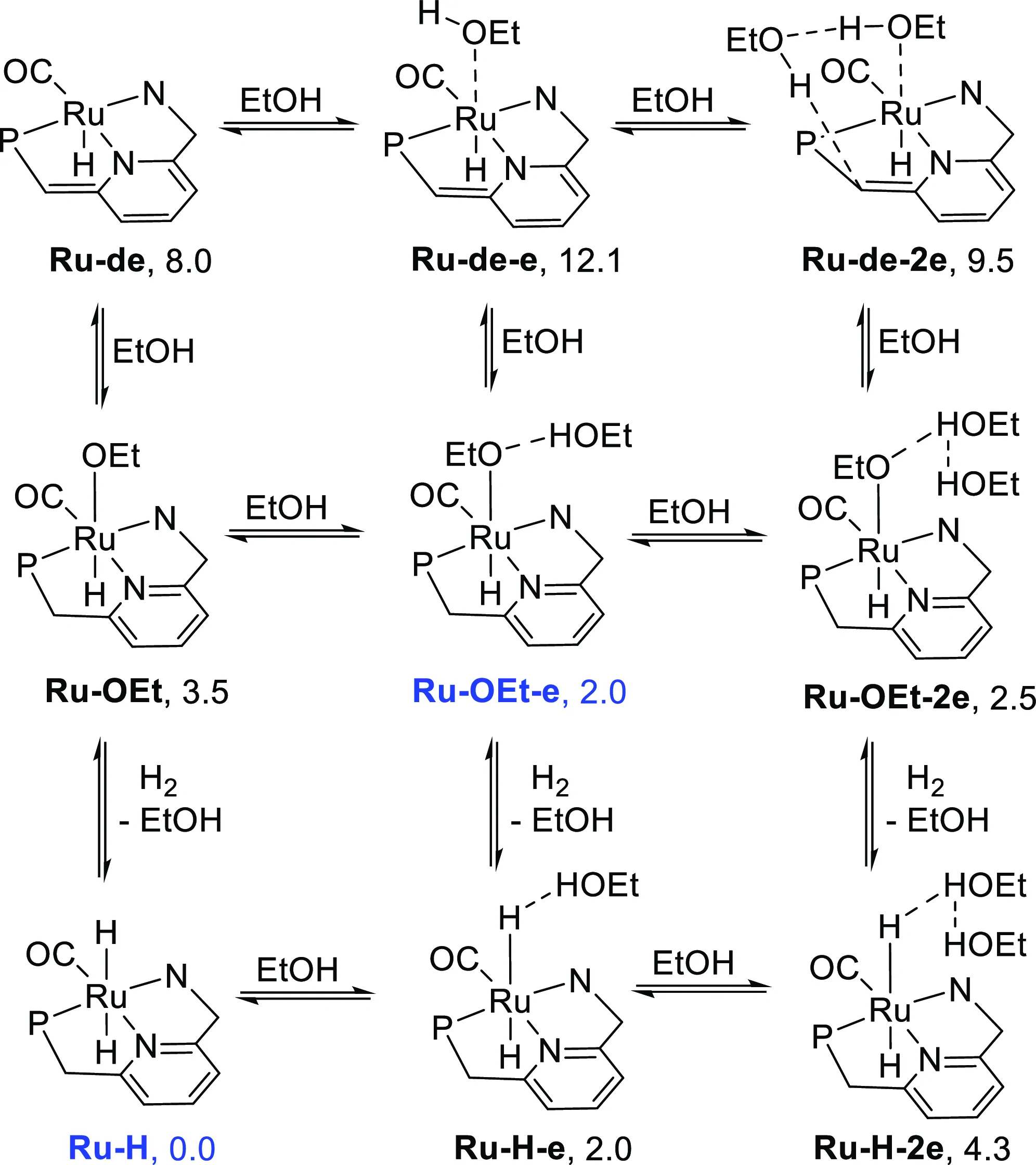
Comparison of plausible catalyst resting states, with zero, one, or two explicit ethanol molecules included. For clarity, the *t*-butyl substituents on phosphorus and the ethyl substituents on the nitrogen are omitted in Figures 6,7,8,9,10,11. The values reported are standard-state free energies in kcal/mol calculated at 55 °C.

### Activation of Hydrogen

In their original report on ester hydrogenation, Milstein and co-workers proposed that hydrogen is activated by **Ru-de** through the cooperative action of the ruthenium center as a Lewis acid and the deprotonated ligand linker carbon adjacent to phosphorus as a base. This proposal was corroborated in the computational studies of Wang^10a^ and Zhang,^14^ as well as others where related hydrogenation or dehydrogenation reactions were studied.^10c,10e,11a,11b^ In 2020, Gusev showed that hydrogen activation could proceed with a lower free-energy barrier via deprotonation of coordinated H_2_ by an exogenous alkoxide base, with no involvement of the ligand CH_2_ linkers.^15^ We have thoroughly examined these pathways including a conformational analysis of intermediates and transition states, and we find that Gusev’s mechanism is the minimum-energy pathway (MEP), as shown in Figure 7.

**Figure 7:**
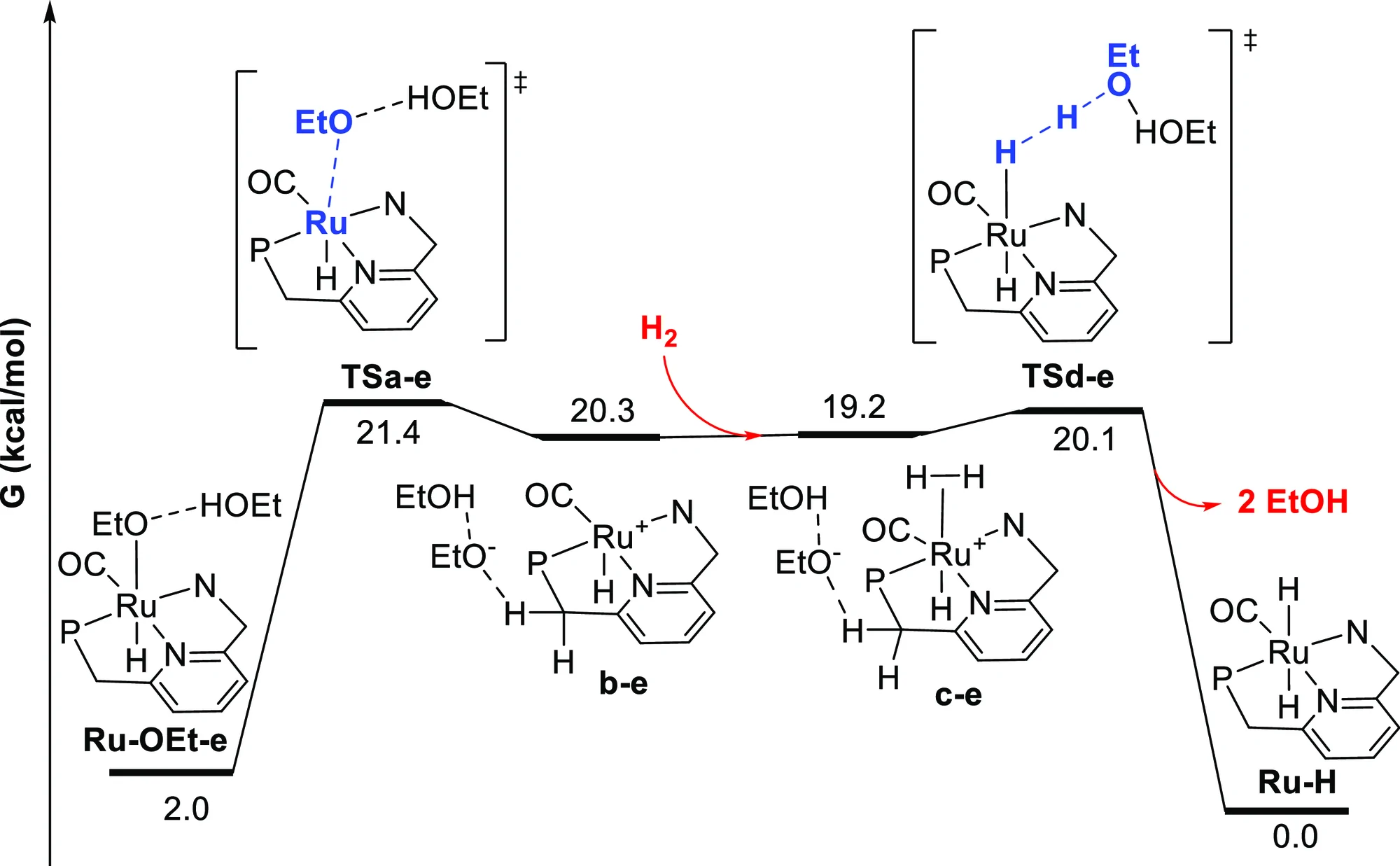
Minimum-energy pathway for the activation of hydrogen. For transition states, the atoms principally involved are highlighted in bold and blue.

Hydrogen activation begins from the hydridoethoxide complex **Ru-OEt-e**, from which the ethoxide anion dissociates through **TSa**, aided by the coordination of a molecule of ethanol, giving the ion pair **b-e**. Hydrogen coordinates to the vacant site giving **c-e**, and the coordinated H_2_ molecule is deprotonated by ethoxide to give **Ru-H** via **TSd-e**. This pathway has an overall free-energy barrier of 19.4 kcal/mol, and is not expected to be rate-determining. Based on the kinetic data and the experimental determination of resting-state speciation described above, we expected that hydrogen activation might require exogenous alcohol to proceed with a low barrier. To test this hypothesis, we attempted to recalculate the pathway shown in Figure 7 without the added explicit molecule of ethanol. As shown in Figure S1, intermediates **b** and **c**, the ethanol-free analogs of **b-e** and **c-e**, have free energies of 31.9 and 35.9 kcal/mol respectively, indicating that this pathway has a prohibitively high barrier when an alcohol molecule is not present to stabilize the concentrated negative charge of the exogenous alkoxide anion.

We also considered pathways involving metal–ligand-cooperative H_2_ activation from **Ru-de**, as previously reported by others.^14^ We find that H_2_ activation by **Ru-de** proceeds with an overall barrier of 27.9 kcal/mol from the intermediate **Ru-OEt-e**, and that this barrier is only reduced to 27.1 kcal/mol with the inclusion of an ethanol molecule as proton shuttle (Figure S2). Interestingly, metal–ligand-cooperative H_2_ activation through the CH_2_ linker on the amine side of the pincer ligand proceeds with a lower barrier of 25.9 kcal/mol (Figure S3), but this is still much higher than the MEP shown in Figure 7.

In summary, hydrogen activation proceeds via deprotonation of coordinated H_2_ by ethoxide anion, without involvement of the ligand CH_2_ groups. This pathway can only proceed with a low barrier in the presence of exogenous alcohol. This finding is consistent with our experimental observations in two key ways: (1) the low overall barrier of 19.4 kcal/mol for hydrogen activation means this portion of the mechanism lies outside of the rate-determining sequence from the TDI to the TDTS, consistent with the observed lack of dependence of the reaction rate on the hydrogen pressure; and (2) the requirement of an alcohol molecule for low-barrier hydrogen activation is consistent with the experimental finding that added alcohol is required for catalysis.

### Ester Hydrogenolysis

As described in the Introduction, the mechanism of ester hydrogenolysis mediated by Milstein’s catalyst was addressed computationally by the groups of Wang,^10a^ Hasanayn,^13^ Zhang,^14^ and Guzev.^15^ Wang and co-workers found that a pathway involving bifunctional double hydrogen transfer, with **Ru-de** as a key intermediate, was favored, with Milstein’s proposed path–involving dechelation of the NEt_2_ group followed by inner-sphere 1,2-insertion of the C=O bond into Ru–H–having a higher overall barrier.^10a^ Zhang and co-workers arrived at the same conclusion,^14^ although they considered a 1,2-insertion pathway with a different stereochemical configuration: in Wang’s work, the ester oxygen was trans to hydride, while in Zhang’s paper the ester oxygen was trans to phosphorus. Hasanayn identified a completely different mechanism for ester hydrogenolysis, termed H/OR metathesis, which does not involve dechelation or deprotonation of the pincer ligand;^13^ Gusev concluded that Hasanayn’s mechanism was preferred.^15^

Because the ester hydrogenolysis portion of the catalytic cycle is rate-determining and because such a diverse set of mechanisms has been proposed, we examined plausible pathways in great detail. Improvements on the prior work include: (1) a thorough automated conformational analysis of all potentially rate-determining intermediates and transition states using CREST;^31^ (2) consideration of multiple diastereomeric pathways, since both the ruthenium and hemiacetal carbon centers are stereogenic; and (3) comparison of all of the previously proposed pathways using the same very high-level DFT method (ωB97M-V^27^/def2-QZVPPD^28^/COSMO-RS//r^2^SCAN-3c^29^). Figures 8,9,10 below and Figures S4–S23 in the Supporting Information summarize the ester hydrogenolysis pathways we considered.

**Figure 8:**
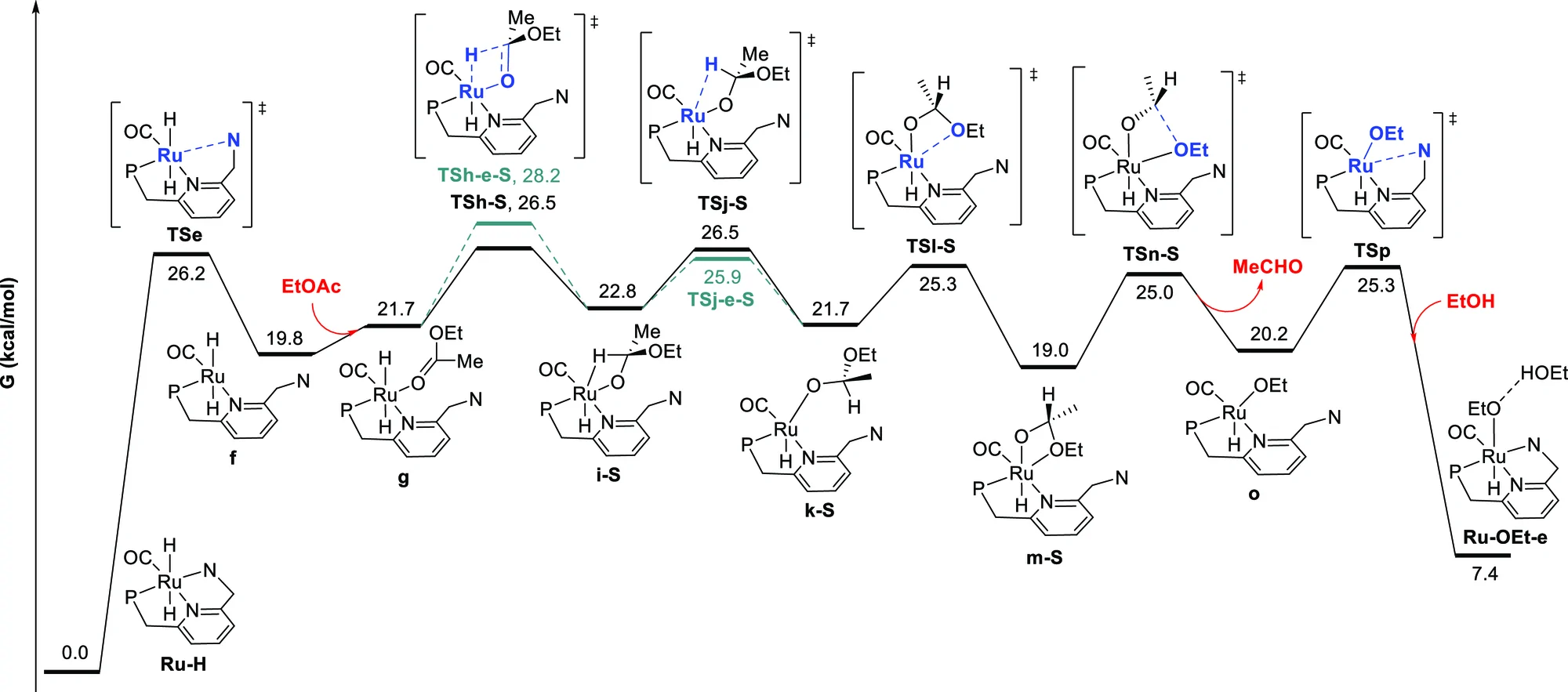
Overall minimum-energy pathway for ester hydrogenolysis. Energies in black refer to species with no explicit ethanol; energies in teal refer to the same species with one explicit ethanol molecule. Structures of the ethanol adducts **TSh-e-S** and **TSj-e-S** are omitted for clarity of the diagram.

Figure 8 shows the minimum-energy pathway for ester hydrogenolysis, according to our calculations. The sequence begins with the predominant catalyst resting state **Ru-H**, from which the NEt_2_ arm dissociates through **TSe**, giving the unsaturated intermediate **f**. The ester coordinates to give **g**, which undergoes 1,2-insertion thorough **TSh-S** to give the agostic hemiacetaloxide complex **i-S**. The agostic C–H bond dissociates through **TSj-S** to give the five-coordinate complex **k-S**, from which the ethoxy oxygen coordinates through **TSl-S** to give **m-s**. The C–O bond is cleaved through **TSn-S**, releasing the aldehyde to give **o**, which reforms the intermediate **Ru-OEt-e** by rechelation of the NE_2_ group through **TSp**. The C–H insertion portion of this pathway from **Ru–H** to **TSh-S** is similar to the insertion pathway identified by Wang,^10a^ except that the chiral carbon has an *R* configuration in Wang’s work rather than *S*. To the best of our knowledge, the second portion of this pathway, involving cleavage of the C–O bond from the bidentate hemiacetaloxide complex **m-S** via **TSn-S**, has not been previously reported. In addition to the sequence shown in Figure 8, we also examined several diastereomeric pathways (Figures S4–S11), which had higher overall free-energy barriers.

Overall, the overall free-energy barrier of 26.5 kcal/mol for MEP shown in Figure 8 agrees reasonably well with the experimentally determined barrier of 22.0 kcal/mol, and we have not identified any complete pathways for ester hydrogenolysis with a lower overall barrier. To examine the possibility that truncation of the substrate hexyl substituents to ethyl substantially affected the calculated barriers, we recalculated the free-energy difference between **Ru-H** and **TSh-S** using the experimental substrate hexyl hexanoate; the calculated Δ*G*° of 26.0 kcal/mol (see Figure S28) indicates that this truncation has a minimal effect on the calculated energies as expected. Importantly, the overall barrier was not lowered by the inclusion of an explicit ethanol molecule, as shown in the key transition states **TSh-e-S** and **TSj-e-S**. This is consistent with the experimental observation (Figure 4, panel d) that saturation behavior is observed in the concentration of added alcohol: alcohol is required to allow the non-rate-determining hydrogen activation and aldehyde hydrogenolysis to proceed, but is not required for (and should not accelerate) the rate-determining ester-hydrogenolysis portion of the mechanism.

Figure 9 shows the lowest-energy stereoisomer of the Hasanayn H/OR metathesis pathway that we identified. In this sequence, **Ru-H** attacks the ester through **TSq-R** to give the hemiacetaloxide intermediate **r-R**, which slips through **TSs-R** to allow the ethoxy group to coordinate to ruthenium. Without an explicit ethanol molecule, C–O cleavage is concerted with slippage in **TSs-R**, leading directly to release of aldehyde and regenerating the alkoxide intermediate **Ru-OEt-e**. If an explicit ethanol molecule is included, the overall barrier is substantially lowered from 32.7 to 27.5 kcal/mol, and the slippage (through **TSs-e-R**) and C–O cleavage (through **TSu-e-R**) now occur in a stepwise fashion. In addition to the sequence shown in Figure 9, we also considered the addition of a second explicit ethanol molecule (Figure S12) and a diastereomeric pathway (Figure S13), both of which had higher overall barriers.

**Figure 9:**
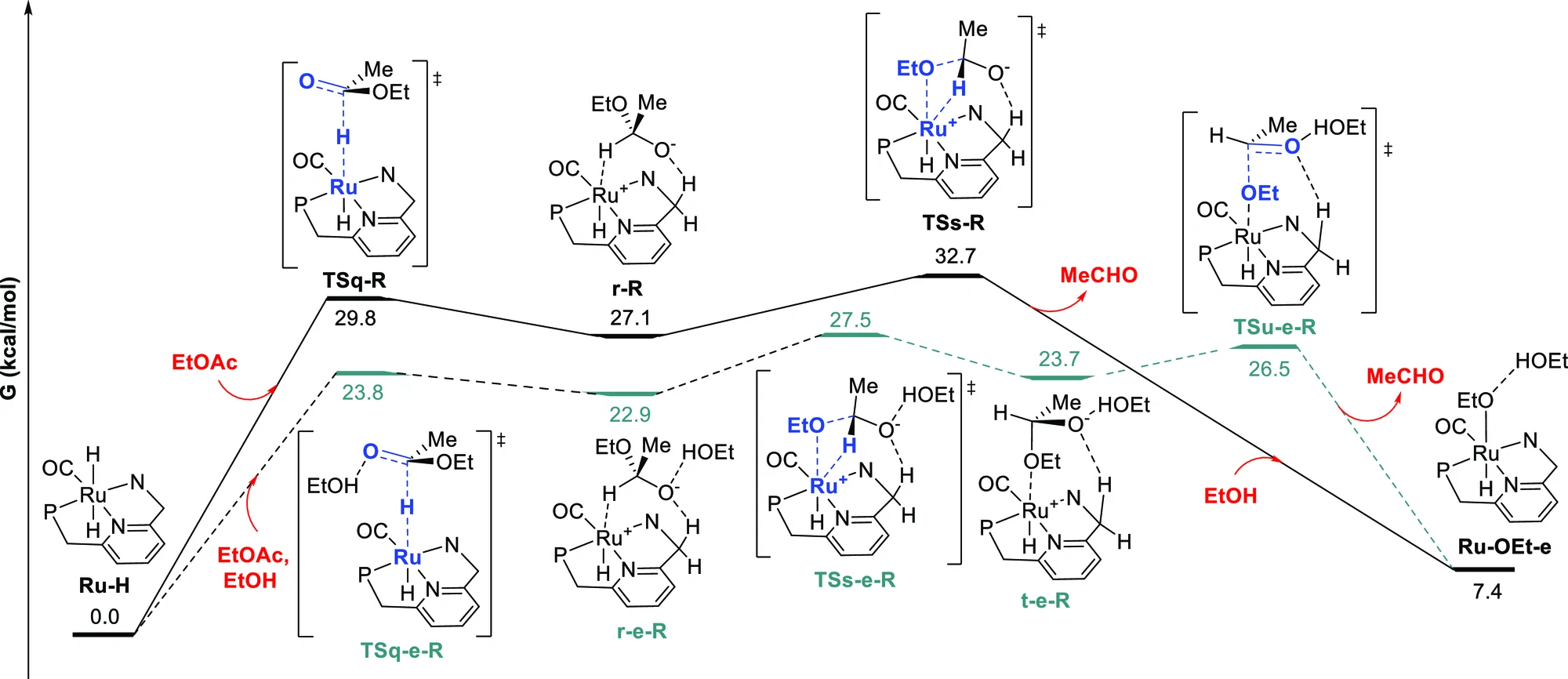
Alternative pathway for ester hydrogenolysis by an H/OR metathesis mechanism. Energies shown in teal refer to species with an explicit ethanol molecule.

Figure 10 shows the lowest-energy stereoisomer we identified for a pathway involving dearomatization of the pincer ligand. In this mechanism, C–H bond formation again proceeds through attack of **Ru-H** on the ester through **TSq-R** giving the intermediate **r-R**, but a different slippage motion in **TSv-R** allows the anionic hemiacetaloxide oxygen to coordinate to ruthenium giving **w-R**. From this point, protonation of the ethoxy oxygen by the PCH_2_ linker through **TSx-R** gives **Ru-de** with release of the aldehyde, and the intermediate **Ru-OEt-e** is formed by reaction of **Ru-de** with ethanol. The latter portion of this pathway, beginning with **w-R**, was previously identified by Wang^10a^ and Zhang.^14^ If an explicit ethanol molecule is included in this pathway, the overall barrier is lowered from 33.4 to 28.0 kcal/mol. In addition to the sequence shown in Figure 10, we also considered a diastereomeric pathway (Figure S14), an alternative mechanism for aldehyde dissociation that bypasses ligand dearomatization to regenerate **Ru-OEt-e** without the intermediacy of **Ru-de** (Figure S15), alternatives involving release of the free hemiacetal (Figures S16,S17), alternatives where the ethoxy oxygen rather than the aldehyde oxygen is coordinated to ruthenium during C–O cleavage (Figures S18,S19), and variants where the NCH_2_ linker is deprotonated instead of the PCH_2_ linker (Figures S20–S23). All of these alternative mechanisms had higher overall barriers compared with that shown in Figure 10.

**Figure 10:**
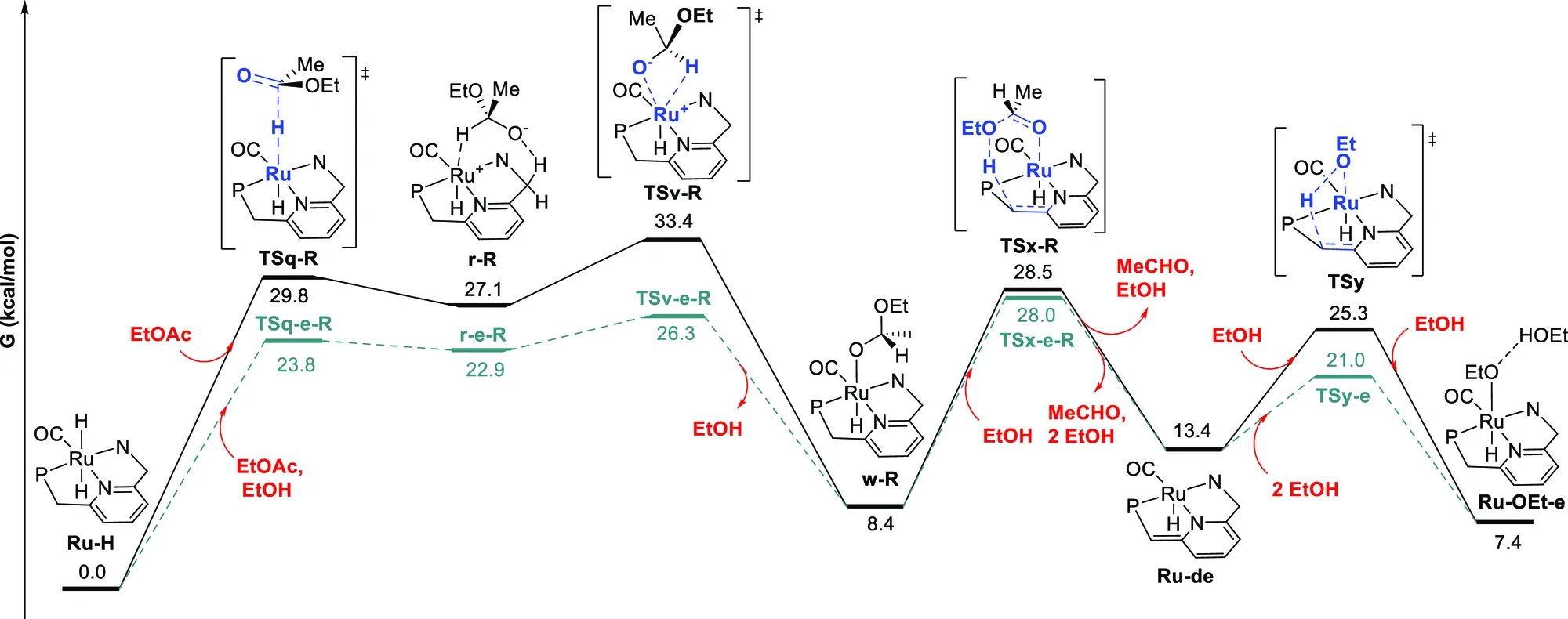
Alternative pathway for ester hydrogenolysis involving dearomatization of the pincer ligand. Energies shown in teal refer to species with an explicit ethanol molecule. Structures of the ethanol adducts are omitted for clarity of the diagram.

The three disparate pathways for ester hydrogenolysis discussed above are difficult to distinguish using DFT alone, as the overall barriers are 26.5, 27.5, and 28.0 kcal/mol. These differences are small enough to be within the expected error for calculations of solution free-energies using DFT. However, the experimental kinetics clearly show that added alcohol does not increase the reaction rate significantly once the saturation threshold of approximately 0.1 M is reached. This observation supports a rate-determining sequence where the addition of an explicit alcohol molecule between the TDI and the TDTS does not substantially lower the energetic span. The pathway shown in Figure 8 is consistent with this requirement, while the pathways in Figures 9 and 10 are not. Although the assignment is not completely unambiguous, we believe that the dechelation/insertion pathway shown in Figure 8 is most likely operative based on its agreement with the kinetic data.

### Aldehyde Hydrogenation

The final portion of the bicyclic catalytic pathway shown in Scheme 4 is the hydrogenation of the aldehyde released during ester hydrogenolysis. As we did for ester hydrogenolysis, we considered pathways involving pincer ligand dechelation/inner-sphere insertion, direct hydride attack followed by slippage to generate a ruthenium ethoxide complex, and pincer ligand dearomatization.

Figure 11 shows the minimum-energy pathway we identified for aldehyde hydrogenation, which requires an explicit molecule of ethanol to proceed with a low barrier and was previously identified by Gusev.^15^ First, **Ru-H** attacks the carbonyl carbon through **TSz-e** giving **aa-e**, a C–H σ-complex of ethoxide. The ethoxide anion then rearranges through **TSab-e** to give **Ru-OEt-e**, completing the catalytic cycle. This sequence has an overall free-energy barrier of 14.0 kcal/mol from the intermediate **Ru-H**, indicating that it is not rate-determining. We examined the same pathway without an explicit ethanol molecule to stabilize the negative charge of the anionic ethoxide oxygen through hydrogen bonding, and the barrier was increased substantially to 22.6 kcal/mol (Figure S24). We considered two diastereomeric pathways previously examined by Wang^10a^ and Zhang^14^ involving pincer ligand dechelation followed by 1,2-migratory insertion of the C=O double bond into the Ru–H bond (Figures S25 and S26), both of which had much higher barriers than the MEP shown in Figure 11. Additionally, we considered a pathway (Figure S27) where the ethoxide σ-complex **aa-e** (or **aa** without an explicit ethanol molecule) releases ethanol by proton transfer from the pincer ligand’s CH_2_ linker as previously examined by Wang^10a^ and Zhang,^14^ which also proceeded with a higher barrier. In summary, the hydrogenation of the aldehyde occurs through direct hydride attack and rearrangement to the alkoxide complex as shown in Figure 11. The nonrate-determining aldehyde hydrogenation sequence requires an explicit alcohol molecule to occur with a low barrier, which is again consistent with the experimental kinetic data, which showed added alcohol is required for catalytic activity but shows saturation behavior.

**Figure 11:**
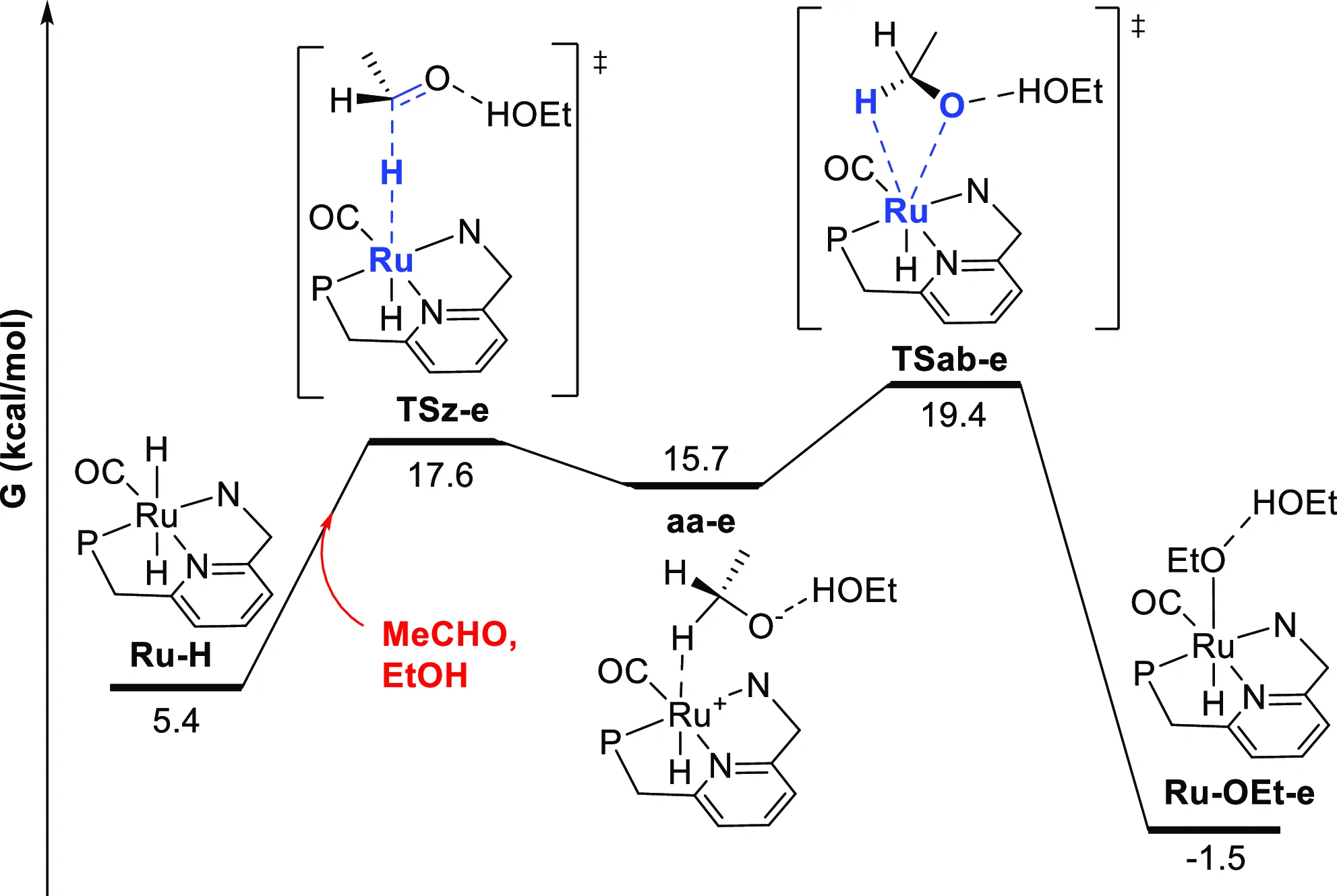
Minimum-energy pathway for aldehyde hydrogenation.

## Discussion

The experimental and computational results described above comprise the most comprehensive assessment to date of the mechanism of ester hydrogenation catalyzed by **Ru-de**, including an exhaustive DFT analysis backed by key experimental measurements. Experimental checks include (1) the determination of the overall catalytic rate law, which is fully consistent with the kinetics predicted by DFT; (2) the experimental determination of the overall free-energy barrier (energetic span) of 22.0 kcal/mol, which is reasonably consistent with the calculated overall barrier of 26.5 kcal/mol; and (3) the experimental analysis of the catalyst resting state equilibrium by NMR.

Overall, our proposed mechanisms for hydrogen activation and aldehyde hydrogenation are consistent with those proposed recently by Gusev.^15^ For these portions of the catalytic pathway, the free-energy barriers for alternative mechanisms involving pincer ligand dearomatization or dechelation are substantially higher than the minimum-energy pathways shown in Figures 7 and 11. Both hydrogen activation and aldehyde reduction rely on the well-documented ability of alcohols to serve as proton shuttles^20b,21^ in catalytic reactions, as has been demonstrated in many computational studies of catalysts for polar-bond hydrogenation and related reactions.^24,32^ Additionally, Milstein and co-workers have demonstrated that thiols can have a similar beneficial effect.^33^

For the rate-determining ester hydrogenation sequence, we find that the minimum-energy pathway involves dechelation of the pincer’s NEt_2_ arm and 1,2-insertion of the C=O bond into Ru–H, as shown in Figure 8. Alternative pathways involving H/OR metathesis (Figure 9) as proposed by Hasanayn^13^ and Gusev,^15^ or dearomatization of the pincer ligand (Figure 10) as proposed by Wang^10a^ and Zhang,^14^ were found to have slightly higher overall barriers in our analysis. Importantly, these alternative pathways would predict a first-order dependence of the catalytic rate on the alcohol concentration, which is not consistent with the observed kinetic data (Figure 4).

### Effect of Solvent

Because the experimental work we report here was conducted in toluene, we have used toluene as a solvent model in the DFT calculations discussed above (CPCM for geometry optimizations and frequency calculations; COSMO-RS for single-point energies). Milstein’s original work was conducted in dioxane,^4^ although without added alcohol at the outset it is likely that productive catalysis occurred through **Ru-NH** after ethane release (Scheme 3) rather than **Ru-H** formed by reaction of **Ru-de** with hydrogen. Gusev observed dramatically higher catalytic activity when ethanol was used as solvent,^15^ although **Ru-NH** again may be the predominant active catalyst form, since reactions were run at 100 °C where ethane release to form **Ru-NH** is known to be fast.^17^ Still, prior computational work on ester hydrogenation by **Ru-de** used a variety of solvent continuum models: Wang used CPCM/toluene,^10a^ Hasanayn used CPCM/THF,^13^ Zhang used SMD/dioxane and identified a change in mechanism in more polar solvents,^14^ and Gusev used SMD/toluene.^15^ To assess the effect of the chosen solvent on the free energies calculated by DFT, we calculated free energies of solvation in tetrahydrofuran and ethanol using the COSMO-RS model. Table 1 (top) shows the overall barrier for the preferred pathway and alternatives in these three solvents. Values in bold and blue represent the preferred pathway for each method, and values in parentheses represent the overall barrier if all species involving explicit ethanol are excluded. For hydrogen activation, the pathway where ethoxide deprotonates coordinated H_2_ (Figure 7) consistently has a lower barrier than alternatives involving dearomatization (Figures S2,S3). For ester hydrogenolysis, the dechelation/inner-sphere insertion pathway (Figure 8) remains preferred in each of the three solvents tested, compared with pathways involving H/OR metathesis (Figures S12 and S13), and pathways involving ligand dearomatization (Figures S14–S23). Satisfyingly, the effect of including an explicit ethanol molecule is essentially eliminated for all three pathways when the COSMO-RS solvent model for ethanol is used. For aldehyde hydrogenation, the pathway involving hydride attack followed by slippage (Figure 11) remains the MEP in all three solvents, compared with pathways involving pincer dechelation (Figures S25 and S26) or dearomatization (Figure S27).

**Table 1:**
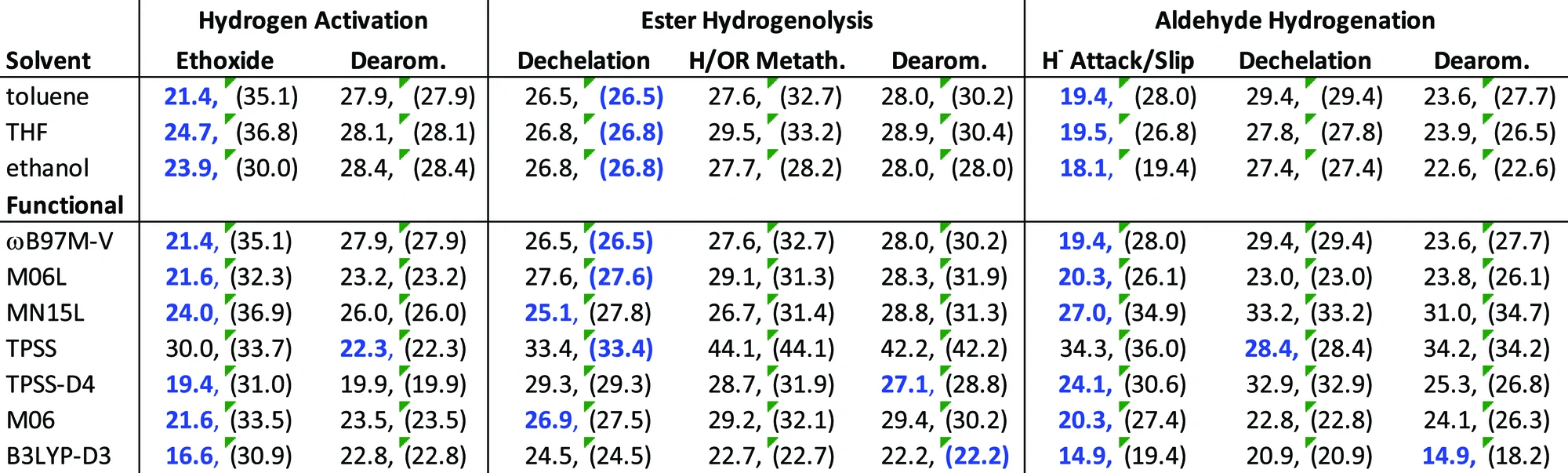
Effect of Solvent and Functional on the Overall Barriers for Alternative Pathways^a^

a Values without parentheses are the overall free-energy maximum in kcal/mol for each pathway; values in parentheses are the overall maxima with no explicit ethanol. Values in bold and blue represent the minimum-energy pathway for each method.

### Effect of the Choice of Functional

To assess the effect of the chosen density functional approximation on the free energies, the single-point electronic energies were recalculated using the following functionals: M06L,^34^ MN15L,^35^ TPSS,^36^ TPSS-D4, M06,^37^ and B3LYP-D3,^38^ in each case using the same def2-QZVPPD basis set and toluene COSMO-RS solvent model. These functionals were chosen to provide a comparison to the methods used in previous DFT analyses. In his 2020 paper,^15^ Gusev used the *meta*-GGA functionals M06L and MN15L, which account for dispersion implicitly through their parametrization. In their 2011 paper,^10a^ Wang and co-workers used the *meta*-GGA functional TPSS, apparently without an empirical dispersion correction. Because, as shown above in Table 1, this method gives energies that are substantially out of agreement with the others considered here, we have additionally calculated electronic energies using TPSS with the charge-dependent D4 dispersion correction recently developed by Grimme and co-workers.^39^ In their 2013 paper,^13^ Hasanayn and Baroudi used the hybrid functional M06, which again accounts for dispersion implicitly. In their 2017 paper,^14^ Zhang and co-workers used the B3LYP hybrid functional with Grimme’s D3 ^40^ empirical dispersion correction applied. Although a variety of double- and triple-ζ basis sets were used in the prior work, we have not attempted to examine the effect of using less complete basis sets on the calculated energies.

The lower portion of Table 1 above shows the overall energy barriers for the minimum-energy and alternative pathways for each of the three portions of the catalytic cycle. Because multiple diastereomeric options were considered for each class of mechanism, the precise MEP within each class varies as described in the Supporting Information. First, we note that TPSS without a dispersion correction predicts substantially higher barriers than all other methods for many of the main and alternative pathways, which are far out of line with experiment. This arises from a general penalty for bringing molecules together when no dispersion correction is applied. TPSS with the D4 dispersion correction gives overall barriers more closely aligned with the other functionals, all of which account for dispersion either explicitly or implicitly.

For the hydrogen activation and aldehyde hydrogenation sequences, all functionals tested aside from TPSS predict the same MEPs, matching those in Figures 7 and 11, respectively. One exception is that in the aldehyde hydrogenation sequence, B3LYP-D3 predicts the same barrier of 14.9 kcal/mol for the hydride attack/slippage mechanism and the mechanism involving ligand dearomatization.

For the ester hydrogenolysis sequence, where ωB97M-V predicts that the preferred pathway involves pincer ligand dechelation, the two alternative pathways involving H/OR metathesis and ligand dearomatization are only 1.1 and 1.5 kcal/mol higher in free energy, respectively. Four of the alternative functionals tested (M06L, MN15L, TPSS, and M06) are in agreement, while two others (TPSS-D4 and B3LYP-D3) predict that the dearomatization sequence is slightly preferred. These results emphasize that for disparate pathways with such close overall barriers, DFT alone cannot provide an unambiguous assignment of the preferred mechanism. Still, it is encouraging that ωB97M-V, which has consistently performed excellently in recent benchmarking studies,^26b–26d^ gives results consistent with the experimental observation that an alcohol cocatalyst is required for non-rate-determining portions of the mechanism but not for the rate-determining ester hydrogenolysis sequence.

### Broader Implications

It is worth revisiting the broader implications of this work for our understanding of how Milstein’s catalyst **Ru-de**, which forms **Ru-H** under hydrogen, operates in ester hydrogenation. Although in this article we focus on the mechanism of action of Milstein’s catalyst in its original form with the NEt_2_ group intact at 55 °C, **Ru-de** is known to release ethane at temperatures as low as 80 °C to transform into the much more active **Ru-NH**, as shown in Scheme 3.^17^ Our previous comprehensive experimental and computational analysis of the catalytic mechanism for **Ru-NH** ^18^ showed key differences when compared to **Ru-H**, which are described below.

First, the overall (experimentally determined) free-energy barrier for the hydrogenation of hexyl hexanoate catalyzed by **Ru-NH** is 17.4 kcal/mol, much lower than the 22.0 kcal/mol experimental barrier for **Ru-H**. As a result, **Ru-NH** is highly active at 25 °C, while **Ru-H** is weakly active at 55 °C. Second, the reaction of **Ru-NH** with alcohol to give a ruthenium-alkoxide species is highly thermodynamically favorable, while the same reaction (Figure 5) is slightly unfavorable for **Ru-H**. This means that the ruthenium-alkoxide species is the dominant resting state for **Ru-NH**, while the dihydrido species is predominant for **Ru-H**. Third, and because of the change in resting state, the kinetic behavior of the two catalysts is quite distinct. For **Ru-NH**, the catalytic rate shows a first-order dependence on [Ru] and the hydrogen pressure, saturation in [ester], and inhibition by added primary alcohol, consistent with hydrogen activation lying on the rate-determining reaction sequence.^18^ For **Ru-H**, the catalytic rate shows a first-order dependence on [Ru] and [ester], no effect of the hydrogen pressure, and saturation kinetics in [alcohol]. Fourth, the simple change of one NEt group to NH has profound implications for the catalytic mechanism. In contrast to the mechanism reported here for **Ru-H** where the pincer ligand is only actively involved because of the ability of the NEt_2_ group to dechelate as shown in Figure 8, the secondary amine functional group of **Ru-NH** is intimately involved in the mechanisms of hydrogen activation, ester hydrogenolysis, and aldehyde hydrogenation, as described in our previous work.^18^ The key role of the NH group in lowering the barrier is consistent with the broader observation that elite catalysts for ester hydrogenation tend to feature a ligand N–H group.^32f,41^ On the other hand, Dub, Scott, and Gordon^32d^ have demonstrated that Noyori-type ruthenium catalysts for hydrogenation can maintain or even improve their catalytic activity when their N–H group is alkylated.

## Conclusion

In summary, we report the surprising finding that Milstein’s catalyst **Ru-de**, previously shown to convert to a highly active form at elevated temperatures by releasing ethane,^17^ is also moderately active in its original form provided exogenous alcohol is present to stabilize key transition states in the hydrogen activation and aldehyde hydrogenation sequences. We have completed a comprehensive experimental/computational study, including experimental determination of resting state speciation, a complete kinetic analysis, and an exhaustive DFT study of the reaction mechanism. Work to determine the mechanism of ethane release by **Ru-de** is ongoing in our laboratory.

## Methods

### General Experimental Methods

Toluene was purchased in anhydrous form from EMD-Millipore and was deoxygenated by sparging with argon before bringing into the glovebox. Liquid reagents were similarly sparged with argon before bringing into the glovebox. To remove trace carboxylic acid impurities, hexyl hexanoate was filtered through activated basic alumina before use. Hydrogen gas was purchased from Airgas at the Ultrahigh Purity level. **Ru-de** was synthesized as described previously.^3^ Gas chromatography was conducted using a Shimadzu 2030 system equipped with an FID detector.

### Hydrogenation of Dimethyl Carbonate

The hydrogenation of dimethyl carbonate, as shown in Figure 1, was conducted in the customized 100 mL Parr reactor described in the Supporting Information. The oil bath was preheated to 100 °C in a fume hood. The disassembled reactor, along with an oven-dried glass liner, was brought into the glovebox. Dimethyl carbonate (0.901 g, 10.0 mmol), the appropriate ruthenium complex (0.050 mmol), and mesitylene (20 μL) as internal standard were added to a 25.0 mL oven-dried volumetric flask, which was then filled to the line with toluene. The reaction solution was transferred to the glass liner along with a stir bar, and the reactor was assembled and sealed. The reactor was removed from the glovebox. After purging gently for 2 min, the hydrogen line was connected and the reactor was pressurized to 20 bar. The thermocouple was connected to the temperature controller, which was set to 80 °C. The reactor was immersed in the oil bath, and the temperature was recorded each minute. Time zero was marked when the internal temperature reached 75 °C, typically after 5–10 min. Liquid and headspace aliquots were removed at regular intervals. Headspace aliquots were analyzed by GC as described above, and liquid aliquots were analyzed by ^1^H NMR spectroscopy in CDCl_3_, comparing the integrations of the signals from dimethyl carbonate with those from the standard mesitylene.

### Dehydrogenation of 1-Decanol

The dehydrogenation of 1-decanol, as shown in Figure 2, was conducted in the customized 100 mL Parr reactor described in the Supporting Information. The oil bath was preheated to 105 °C in a fume hood. The disassembled reactor, along with an oven-dried glass liner, was brought into the glovebox. 1-Decanol (0.396 g, 2.50 mmol), the appropriate ruthenium complex (0.050 mmol), and 1,3,5-trimethoxybenzene (20 mg) were added to a 25.0 mL oven-dried volumetric flask, which was then filled to the line with toluene. The reaction solution was transferred to the glass liner along with a stir bar, and the reactor was assembled and sealed. The reactor was removed from the glovebox. After purging gently for 2 min, the argon line was connected and the reactor was pressurized to 4 bar. The thermocouple was connected to the temperature controller, which was set to 85 °C. The reactor was immersed in the oil bath, and the temperature was recorded each minute. Time zero was marked when the internal temperature reached 80 °C, typically after 5–10 min. Liquid and headspace aliquots were removed at regular intervals. Headspace aliquots were analyzed by GC as described above, and liquid aliquots were analyzed by ^1^H NMR spectroscopy in CDCl_3_, comparing the integrations of the signals from 1-decanol and the product decyl decanoate with those from the standard 1,3,5-trimethoxybenzene.

### Hydrogenation of Hexyl Hexanoate with Headspace Monitoring

The hydrogenation of hexyl hexanoate, as shown in Figure 3, was conducted in the customized 100 mL Parr reactor described in the Supporting Information. The oil bath was preheated to 105 °C in a fume hood. The disassembled reactor, along with an oven-dried glass liner, was brought into the glovebox. Hexyl hexanoate (2.00 g, 10.0 mmol), the appropriate ruthenium complex (0.050 mmol), 1,3,5-trimethoxybenzene (20 mg) and optionally 1-hexanol (0.102 g, 1.00 mmol) were added to a 25.0 mL oven-dried volumetric flask, which was then filled to the line with toluene. The reaction solution was transferred to the glass liner along with a stir bar, and the reactor was assembled and sealed. The reactor was removed from the glovebox. After purging gently for 2 min, the hydrogen line was connected and the reactor was pressurized to 20 bar. The thermocouple was connected to the temperature controller, which was set to 85 °C. The reactor was immersed in the oil bath, and the temperature was recorded each minute. Time zero was marked when the internal temperature reached 80 °C, typically after 5–10 min. Headspace aliquots were analyzed by GC as described above, and liquid aliquots were analyzed by ^1^H NMR spectroscopy in CDCl_3_, comparing the integrations of the signals from hexyl hexanoate and the product 1-hexanol with those from the standard 1,3,5-trimethoxybenzene.

### Hydrogenation of Hexyl Hexanoate for Kinetic Study

In a fume hood, the oil bath described above was preheated to 70 °C. The Asynt Parallel Reactor described in the Supporting Information, along with oven-dried glass liners for each reaction, were brought into the glovebox. Reaction solutions (total volume 10.0 mL) were prepared with the appropriate amounts of **Ru-de**, hexyl hexanoate, 1-hexanol, and tetradecane (internal standard, 0.20 equiv. relative to the ester) in toluene, and were transferred to individual reaction vessels. After the reactor was closed and sealed, it was brought out of the glovebox. After gently purging the hydrogen line for 2 min, it was connected to the reactor and the reactor was filled to the appropriate pressure (10–30 bar). The hydrogen line was kept connected to the reactor throughout the reaction time to ensure that the desired pressure was maintained as aliquots were removed. The thermocouple was connected, the temperature controller was set to 55 °C, and the reactor was immersed in the oil bath. The temperature was recorded every minute, and time zero was marked when it first reached 50 °C, typically after 5–10 min. Aliquots were removed at regular intervals as shown below. The conversion of hexyl hexanoate was monitored by GC analysis, comparing the integration of the substrate signal with that of the internal standard tetradecane. GC analysis employed a Shimadzu SH-Rxi-5 ms column 15 m in length. The oven temperature was initially held at 50 °C for 3 min, then increased to 280 °C at a rate of 20 °C/min, then held at 280 °C for 5 min. With these parameters, 1-hexanol eluted at 3.52 min, hexyl hexanoate eluted at 8.58 min, and tetradecane eluted at 8.67 min.

### NMR Study of Catalyst Speciation

The NMR study of catalyst resting state speciation depicted in Figure 5 consists of six experiments with varying concentrations of 1-hexanol and varying hydrogen pressure. For each experiment, 5.5 mg of **Ru-de** was dissolved in toluene-d_8_ in the glovebox with 1-hexanol added to give molarities ranging from 0.44 to 1.39 M, each with a total volume of 0.70 mL. The solutions were transferred to J-Young NMR tubes, sealed, and removed from the glovebox. They were then briefly evacuated, and pressurized with 1–2 bar hydrogen gas and sealed. ^1^H NMR spectra were recorded at 55 °C. The ratios [**Ru-H**]/[**Ru-alkoxide**] and [H_2_]/[1-hexanol] were measured by integration of the relevant signals: For **Ru-H**, the 2H hydride signal at −4.7 ppm was used; for **Ru-alkoxide**, the 1H hydride signal at −16.5 ppm was used; for H_2_, the signal at 4.5 ppm was used; and for 1-hexanol, the 3H methyl group signal at 0.9 ppm was used.

### Computational Methods

Density functional theory calculations were performed using the ORCA computational chemistry package, version 6.0.1.^42^ The geometries and energies of all species were calculated using the r^2^SCAN-3c composite method developed by Grimme and coco-workers,^29^ using the CPCM^43^ continuum solvent model for toluene. For improved convergence of geometries, the DEFGRID3 integration grid was used for all calculations, along with the TIGHTSCF keyword to achieve tight SCF convergence. Frequency calculations ensured the absence of imaginary vibrational modes in intermediates and the presence of exactly one imaginary mode in transition states. Intrinsic reaction coordinate calculations were used to verify that transition states led to the specified minima. Free-energy corrections were calculated at the experimental reaction temperature of 55 °C, or 328.15 K. Standard-state corrections were applied in order to adjust from 1 atm to 1 M for solution-phase free energies, amounting to 2.15 kcal/mol added to the free energy of each isolated molecule at 328.15 K.^44^ Although the standard state for molecular hydrogen is sometimes taken as the gas at 1 atm, we have used a 1 M standard state in toluene solution, for consistency in computing reaction kinetics from the calculated free energies. The electronic energies were further refined using the ωB97M-V range-separated hybrid functional from Head-Gordon and co-workers,^27^ along with the def2-QZVPPD basis set.^28^ These electronic energies were calculated in vacuum, and separately the free energy of solvation in toluene at 328.15 K was calculated using the COSMO-RS^30^ model with default recommended method in ORCA 6.0.1 (BP86/def2TZVPD). For species likely to be a rate-determining intermediate or transition state, a conformational analysis was conducted using the CREST program developed by Grimme and coco-workers.^31^ All energies reported in the paper are standard-state free energies at 328.15 K. A table of energies is provided in the Supporting Information, and geometries in Cartesian coordinates are included in a separate, compiled.XYZ file. In addition to the method described above, we also recalculated single point electronic energies using different solvents (THF and ethanol) and different density functionals (M06L,^34^ MN15L,^35^ TPSS,^36^ TPSS-D4, M06,^37^ and B3LYP-D3 ^38^).

## Supplementary Material





## Notes

Atomic coordinates (XYZ) for all computed molecular structures, compiled as one file (DFT_structures.xyz), readable by the free program Mercury^45^ (XYZ)
Additional experimental and computational details (PDF) (PDF)
