# Supplementary material for: Dual Active Forms of Milstein’s Catalyst for Ester Hydrogenation
Source: Organometallics. 2026 Aug 5;45(16):1983–97. doi: 10.1021/acs.organomet.6c00183 (PMC13509128; doi:10.1021/acs.organomet.6c00183)
Supplement: Supplementary file 1 [file om6c00183_si_002.pdf]

Supporting Information for

# Dual Active Forms of Milstein's Catalyst for Ester Hydrogenation

Haitian Ye, Jason M. Keith*, and Anthony R. Chianese*

Department of Chemistry, Colgate University, 13 Oak Drive, Hamilton, New York 13346, United States

Emails: J. M. Keith, jkeith@colgate.edu; A. R. Chianese, achianese@colgate.edu

**Table of Contents**

Apparatus for Kinetic Experiments with Headspace Monitoring ....................2
Numerical Data for the Hydrogenation of Dimethyl Carbonate ....................3
Numerical Data for the Dehydrogenation of 1-Decanol ....................3
Numerical Data for the Hydrogenation of Hexyl Hexanoate with Headspace Monitoring ....................4
Apparatus for Kinetic Experiments in Parallel ....................4
Numerical Data for Hydrogenation of Hexyl Hexanoate in the Asynt Parallel Reactor ....................5
Determination of Overall Free-Energy Barrier for Ester Hydrogenation ....................8
Numerical Data for the NMR study of catalyst speciation ....................8
Energies calculated by DFT ....................9
Alternative Pathways for Hydrogen Activation ....................17
Alternative Pathways for Ester Hydrogenolysis Involving Dechelation and Inner-sphere Insertion ....................18
Alternative Pathways for Ester Hydrogenolysis Involving CH/OR Metathesis as Proposed by Hasanayn ....................22
Alternative Pathways for Ester Hydrogenolysis Involving Dearomatization of the Pincer Ligand ....................23
Alternative Pathways for Aldehyde Hydrogenation ....................28
Recalculation of the Energetic Span using Hexyl Hexanoate ....................31
Variation of the Specific Minimum-Energy Pathway by Functional Within Each Class of Mechanism ....................31
References ....................42

**Apparatus for Kinetic Experiments with Headspace Monitoring**

For kinetic experiments where substrate conversion was monitored in parallel with ethane release by the catalyst (Figures 1-3), a customized 100 mL stainless steel reactor from Parr was used. Both liquid and headspace aliquots were collected using 0.8 mm ID 1/16” stainless steel tubing, a Swagelok Low Flow metering valve (part no. SS-SS1) to control flow, and a Swagelok ball valve (part no. SS-41GS1) to allow removal of samples. For liquid aliquots, the sampling tubing extended into the liquid portion of the reaction mixture, approximately 5 mm from the bottom of the reactor. As the internal volume of the sampling valve system was measured to be 0.25 mL, removal of 0.50 mL of liquid before collecting each aliquot ensures that a fresh sample is being taken directly from the reaction mixture. For headspace aliquots, the sampling tubing was terminated above the liquid level inside the reactor. Outside of the reactor, the sampling tubing was run into a glass recrystallization dish filled with water, and gas samples were collected using GC vials initially filled with water, which were inverted and placed over the sampling tube to collect gas, and subsequently capped underwater. For each aliquot, a 5 mL vial was first filled with gas which was discarded, as the ~0.25 mL internal volume of the sampling valve expands by 20-fold after decompression from 20 bar to atmospheric pressure. Then, a 1.5 mL GC vial was filled with gas and analyzed directly by GC-FID, with an injection volume of 5 μL (an autosampler made the injections, the volumes of which are precise within ~1%). Ethane was detected using a 25 meter Agilent CP-$Al_2O_3$/$Na_2SO_4$ column, part no. CP7586. The GC method ran for 3.0 minutes at 50 °C. Ethane eluted with a retention time of 1.12 minutes. Liquid aliquots were analyzed depending on the substrate as described below.

### Numerical Data for the Hydrogenation of Dimethyl Carbonate

**Table S1.** Numerical data for the hydrogenation of dimethyl carbonate by **Ru-de** and **Ru-imine**, as shown in graphical form in Figure 1.

| Ru-de | | | Ru-imine | | |
|---|---|---|---|---|---|
| t (min) | [DMC] (M) | ethane signal | t (min) | [DMC] (M) | ethane signal |
| 0 | 0.400 | 0 | 0 | 0.400 | 0 |
| 15 | 0.395 | 57 | 3 | 0.371 | 0 |
| 30 | 0.385 | 112 | 18 | 0.306 | 0 |
| 45 | 0.369 | 143 | 33 | 0.249 | 0 |
| 60 | 0.359 | 156 | 48 | 0.200 | 0 |
| 90 | 0.309 | 184 | 63 | 0.163 | 0 |
| 120 | 0.283 | 199 | 93 | 0.111 | 0 |
| 150 | 0.260 | 204 | 123 | 0.080 | 0 |
| 180 | 0.243 | 196 | 153 | 0.059 | 0 |
| 210 | 0.228 | 183 | 183 | 0.047 | 0 |
| 240 | 0.219 | 179 | 213 | 0.037 | 0 |
| | | | 243 | 0.030 | 0 |

### Numerical Data for the Dehydrogenation of 1-Decanol

**Table S2.** Numerical data for the dehydrogenation of 1-decanol by **Ru-de** and **Ru-imine**, as shown in graphical form in Figure 2.

| Ru-de | | | Ru-imine | | |
|---|---|---|---|---|---|
| t (min) | [1-decanol] (M) | ethane signal | t (min) | [1-decanol] (M) | ethane signal |
| 0 | 0.100 | 0 | 0 | 0.100 | 0 |
| 10 | 0.096 | 535 | 10 | 0.083 | 0 |
| 20 | 0.091 | 1150 | 20 | 0.076 | 0 |
| 30 | 0.087 | 1754 | 30 | 0.073 | 0 |
| 45 | 0.079 | 2472 | 45 | 0.068 | 0 |
| 60 | 0.074 | 3261 | 60 | 0.064 | 0 |
| 75 | 0.070 | 3672 | 75 | 0.061 | 0 |
| 90 | 0.066 | 4216 | 90 | 0.059 | 0 |
| 120 | 0.061 | 4385 | 120 | 0.056 | 0 |
| 150 | 0.057 | 4830 | 150 | 0.053 | 0 |
| 180 | 0.055 | 4672 | 180 | 0.051 | 0 |
| 210 | 0.053 | 4793 | 210 | 0.051 | 0 |

**Numerical Data for the Hydrogenation of Hexyl Hexanoate with Headspace Monitoring**

**Table S3.** Numerical data for the hydrogenation of hexyl hexanoate by **Ru-de**, **Ru-imine**, and **Ru-de** with 10% 1-hexanol, as shown in graphical form in Figure 3.

| **Ru-de** | | | **Ru-imine** | | | **Ru-de** + 10% 1-hexanol | | |
|---|---|---|---|---|---|---|---|---|
| t (min) | [HH] (M) | ethane signal | t (min) | [HH] (M) | ethane signal | t (min) | [HH] | ethane signal |
| 0 | 0.400 | 0 | 0 | 0.400 | 0 | 0 | 0.400 | 0 |
| 10 | 0.397 | 60 | 10 | 0.070 | 0 | 10 | 0.302 | 87 |
| 20 | 0.393 | 120 | 20 | 0.014 | 0 | 20 | 0.188 | 262 |
| 30 | 0.365 | 189 | 30 | 0.005 | 0 | 30 | 0.103 | 300 |
| 45 | 0.205 | 349 | 45 | 0.004 | 0 | 45 | 0.035 | 349 |
| 60 | 0.087 | 391 | 60 | 0.003 | 0 | 60 | 0.015 | 415 |
| 75 | 0.033 | 465 | 75 | 0.004 | 0 | 75 | 0.006 | 431 |
| 90 | 0.011 | 526 | 90 | 0.003 | 0 | 90 | 0.004 | 457 |
| 120 | 0.015 | 588 | 120 | 0.005 | 0 | 120 | 0.003 | 487 |
| 150 | 0.002 | 656 | | | | 150 | 0.002 | 537 |
| 180 | 0.003 | 686 | | | | | | |
| 210 | 0.005 | 728 | | | | | | |
| 240 | 0.006 | 734 | | | | | | |

**Apparatus for Kinetic Experiments in Parallel**

The kinetic experiments shown in Figure 4 were conducted in an Asynt Multicell Parallel High-Pressure Reactor, designed to allow sampling of aliquots from five hydrogenation reactions run in parallel. Our customization of this apparatus was described previously,[1] but is repeated here for convenience. The reactor was customized to fit in our glovebox antechamber and to include reaction sampling valves on five reactor cells. The sampling valves employ 0.8 mm ID 1/16" stainless steel tubing, a Swagelok Low Flow metering valve (part no. SS-SS1) to control flow, and a Swagelok ball valve (part no. SS-41GS1) to allow removal of samples. As the internal volume of the sampling valve system was measured to be 0.25 mL, removal of 0.50 mL of liquid before collecting each aliquot ensures that a fresh sample is being taken directly from the reaction mixture. A sixth reactor cell is fitted with a thermocouple to allow control of the internal reaction temperatures. For reactions at 55 °C, the reactor was partially submerged (2 cm past the internal liquid level) in a stirred oil bath (a non-magnetic stainless-steel cooking pot), whose temperature is controlled using heating tape (Briskheat part no. BIH051040L) wrapped around the oil bath and an Omega Platinum Series Universal Benchtop Digital Controller, part no. CS8DPT. The oil bath is placed on a stirring hotplate, which allows stirring of the reactions and oil bath, as well as pre-heating of the oil to allow the reactions equilibrate to the desired temperature quickly.

**Numerical Data for Hydrogenation of Hexyl Hexanoate in the Asynt Parallel Reactor**

**Table S4.** Concentrations for the hydrogenation of hexyl hexanoate by **Ru-de**, shown in graphical form in Figure 4.

**Standard Conditions: [Ru-de] = 0.002 M, [ester]$_0$ = 0.40 M, P($H_2$) = 20 bar, [1-hexanol] = 0.16 M**

| t (s) | [HH] | [HA] | t (s) | [HH] | [HA] | t (s) | [HH] | [HA] |
|---|---|---|---|---|---|---|---|---|
| 0 | 0.4 | 0.16 | 0 | 0.4 | 0.16 | 0 | 0.4 | 0.16 |
| 600 | 0.401462 | 0.161036 | 780 | 0.398526 | 0.163211 | 600 | 0.387574 | 0.167155 |
| 1200 | 0.395183 | 0.173194 | 1380 | 0.394387 | 0.17269 | 1200 | 0.377357 | 0.183908 |
| 1800 | 0.389774 | 0.185215 | 1980 | 0.389689 | 0.18375 | 1800 | 0.367594 | 0.201085 |
| 2700 | 0.381277 | 0.203626 | 2880 | 0.381945 | 0.199113 | 2700 | 0.355055 | 0.227941 |
| 3600 | 0.36979 | 0.214307 | 3780 | 0.372945 | 0.21168 | 3600 | 0.341897 | 0.254314 |
| 4500 | 0.361816 | 0.239674 | 4680 | 0.364592 | 0.230685 | 4500 | 0.328631 | 0.276778 |
| 5400 | 0.352853 | 0.258019 | 5580 | 0.355233 | 0.252197 | 5400 | 0.314739 | 0.297336 |
| 7200 | 0.333585 | 0.295972 | 7380 | 0.338113 | 0.287422 | 7200 | 0.291046 | 0.349279 |
| 9000 | 0.315732 | 0.335608 | 9180 | 0.320295 | 0.316998 | 9000 | 0.267753 | 0.396652 |

**Variation of [1-hexanol] (appears in Figure 4, panel c):**

| 0.01 M HA | | | 0.02 M HA | | | 0.04 M HA | | | 0.08 M HA | | |
|---|---|---|---|---|---|---|---|---|---|---|---|
| t (s) | [HH] | [HA] | t (s) | [HH] | [HA] | t (s) | [HH] | [HA] | t (s) | [HH] | [HA] |
| 0 | 0.4 | 0.01 | 0 | 0.4 | 0.02 | 0 | 0.4 | 0.04 | 0 | 0.4 | 0.08 |
| 720 | 0.400538 | 0.009166 | 660 | 0.40024 | 0.020031 | 600 | 0.397862 | 0.044304 | 660 | 0.400111 | 0.080076 |
| 1320 | 0.401658 | 0.012406 | 1260 | 0.400823 | 0.024929 | 1200 | 0.398203 | 0.049673 | 1260 | 0.397009 | 0.092165 |
| 1920 | 0.40153 | 0.011954 | 1860 | 0.398928 | 0.028638 | 1800 | 0.395135 | 0.056831 | 1860 | 0.393328 | 0.100404 |
| 2820 | 0.400774 | 0.013086 | 2760 | 0.396245 | 0.033289 | 2700 | 0.38822 | 0.065234 | 2760 | 0.387129 | 0.111425 |
| 4620 | 0.399287 | 0.01609 | 3660 | 0.396226 | 0.027826 | 3600 | 0.383413 | 0.07844 | 3660 | 0.38003 | 0.125588 |
| 5520 | 0.398448 | 0.018709 | 4560 | 0.388578 | 0.047122 | 4500 | 0.376727 | 0.090386 | 4560 | 0.37296 | 0.137837 |
| 7320 | 0.395826 | 0.021857 | 5460 | 0.383573 | 0.05544 | 5400 | 0.369388 | 0.103431 | 5460 | 0.363921 | 0.151737 |
| 9120 | 0.393442 | 0.027529 | 7260 | 0.372566 | 0.07642 | 7200 | 0.354337 | 0.13016 | 7260 | 0.348003 | 0.181847 |
| | | | 9060 | 0.360165 | 0.102306 | 9000 | 0.33659 | 0.164043 | 9060 | 0.329327 | 0.221567 |

| 0.12 M HA | | | 0.12 M HA | | | 0.24 M HA | | |
|---|---|---|---|---|---|---|---|---|
| t (s) | [HH] | [HA] | t (s) | [HH] | [HA] | t (s) | [HH] | [HA] |
| 0 | 0.4 | 0.12 | 0 | 0.4 | 0.12 | 0 | 0.4 | 0.24 |
| 720 | 0.397148 | 0.125677 | 660 | 0.391102 | 0.125288 | 840 | 0.397461 | 0.245354 |
| 1320 | 0.395878 | 0.134246 | 1260 | 0.382074 | 0.143783 | 1440 | 0.392454 | 0.25784 |
| 1920 | 0.391327 | 0.140458 | 1860 | 0.373095 | 0.158679 | 2040 | 0.385631 | 0.271778 |
| 2820 | 0.38473 | 0.153383 | 2760 | 0.362848 | 0.179937 | 2940 | 0.376963 | 0.287289 |
| 3720 | 0.378054 | 0.165792 | 3660 | 0.353227 | 0.197432 | 3840 | 0.366654 | 0.309482 |
| 4620 | 0.371005 | 0.180411 | 4560 | 0.344015 | 0.216754 | 4740 | 0.356027 | 0.327381 |
| 5520 | 0.36229 | 0.198047 | 5460 | 0.335794 | 0.230395 | 5640 | 0.345754 | 0.350627 |
| 7320 | 0.348446 | 0.222012 | 7260 | 0.321259 | 0.256615 | 7440 | 0.325477 | 0.390114 |
| 9120 | 0.330379 | 0.258212 | 9060 | 0.307905 | 0.290084 | 9240 | 0.306457 | 0.429781 |

**Variation of [Ru-de] (appears in Figure 4, panel a):**

| 0.001 M Ru | | | 0.003 M Ru | | |
|---|---|---|---|---|---|
| t (s) | [HH] | [HA] | t (s) | [HH] | [HA] |
| 0 | 0.4 | 0.16 | 0 | 0.4 | 0.16 |
| 660 | 0.403481 | 0.157217 | 600 | 0.398729 | 0.16732 |
| 1260 | 0.401653 | 0.163094 | 1200 | 0.387335 | 0.188697 |
| 1860 | 0.399262 | 0.169481 | 1800 | 0.374574 | 0.210808 |
| 2760 | 0.39593 | 0.177364 | 2700 | 0.357192 | 0.250386 |
| 3660 | 0.392177 | 0.184185 | 3600 | 0.339355 | 0.282004 |
| 4560 | 0.386268 | 0.184212 | 4500 | 0.32211 | 0.317146 |
| 5460 | 0.383841 | 0.196568 | 5400 | 0.304138 | 0.350831 |
| 7260 | 0.376457 | 0.217805 | 7200 | 0.272052 | 0.41267 |
| 9060 | 0.36541 | 0.217621 | 9000 | 0.240067 | 0.475331 |

**Table S4.** continued

**Variation of [1-hexanol]$_0$ (Appears in Figure 4, panel b):**

| 0.05 M HH | | |
|---|---|---|
| t (s) | [HH] | [HA] |
| 0 | 0.05 | 0.16 |
| 720 | 0.049138 | 0.169126 |
| 1320 | 0.04612 | 0.178425 |
| 1920 | 0.043311 | 0.182555 |
| 2820 | 0.040191 | 0.191074 |
| 3720 | 0.03664 | 0.196603 |
| 4620 | 0.033743 | 0.20266 |
| 5520 | 0.030798 | 0.207156 |
| 7320 | 0.026383 | 0.218899 |
| 9120 | 0.02231 | 0.227447 |

| 0.10 M HH | | |
|---|---|---|
| t (s) | [HH] | [HA] |
| 0 | 0.1 | 0.16 |
| 840 | 0.102778 | 0.172427 |
| 1440 | 0.099886 | 0.181285 |
| 2040 | 0.095744 | 0.189054 |
| 2940 | 0.090799 | 0.197356 |
| 3840 | 0.08588 | 0.21003 |
| 4740 | 0.080433 | 0.218407 |
| 5640 | 0.076534 | 0.23269 |
| 7440 | 0.067528 | 0.245617 |
| 9240 | 0.059481 | 0.267034 |

| 0.20 M HH | | |
|---|---|---|
| t (s) | [HH] | [HA] |
| 0 | 0.2 | 0.16 |
| 780 | 0.205716 | 0.161956 |
| 1380 | 0.203246 | 0.181118 |
| 1980 | 0.198405 | 0.192182 |
| 2880 | 0.191135 | 0.203165 |
| 3780 | 0.183333 | 0.217721 |
| 4680 | 0.177919 | 0.234581 |
| 5580 | 0.169764 | 0.24813 |
| 7380 | 0.156713 | 0.274996 |
| 9180 | 0.143792 | 0.28924 |

| 0.20 M HH | | |
|---|---|---|
| t (s) | [HH] | [HA] |
| 0 | 0.2 | 0.16 |
| 780 | 0.201621 | 0.164874 |
| 1380 | 0.196765 | 0.17983 |
| 1980 | 0.190965 | 0.184989 |
| 2880 | 0.185089 | 0.201603 |
| 3780 | 0.176691 | 0.208986 |
| 4680 | 0.169058 | 0.222965 |
| 5580 | 0.165342 | 0.246428 |
| 7380 | 0.153177 | 0.268746 |
| 9180 | 0.13953 | 0.283524 |

| 0.60 M HH | | |
|---|---|---|
| t (s) | [HH] | [HA] |
| 0 | 0.6 | 0.16 |
| 840 | 0.59738 | 0.168664 |
| 1440 | 0.590144 | 0.178285 |
| 2040 | 0.582807 | 0.199091 |
| 2940 | 0.571414 | 0.218643 |
| 3840 | 0.559746 | 0.241261 |
| 4740 | 0.546571 | 0.267982 |
| 5640 | 0.533713 | 0.292583 |
| 7440 | 0.508637 | 0.344903 |
| 9240 | 0.482413 | 0.391598 |

**Variation of P($H_2$) (Appears in Figure 4, panel d):**

| 10 bar | | |
|---|---|---|
| t (s) | [HH] | [HA] |
| 0 | 0.4 | 0.16 |
| 600 | 0.400263 | 0.163118 |
| 1200 | 0.395391 | 0.170166 |
| 1800 | 0.390461 | 0.183352 |
| 2700 | 0.381099 | 0.19724 |
| 3600 | 0.374014 | 0.214189 |
| 4500 | 0.364122 | 0.232655 |
| 5400 | 0.355312 | 0.25022 |
| 7200 | 0.336365 | 0.289589 |
| 9000 | 0.315182 | 0.329938 |

| 10 bar | | |
|---|---|---|
| t (s) | [HH] | [HA] |
| 0 | 0.4 | 0.16 |
| 660 | 0.400012 | 0.164061 |
| 1260 | 0.393389 | 0.173808 |
| 1860 | 0.389908 | 0.184536 |
| 2760 | 0.380176 | 0.203807 |
| 3660 | 0.370322 | 0.222895 |
| 4560 | 0.359738 | 0.24657 |
| 5460 | 0.348668 | 0.266916 |
| 7260 | 0.326167 | 0.31174 |
| 9060 | 0.304443 | 0.355769 |

| 10 bar | | |
|---|---|---|
| t (s) | [HH] | [HA] |
| 0 | 0.4 | 0.16 |
| 600 | 0.398473 | 0.166443 |
| 1200 | 0.39013 | 0.179787 |
| 1800 | 0.382148 | 0.194214 |
| 2700 | 0.370405 | 0.218183 |
| 3600 | 0.358773 | 0.244102 |
| 4500 | 0.345871 | 0.265497 |
| 5400 | 0.333248 | 0.288206 |
| 7200 | 0.309795 | 0.33768 |
| 9000 | 0.286869 | 0.385015 |

| 10 bar | | |
|---|---|---|
| t (s) | [HH] | [HA] |
| 0 | 0.4 | 0.16 |
| 660 | 0.397383 | 0.168998 |
| 1260 | 0.390521 | 0.18046 |
| 1860 | 0.381952 | 0.19861 |
| 2760 | 0.370622 | 0.219656 |
| 3660 | 0.358786 | 0.243367 |
| 4560 | 0.346762 | 0.266713 |
| 5460 | 0.334174 | 0.290076 |
| 7260 | 0.310489 | 0.339571 |
| 9060 | 0.285508 | 0.388814 |

| 30 bar | | |
|---|---|---|
| t (s) | [HH] | [HA] |
| 0 | 0.4 | 0.16 |
| 600 | 0.396838 | 0.169656 |
| 1200 | 0.387582 | 0.184602 |
| 1800 | 0.378366 | 0.201201 |
| 2700 | 0.364695 | 0.225206 |
| 3600 | 0.352265 | 0.249312 |
| 4500 | 0.33976 | 0.27167 |
| 5400 | 0.327148 | 0.296636 |
| 7200 | 0.303915 | 0.346892 |
| 9480 | 0.273742 | 0.405444 |

| 30 bar | | |
|---|---|---|
| t (s) | [HH] | [HA] |
| 0 | 0.4 | 0.16 |
| 660 | 0.397148 | 0.169872 |
| 1260 | 0.389418 | 0.185926 |
| 1860 | 0.380516 | 0.201877 |
| 2760 | 0.36745 | 0.226953 |
| 3660 | 0.353879 | 0.25162 |
| 4560 | 0.340053 | 0.281271 |
| 5460 | 0.326995 | 0.304871 |
| 7260 | 0.302104 | 0.358049 |
| 9540 | 0.271788 | 0.413198 |

**Table S5.** Initial rates for the hydrogenation of hexyl hexanoate by **Ru-de**, shown in graphical form in Figure 4.

| Experiment | $[Ru\text{-}de]_0$ (M) | $[ester]_0$ (M) | $[1\text{-}hexanol]_0$ (M) | $P(H_2)$ (bar) | Initial rate x $10^5$ (M/s) | Appears in Figure 4 panels |
|---|---|---|---|---|---|---|
| 0 | 0.002 | 0.4 | 0.16 | 20 | 0.906 | (a,b,c,d), Standard conditions |
| 1 | 0.002 | 0.4 | 0.16 | 20 | 0.989 | (a,b,c,d), Standard conditions |
| 2 | 0.002 | 0.4 | 0.16 | 20 | 1.455 | (a,b,c,d), Standard conditions |
| 3 | 0.001 | 0.4 | 0.16 | 20 | 0.412 | a, Variation of $[Ru\text{-}de]_0$ |
| 4 | 0.003 | 0.4 | 0.16 | 20 | 1.194 | a, Variation of $[Ru\text{-}de]_0$ |
| 5 | 0.003 | 0.4 | 0.16 | 20 | 1.858 | a, Variation of $[Ru\text{-}de]_0$ |
| 6 | 0.002 | 0.05 | 0.16 | 20 | 0.320 | b, Variation of $[ester]_0$ |
| 7 | 0.002 | 0.1 | 0.16 | 20 | 0.491 | b, Variation of $[ester]_0$ |
| 8 | 0.002 | 0.2 | 0.16 | 20 | 0.702 | b, Variation of $[ester]_0$ |
| 9 | 0.002 | 0.2 | 0.16 | 20 | 0.695 | b, Variation of $[ester]_0$ |
| 10 | 0.002 | 0.6 | 0.16 | 20 | 1.321 | b, Variation of $[ester]_0$ |
| 11 | 0.002 | 0.4 | 0.01 | 20 | 0.080 | c, Variation of $[1\text{-}hexanol]_0$ |
| 12 | 0.002 | 0.4 | 0.02 | 20 | 0.449 | c, Variation of $[1\text{-}hexanol]_0$ |
| 13 | 0.002 | 0.4 | 0.04 | 20 | 0.709 | c, Variation of $[1\text{-}hexanol]_0$ |
| 14 | 0.002 | 0.4 | 0.08 | 20 | 0.803 | c, Variation of $[1\text{-}hexanol]_0$ |
| 15 | 0.002 | 0.4 | 0.12 | 20 | 0.775 | c, Variation of $[1\text{-}hexanol]_0$ |
| 16 | 0.002 | 0.4 | 0.12 | 20 | 1.018 | c, Variation of $[1\text{-}hexanol]_0$ |
| 17 | 0.002 | 0.4 | 0.24 | 20 | 1.062 | c, Variation of $[1\text{-}hexanol]_0$ |
| 18 | 0.002 | 0.4 | 0.16 | 10 | 0.969 | d, Variation of $P(H_2)$ |
| 19 | 0.002 | 0.4 | 0.16 | 10 | 1.098 | d, Variation of $P(H_2)$ |
| 20 | 0.002 | 0.4 | 0.16 | 10 | 1.305 | d, Variation of $P(H_2)$ |
| 21 | 0.002 | 0.4 | 0.16 | 10 | 1.301 | d, Variation of $P(H_2)$ |
| 22 | 0.002 | 0.4 | 0.16 | 30 | 1.369 | d, Variation of $P(H_2)$ |
| 23 | 0.002 | 0.4 | 0.16 | 30 | 1.401 | d, Variation of $P(H_2)$ |

**Determination of Overall Free-Energy Barrier for Ester Hydrogenation**

For comparison with the overall free-energy barrier calculated by DFT, we determined the experimental free-energy barrier based on the experimental kinetic data where the initial concentration of 1-hexanol was at least 0.080 M. This corresponds to all experiments in Table S5 above, except for experiments 11, 12, and 13. For each experiment, we calculated the rate constant k, assuming the following rate law:

rate = k[**Ru-de**][ester]

The average rate constant for these 21 experiments is 0.015 ± 0.005 $M^{-1}\cdot s^{-1}$

Using the Eyring equation with the reaction temperature of 328.15 K and a transmission coefficient $\kappa$ of 1, we find that the free energy barrier is 22.0 ± 0.2 kcal/mol.

**Numerical Data for the NMR study of catalyst speciation**

**Table S6.** NMR integrations and concentration ratios for the study of catalyst speciation shown in Figure 5.

| Experiment | **Ru-H** | $H_2$ | **Ru-alkoxide** | 1-hexanol | [**Ru-H**]/[**Ru-alkoxide**] | [$H_2$]/[1-hexanol] |
|---|---|---|---|---|---|---|
| 1 | 1.00 | 4.41 | 3.81 | 1234.67 | 0.13123 | 0.00536 |
| 2 | 1.00 | 1.66 | 1.95 | 222.62 | 0.25641 | 0.01118 |
| 3 | 1.00 | 2.35 | 2.14 | 386.11 | 0.23364 | 0.00913 |
| 4 | 1.00 | 1.90 | 1.14 | 177.88 | 0.43860 | 0.01602 |
| 5 | 1.00 | 2.56 | 2.08 | 398.04 | 0.24038 | 0.00965 |
| 6 | 1.00 | 3.43 | 2.61 | 733.66 | 0.19157 | 0.00701 |

**Energies calculated by DFT**

Table S7 below presents the solution-phase free energies calculated at the experimental reaction temperature of 328.15 K. For each species, the solution free energy was calculated as follows:

$$G^{\circ}_{total} = E_{single\text{-}point} + G_{solvation} + G_{correction} + 2.15 \text{ kcal/mol},$$

where $E_{single\text{-}point}$ is the electronic energy calculated in vacuum for the given functional, $G_{solvation}$ is the free energy of solvation calculated for the given solvent at 328.15 K using the COSMO-RS model as described above, and $G_{correction}$ is the free-energy correction resulting from a frequency calculation on the stationary point at the r$^2$SCAN-3c level. The addition of 2.15 kcal/mol to each species, to adjust the standard state from 1 atm to 1 M, ensures that $\Delta G^{\circ}$ values calculated for reactions with a change in molecularity appropriately use the 1 M standard state in solution. The $G^{\circ}_{rel}$ values reported in the table below are reported against the catalyst resting state **Ru-H**, the organic reactants ethyl acetate and one molecule of hydrogen, and two free ethanol molecules, which are included as explicit solvent for some species. Mass balance is ensured throughout; the column "Mass Balance" lists the small molecules added to each ruthenium species. The source data, including electronic energies, solvation free energies, the frequency correction, and the single imaginary frequency for transition states, is listed with each species in the included file "DFT structures.xyz".

**Table S7.** Free energies calculated by DFT. All values are standard-state free energies in kcal/mol. Bold values (ωB97M-V/toluene) represent the free energy values presented in the reaction coordinate diagram figures in the main text and Supporting Information.

| | | ωB97M-V | | | toluene | | | | | |
|---|---|---|---|---|---|---|---|---|---|---|
| **Organic Thermodynamics** | **Mass balance** | **toluene** | **THF** | **ethanol** | **M06L** | **MN15L** | **TPSS** | **TPSS_D4** | **M06** | **B3LYP-D3** |
| ethyl_acetate | 2 $H_2$ | **0.0** | 0.0 | 0.0 | 0.0 | 0.0 | 0.0 | 0.0 | 0.0 | 0.0 |
| acetaldehyde | EtOH + H2 | **5.4** | 3.7 | 3.3 | 4.9 | 10.5 | 6.7 | 8.7 | 5.8 | 4.8 |
| ethanol | EtOH | **-3.5** | -6.6 | -6.9 | -1.3 | 7.6 | 3.2 | 4.5 | -1.5 | -1.6 |
| **Potential Catalyst Resting States (Fig. 6)** | | | | | | | | | | |
| Ru-de | 2 EtOH + 2 $H_2$ + EtOAc | **8.0** | 8.1 | 8.4 | 6.1 | 6.3 | 2.8 | 4.6 | 2.8 | -2.2 |
| Ru-de-e | EtOH + 2 $H_2$ + EtOAc | **12.1** | 13.9 | 14.7 | 9.9 | 7.4 | 18.3 | 9.7 | 10.6 | 8.6 |
| Ru-de-2e | 2 $H_2$ + EtOAc | **9.5** | 13.2 | 14.3 | 10.2 | 7.4 | 21.8 | 6.4 | 9.6 | 5.3 |
| Ru-OEt | EtOH + 2 $H_2$ + EtOAc | **3.5** | 5.2 | 5.1 | 1.8 | 1.8 | 5.9 | -1.4 | 3.5 | 1.0 |
| Ru-OEt-e | 2 $H_2$ + EtOAc | **2.0** | 5.4 | 5.9 | 1.8 | -0.7 | 13.3 | -1.2 | 3.0 | -0.3 |
| Ru-OEt-2e | 2 $H_2$ + EtOAc - EtOH | **2.5** | 7.6 | 8.7 | 3.2 | -1.9 | 20.5 | 0.0 | 4.2 | 0.0 |
| Ru-H | 2 EtOH + $H_2$ + EtOAc | **0.0** | 0.0 | 0.0 | 0.0 | 0.0 | 0.0 | 0.0 | 0.0 | 0.0 |
| Ru-H-e | EtOH + $H_2$ + EtOAc | **2.0** | 3.7 | 3.9 | 2.5 | 0.7 | 8.6 | 1.8 | 3.2 | 1.4 |
| Ru-H-2e | $H_2$ + EtOAc | **4.3** | 7.6 | 8.3 | 5.2 | 0.9 | 17.1 | 4.3 | 6.1 | 3.2 |
| **Hydrogen Activation MEP (Fig. 7)** | | | | | | | | | | |
| Ru-OEt-e | 2 $H_2$ + EtOAc | **2.0** | 5.4 | 5.9 | 1.8 | -0.7 | 13.3 | -1.2 | 3.0 | -0.3 |
| TSa | 2 $H_2$ + EtOAc | **21.4** | 24.7 | 23.9 | 20.9 | 18.8 | 29.9 | 17.4 | 19.9 | 10.7 |
| b | 2 $H_2$ + EtOAc | **20.3** | 23.6 | 22.9 | 19.8 | 17.4 | 28.3 | 16.2 | 18.7 | 9.5 |
| c | $H_2$ + EtOAc | **19.2** | 22.4 | 22.3 | 20.1 | 22.7 | 28.8 | 18.2 | 20.3 | 15.5 |
| TSd | $H_2$ + EtOAc | **20.1** | 23.4 | 23.4 | 21.6 | 24.0 | 30.0 | 19.4 | 21.6 | 16.6 |
| Ru-H | 2 EtOH + $H_2$ + EtOAc | **0.0** | 0.0 | 0.0 | 0.0 | 0.0 | 0.0 | 0.0 | 0.0 | 0.0 |

| | | ωB97M-V | | | toluene | | | | | |
|---|---|---|---|---|---|---|---|---|---|---|
| | | toluene | THF | ethanol | M06L | MN15L | TPSS | TPSS_D4 | M06 | B3LYP-D3 |
| **Ester Hydrogenolysis MEP With No Explicit EtOH (Dechelation, Fig. 8)** | | | | | | | | | | |
| Ru-H | 2 EtOH + $H_2$ + EtOAc | **0.0** | 0.0 | 0.0 | 0.0 | 0.0 | 0.0 | 0.0 | 0.0 | 0.0 |
| TSe | 2 EtOH + $H_2$ + EtOAc | **26.2** | 26.2 | 25.9 | 22.4 | 24.8 | 25.2 | 27.5 | 23.4 | 20.2 |
| f | 2 EtOH + $H_2$ + EtOAc | **19.8** | 19.8 | 19.6 | 16.5 | 19.4 | 15.1 | 20.0 | 16.3 | 13.5 |
| g | 2 EtOH + $H_2$ | **21.7** | 21.9 | 22.3 | 19.4 | 20.4 | 30.2 | 24.3 | 21.7 | 21.7 |
| TSh-S | 2 EtOH + $H_2$ | **26.5** | 26.8 | 26.8 | 27.6 | 25.3 | 35.6 | 29.3 | 28.3 | 28.2 |
| i-S | 2 EtOH + $H_2$ | **22.8** | 23.1 | 21.9 | 22.6 | 22.4 | 32.0 | 24.0 | 22.5 | 22.6 |
| TSj-S | 2 EtOH + $H_2$ | **26.5** | 26.8 | 25.7 | 26.6 | 27.8 | 36.0 | 28.6 | 26.0 | 21.3 |
| k-S | 2 EtOH + $H_2$ | **21.7** | 21.9 | 22.2 | 21.2 | 25.1 | 26.3 | 22.3 | 20.3 | 15.4 |
| TSl-S | 2 EtOH + $H_2$ | **25.3** | 25.6 | 25.3 | 23.2 | 24.2 | 35.0 | 26.9 | 24.3 | 19.7 |
| m-S | 2 EtOH + $H_2$ | **19.0** | 19.4 | 18.8 | 19.2 | 21.7 | 29.9 | 20.8 | 21.0 | 19.3 |
| TSn-S | 2 EtOH + $H_2$ | **25.0** | 25.3 | 26.1 | 20.1 | 27.5 | 32.3 | 24.2 | 24.2 | 21.2 |
| o | 2 EtOH + $H_2$ + MeCHO | **20.2** | 20.2 | 19.5 | 13.8 | 22.1 | 16.5 | 18.5 | 15.4 | 10.4 |
| TSp | 2 EtOH + $H_2$ + MeCHO | **25.3** | 25.3 | 24.6 | 16.7 | 22.7 | 25.2 | 22.7 | 20.2 | 14.6 |
| Ru-OEt-e | EtOH + $H_2$ + MeCHO | **7.4** | 9.1 | 9.2 | 6.6 | 9.9 | 20.0 | 7.4 | 8.8 | 4.5 |
| **Addition of Explicit EtOH to Key Species in the Above Pathway** | | | | | | | | | | |
| TSh-e-S | EtOH + $H_2$ | **28.2** | 30.1 | 30.6 | 29.8 | 23.7 | 44.4 | 31.8 | 30.6 | 29.9 |
| TSj-e-S | EtOH + $H_2$ | **25.9** | 27.7 | 27.9 | 26.5 | 24.9 | 41.8 | 28.8 | 25.2 | 20.0 |
| **Ester Hydrogenolysis Alternative 1 With No Explicit EtOH (Hasanayn CH/OR Metathesis, Fig. 9)** | | | | | | | | | | |
| Ru-H | 2 EtOH + $H_2$ + EtOAc | **0.0** | 0.0 | 0.0 | 0.0 | 0.0 | 0.0 | 0.0 | 0.0 | 0.0 |
| TSq-R | 2 EtOH + $H_2$ | **29.8** | 30.1 | 29.3 | 28.5 | 27.0 | 37.5 | 24.2 | 28.4 | 25.0 |
| r-R | 2 EtOH + $H_2$ | **27.1** | 27.5 | 24.9 | 25.8 | 26.0 | 37.6 | 24.1 | 25.0 | 21.6 |
| TSs-R | 2 EtOH + $H_2$ | **32.7** | 33.2 | 27.7 | 31.3 | 32.3 | 44.1 | 31.9 | 32.1 | 25.1 |
| Ru-OEt-e | 2 EtOH + $H_2$ | **7.4** | 9.1 | 9.2 | 6.6 | 9.9 | 20.0 | 7.4 | 8.8 | 4.5 |
| **Addition of Explicit EtOH to Key Species in the Above Pathway (Note that the slip and C-O cleavage steps are now separate)** | | | | | | | | | | |
| TSq-e-R | EtOH + $H_2$ | **23.8** | 25.6 | 26.0 | 24.5 | 21.3 | 36.9 | 19.9 | 23.8 | 20.0 |
| r-e-R | EtOH + $H_2$ | **22.9** | 24.7 | 23.7 | 22.8 | 20.9 | 38.2 | 20.5 | 21.7 | 19.1 |
| TSs-e-R | EtOH + $H_2$ | **27.5** | 29.5 | 28.2 | 29.1 | 28.1 | 45.3 | 28.7 | 28.5 | 20.3 |
| t-e-R | EtOH + $H_2$ | **23.7** | 25.7 | 24.3 | 25.9 | 25.2 | 45.0 | 25.3 | 26.4 | 20.1 |
| TSu-e-R | EtOH + $H_2$ | **26.5** | 28.5 | 28.8 | 25.3 | 26.4 | 45.0 | 25.2 | 29.2 | 23.5 |
| **Ester Hydrogenolysis Alternative 2 With No Explicit EtOH (Dearomatization, Fig. 10)** | | | | | | | | | | |
| Ru-H | 2 EtOH + $H_2$ + EtOAc | **0.0** | 0.0 | 0.0 | 0.0 | 0.0 | 0.0 | 0.0 | 0.0 | 0.0 |
| TSq-R | 2 EtOH + $H_2$ | **29.8** | 30.1 | 29.3 | 28.5 | 27.0 | 37.5 | 24.2 | 28.4 | 25.0 |
| r-R | 2 EtOH + $H_2$ | **27.1** | 27.5 | 24.9 | 25.8 | 26.0 | 37.6 | 24.1 | 25.0 | 21.6 |
| TSv-R | 2 EtOH + $H_2$ | **33.4** | 33.4 | 33.4 | 33.6 | 33.8 | 44.6 | 34.0 | 32.5 | 25.8 |
| w-R | 2 EtOH + $H_2$ | **8.4** | 8.4 | 8.4 | 11.1 | 10.7 | 22.4 | 10.6 | 12.0 | 9.6 |
| TSx-R | 2 EtOH + $H_2$ | **28.5** | 28.9 | 29.5 | 27.1 | 30.3 | 37.6 | 23.3 | 29.4 | 23.6 |
| Ru-de | 3 EtOH + $H_2$ + MeCHO | **13.4** | 11.8 | 11.8 | 11.0 | 16.8 | 9.5 | 13.3 | 8.5 | 2.6 |
| TSy | 2 EtOH + $H_2$ + MeCHO | **25.3** | 25.4 | 25.7 | 24.2 | 29.3 | 29.0 | 22.8 | 26.4 | 22.0 |
| Ru-OEt-e | 2 EtOH + $H_2$ | **7.4** | 9.1 | 9.2 | 6.6 | 9.9 | 20.0 | 7.4 | 8.8 | 4.5 |
| **Addition of Explicit EtOH to Key Species in the Above Pathway** | | | | | | | | | | |
| TSq-e-R | EtOH + $H_2$ | **23.8** | 25.6 | 26.0 | 24.5 | 21.3 | 36.9 | 19.9 | 23.8 | 20.0 |
| r-e-R | EtOH + $H_2$ | **22.9** | 24.7 | 23.7 | 22.8 | 20.9 | 38.2 | 20.5 | 21.7 | 19.1 |
| TSv-e-R | EtOH + $H_2$ | **26.3** | 28.2 | 28.5 | 29.8 | 27.6 | 46.4 | 29.7 | 27.3 | 19.6 |
| TSx-e-R | EtOH + $H_2$ | **28.0** | 30.0 | 30.6 | 27.3 | 28.8 | 44.3 | 25.4 | 29.8 | 22.6 |
| TSy-e | EtOH + $H_2$ + MeCHO | **21.0** | 22.8 | 23.2 | 21.2 | 27.2 | 31.4 | 17.8 | 22.7 | 15.3 |
| **Aldehyde Reduction MEP, Fig. 11** | | | | | | | | | | |
| Ru-H | 3 EtOH + MeCHO | **5.4** | 3.7 | 3.3 | 4.9 | 10.5 | 6.7 | 8.7 | 5.8 | 4.8 |
| TSz-e | 2 EtOH | **17.6** | 17.8 | 18.1 | 17.2 | 19.8 | 28.9 | 18.6 | 19.1 | 14.9 |
| aa-e | 2 EtOH | **15.7** | 15.9 | 13.3 | 13.7 | 19.9 | 27.3 | 15.4 | 15.3 | 10.9 |
| TSab-e | 2 EtOH | **19.4** | 19.5 | 17.3 | 20.3 | 27.0 | 34.3 | 24.1 | 20.3 | 11.3 |
| Ru-OEt-e | 2 EtOH | **-1.5** | -1.2 | -1.0 | 0.4 | 6.9 | 16.6 | 3.3 | 1.5 | -1.9 |

| Hydrogen Activation Alternative Pathways | | ωB97M-V | | | toluene | | | | | |
|---|---|---|---|---|---|---|---|---|---|---|
| | | toluene | THF | ethanol | M06L | MN15L | TPSS | TPSS_D4 | M06 | B3LYP-D3 |
| **Deprotonation of $H_2$ by Ethoxide - No Explicit EtOH, Fig. S1** | | | | | | | | | | |
| Ru-OEt-e | 2 $H_2$ + EtOAc | **2.0** | 5.4 | 5.9 | 1.8 | -0.7 | 13.3 | -1.2 | 3.0 | -0.3 |
| b-e | EtOH + 2 $H_2$ + EtOAc | **34.6** | 36.2 | 30.0 | 30.5 | 31.9 | 29.2 | 27.2 | 30.5 | 23.5 |
| c-e | EtOH + $H_2$ + EtOAc | **35.1** | 36.8 | 28.4 | 32.3 | 35.9 | 33.7 | 31.0 | 33.5 | 30.9 |
| Ru-H | 2 EtOH + $H_2$ + EtOAc | **0.0** | 0.0 | 0.0 | 0.0 | 0.0 | 0.0 | 0.0 | 0.0 | 0.0 |
| **Dearomatized Pathway Involving the $PCH_2$ Linker, Fig. S2** | | | | | | | | | | |
| Ru-OEt-e | 2 $H_2$ + EtOAc | **2.0** | 5.4 | 5.9 | 1.8 | -0.7 | 13.3 | -1.2 | 3.0 | -0.3 |
| TSy | EtOH + 2 $H_2$ + EtOAc | **19.9** | 21.7 | 22.4 | 19.3 | 18.8 | 22.3 | 14.1 | 20.7 | 17.2 |
| Ru-de-e | EtOH + 2 $H_2$ + EtOAc | **12.1** | 13.9 | 14.7 | 9.9 | 7.4 | 18.3 | 9.7 | 10.6 | 8.6 |
| Ru-de | 2 EtOH + 2 $H_2$ + EtOAc | **8.0** | 8.1 | 8.4 | 6.1 | 6.3 | 2.8 | 4.6 | 2.8 | -2.2 |
| ac | 2 EtOH + $H_2$ + EtOAc | **10.5** | 10.6 | 10.9 | 8.8 | 12.2 | 7.2 | 9.7 | 7.0 | 7.3 |
| TSad | 2 EtOH + $H_2$ + EtOAc | **29.9** | 30.1 | 30.3 | 26.7 | 28.3 | 25.2 | 22.2 | 26.8 | 25.7 |
| Ru-H | 2 EtOH + $H_2$ + EtOAc | **0.0** | 0.0 | 0.0 | 0.0 | 0.0 | 0.0 | 0.0 | 0.0 | 0.0 |
| **Addition of Explicit EtOH to Key Species in the Above Pathway** | | | | | | | | | | |
| TSy-e | 2 $H_2$ + EtOAc | **15.6** | 19.1 | 19.9 | 16.3 | 16.6 | 24.7 | 9.1 | 17.0 | 10.5 |
| Ru-de-2e | 2 $H_2$ + EtOAc | **9.5** | 13.2 | 14.3 | 10.2 | 7.4 | 21.8 | 6.4 | 9.6 | 5.3 |
| TSad-e | EtOH + $H_2$ + EtOAc | **29.1** | 30.9 | 28.8 | 26.2 | 29.5 | 31.9 | 22.1 | 27.9 | 24.2 |
| **Dearomatized Pathway Involving the $NCH_2$ Linker, Fig. S3** | | | | | | | | | | |
| ac-N | 2 EtOH + $H_2$ + EtOAc | **22.4** | 22.6 | 22.9 | 20.6 | 23.1 | 19.4 | 18.7 | 18.4 | 18.8 |
| TSad-N | 2 EtOH + $H_2$ + EtOAc | **27.9** | 28.0 | 28.4 | 23.2 | 26.0 | 22.3 | 19.9 | 23.5 | 22.8 |
| Ru-H | 2 EtOH + $H_2$ + EtOAc | **0.0** | 0.0 | 0.0 | 0.0 | 0.0 | 0.0 | 0.0 | 0.0 | 0.0 |
| **Addition of Explicit EtOH to Key Species in the Above Pathway** | | | | | | | | | | |
| TSad-e-N | EtOH + $H_2$ + EtOAc | **31.3** | 33.2 | 30.8 | 28.7 | 31.8 | 32.1 | 26.4 | 29.8 | 26.2 |
| Ester Hydrogenolysis Alternative Pathways - Dechelation | | | | | | | | | | |
| **Hydride Insertion, Different Ru Configuration (O trans to Hydride), Fig. S4** | | | | | | | | | | |
| Ru-H | 2 EtOH + $H_2$ + EtOAc | **0.0** | 0.0 | 0.0 | 0.0 | 0.0 | 0.0 | 0.0 | 0.0 | 0.0 |
| TSae | 2 EtOH + $H_2$ + EtOAc | **24.0** | 24.1 | 24.0 | 18.1 | 21.9 | 21.7 | 24.2 | 17.0 | 16.1 |
| af | 2 EtOH + $H_2$ + EtOAc | **19.7** | 19.8 | 19.5 | 16.6 | 22.7 | 15.6 | 22.0 | 13.3 | 12.9 |
| ag-S | 2 EtOH + $H_2$ | **17.0** | 17.3 | 17.6 | 15.5 | 15.5 | 28.2 | 21.4 | 15.7 | 13.5 |
| TSah-S | 2 EtOH + $H_2$ | **26.2** | 26.5 | 27.0 | 26.9 | 25.9 | 34.9 | 29.6 | 26.8 | 26.4 |
| ai-S | 2 EtOH + $H_2$ | **27.4** | 27.6 | 27.8 | 26.1 | 26.5 | 35.8 | 27.5 | 26.9 | 26.7 |
| TSaj-S | 2 EtOH + $H_2$ | **28.3** | 28.5 | 28.6 | 27.9 | 29.5 | 37.5 | 29.6 | 27.5 | 22.0 |
| k-S | 2 EtOH + $H_2$ | **21.7** | 21.9 | 22.2 | 21.2 | 25.1 | 26.3 | 22.3 | 20.3 | 15.4 |
| **Hydride Insertion, Different Carbon Configuration (R-hemiacetaloxide), Fig S5** | | | | | | | | | | |
| Ru-H | 2 EtOH + $H_2$ + EtOAc | **0.0** | 0.0 | 0.0 | 0.0 | 0.0 | 0.0 | 0.0 | 0.0 | 0.0 |
| TSe | 2 EtOH + $H_2$ + EtOAc | **26.2** | 26.2 | 25.9 | 22.4 | 24.8 | 25.2 | 27.5 | 23.4 | 20.2 |
| f | 2 EtOH + $H_2$ + EtOAc | **19.8** | 19.8 | 19.6 | 16.5 | 19.4 | 15.1 | 20.0 | 16.3 | 13.5 |
| g | 2 EtOH + $H_2$ | **21.7** | 21.9 | 22.3 | 19.4 | 20.4 | 30.2 | 24.3 | 21.7 | 21.7 |
| TSh-R | 2 EtOH + $H_2$ | **26.6** | 26.9 | 27.4 | 27.8 | 28.2 | 33.4 | 29.7 | 28.4 | 27.4 |
| i-R | 2 EtOH + $H_2$ | **21.7** | 22.0 | 21.2 | 21.5 | 23.6 | 28.7 | 22.9 | 21.6 | 21.4 |
| TSj-R | 2 EtOH + $H_2$ | **27.0** | 27.2 | 26.3 | 26.9 | 29.5 | 33.2 | 28.7 | 25.7 | 20.9 |
| k-R | 2 EtOH + $H_2$ | **20.8** | 21.0 | 20.9 | 20.2 | 23.9 | 25.8 | 21.9 | 19.3 | 14.7 |
| **Addition of Explicit EtOH to Key Species in the Above Pathway** | | | | | | | | | | |
| TSh-e-R | EtOH + $H_2$ | **28.7** | 30.7 | 31.3 | 30.0 | 25.7 | 44.4 | 32.9 | 31.1 | 29.4 |

| | | ωB97M-V | | | toluene | | | | | |
|---|---|---|---|---|---|---|---|---|---|---|
| | | toluene | THF | ethanol | M06L | MN15L | TPSS | TPSS_D4 | M06 | B3LYP-D3 |
| **Hydride Insertion, Different Ru Configuration (O trans to Hydride) and Carbon Configuration (R-hemiacetaloxide), Fig. S6** | | | | | | | | | | |
| Ru-H | 2 EtOH + $H_2$ + EtOAc | **0.0** | 0.0 | 0.0 | 0.0 | 0.0 | 0.0 | 0.0 | 0.0 | 0.0 |
| TSae | 2 EtOH + $H_2$ + EtOAc | **24.0** | 24.1 | 24.0 | 18.1 | 21.9 | 21.7 | 24.2 | 17.0 | 16.1 |
| af | 2 EtOH + $H_2$ + EtOAc | **19.7** | 19.8 | 19.5 | 16.6 | 22.7 | 15.6 | 22.0 | 13.3 | 12.9 |
| ag-R | 2 EtOH + $H_2$ | **14.1** | 14.4 | 14.5 | 12.1 | 12.7 | 23.7 | 17.6 | 12.9 | 13.6 |
| TSah-R | 2 EtOH + $H_2$ | **23.8** | 24.1 | 24.6 | 25.3 | 23.2 | 33.5 | 28.2 | 24.4 | 24.5 |
| ai-R | 2 EtOH + $H_2$ | **23.7** | 23.9 | 24.1 | 23.8 | 23.1 | 33.1 | 28.0 | 23.6 | 23.8 |
| TSaj-S | 2 EtOH + $H_2$ | **27.1** | 27.3 | 27.3 | 27.1 | 27.9 | 35.9 | 28.5 | 26.7 | 21.2 |
| k-R | 2 EtOH + $H_2$ | **20.8** | 21.0 | 20.9 | 20.2 | 23.9 | 25.8 | 21.9 | 19.3 | 14.7 |
| **Addition of Explicit EtOH to Key Species in the Above Pathway** | | | | | | | | | | |
| TSah-e-R | EtOH + $H_2$ | **26.1** | 28.2 | 29.0 | 28.7 | 25.9 | 42.5 | 31.5 | 27.6 | 26.9 |
| TSaj-e-S | 2 EtOH + $H_2$ | **26.6** | 28.5 | 29.4 | 27.6 | 27.4 | 43.2 | 29.5 | 26.9 | 20.9 |
| **C-O Cleavage, Different Ru Configuration (OEt trans to Hydride), Fig. S7** | | | | | | | | | | |
| k-S | 2 EtOH + $H_2$ | **21.7** | 21.9 | 22.2 | 21.2 | 25.1 | 26.3 | 22.3 | 20.3 | 15.4 |
| ak-S | 2 EtOH + $H_2$ | **19.4** | 19.7 | 19.0 | 19.6 | 23.2 | 28.4 | 21.3 | 21.0 | 19.4 |
| TSal-S | 2 EtOH + $H_2$ | **29.1** | 29.4 | 29.2 | 24.2 | 31.0 | 35.5 | 28.1 | 29.4 | 26.0 |
| o | 2 EtOH + $H_2$ + MeCHO | **20.2** | 20.2 | 19.5 | 13.8 | 22.1 | 16.5 | 18.5 | 15.4 | 10.4 |
| TSp | 2 EtOH + $H_2$ + MeCHO | **25.3** | 25.3 | 24.6 | 16.7 | 22.7 | 25.2 | 22.7 | 20.2 | 14.6 |
| Ru-OEt-e | EtOH + $H_2$ + MeCHO | **7.4** | 9.1 | 9.2 | 6.6 | 9.9 | 20.0 | 7.4 | 8.8 | 4.5 |
| **C-O Cleavage, Different Carbon Configuration (R-hemiacetaloxide), Fig. S8** | | | | | | | | | | |
| k-R | 2 EtOH + $H_2$ | **20.8** | 21.0 | 20.9 | 20.2 | 23.9 | 25.8 | 21.9 | 19.3 | 14.7 |
| TSam-R | 2 EtOH + $H_2$ | **22.0** | 22.2 | 21.8 | 20.7 | 22.7 | 28.2 | 22.4 | 21.0 | 16.0 |
| m-R | 2 EtOH + $H_2$ | **15.5** | 15.9 | 15.5 | 15.2 | 17.1 | 26.4 | 17.1 | 17.2 | 15.6 |
| TSn-R | 2 EtOH + $H_2$ | **23.9** | 24.1 | 24.8 | 19.7 | 26.7 | 32.0 | 24.6 | 23.4 | 18.3 |
| o | 2 EtOH + $H_2$ + MeCHO | **20.2** | 20.2 | 19.5 | 13.8 | 22.1 | 16.5 | 18.5 | 15.4 | 10.4 |
| TSp | 2 EtOH + $H_2$ + MeCHO | **25.3** | 25.3 | 24.6 | 16.7 | 22.7 | 25.2 | 22.7 | 20.2 | 14.6 |
| Ru-OEt-e | EtOH + $H_2$ + MeCHO | **7.4** | 9.1 | 9.2 | 6.6 | 9.9 | 20.0 | 7.4 | 8.8 | 4.5 |
| **C-O Cleavage, Different Ru Configuration (OEt trans to Hydride) and Different Carbon Configuration (R-hemiacetaloxide), Fig S9** | | | | | | | | | | |
| k-R | 2 EtOH + $H_2$ | **20.8** | 21.0 | 20.9 | 20.2 | 23.9 | 25.8 | 21.9 | 19.3 | 14.7 |
| ak-R | 2 EtOH + $H_2$ | **18.1** | 18.3 | 17.2 | 18.4 | 20.6 | 29.1 | 20.3 | 19.6 | 18.0 |
| TSal-R | 2 EtOH + $H_2$ | **33.0** | 33.3 | 33.3 | 27.4 | 34.0 | 40.8 | 32.3 | 32.9 | 29.9 |
| o | 2 EtOH + $H_2$ + MeCHO | **20.2** | 20.2 | 19.5 | 13.8 | 22.1 | 16.5 | 18.5 | 15.4 | 10.4 |
| TSp | 2 EtOH + $H_2$ + MeCHO | **25.3** | 25.3 | 24.6 | 16.7 | 22.7 | 25.2 | 22.7 | 20.2 | 14.6 |
| Ru-OEt-e | EtOH + $H_2$ + MeCHO | **7.4** | 9.1 | 9.2 | 6.6 | 9.9 | 20.0 | 7.4 | 8.8 | 4.5 |
| **C-O Cleavage Concerted with Aldehyde Dissociation, Fig. S10** | | | | | | | | | | |
| m-S | 2 EtOH + $H_2$ | **19.0** | 19.4 | 18.8 | 19.2 | 21.7 | 29.9 | 20.8 | 21.0 | 19.3 |
| TSan-S | 2 EtOH + $H_2$ | **26.7** | 27.1 | 27.8 | 21.2 | 25.2 | 35.8 | 27.5 | 24.7 | 17.7 |
| o | 2 EtOH + $H_2$ + MeCHO | **20.2** | 20.2 | 19.5 | 13.8 | 22.1 | 16.5 | 18.5 | 15.4 | 10.4 |
| **Addition of Explicit EtOH to Key Species in the Above Pathway** | | | | | | | | | | |
| TSan-e-S | EtOH + $H_2$ | **27.3** | 29.4 | 30.1 | 22.3 | 21.4 | 44.3 | 29.1 | 25.5 | 17.8 |
| **C-O Cleavage Concerted with Aldehyde Dissociation, Different Carbon Configuration (R-hemiacetaloxide), Fig. S11** | | | | | | | | | | |
| m-R | 2 EtOH + $H_2$ | **15.5** | 15.9 | 15.5 | 15.2 | 17.1 | 26.4 | 17.1 | 17.2 | 15.6 |
| TSan-R | 2 EtOH + $H_2$ | **28.0** | 28.3 | 29.1 | 22.7 | 28.1 | 35.6 | 28.2 | 25.9 | 18.5 |
| o | 2 EtOH + $H_2$ + MeCHO | **20.2** | 20.2 | 19.5 | 13.8 | 22.1 | 16.5 | 18.5 | 15.4 | 10.4 |
| **Addition of Explicit EtOH to Key Species in the Above Pathway** | | | | | | | | | | |
| TSan-e-R | EtOH + $H_2$ | **27.0** | 29.0 | 29.9 | 22.6 | 23.3 | 42.3 | 28.6 | 25.3 | 17.5 |

| Ester Hydrogenolysis Alternative Pathways - Hasanayn CH/OR Metathesis | | ωB97M-V | | | toluene | | | | | |
|---|---|---|---|---|---|---|---|---|---|---|
| | | toluene | THF | ethanol | M06L | MN15L | TPSS | TPSS_D4 | M06 | B3LYP-D3 |
| **Ester Hydrogenolysis Alternative 1 (Hasanayn), Addition of second explicit EtOH molecule, Fig, S12** | | | | | | | | | | |
| Ru-H | 2 EtOH + $H_2$ + EtOAc | **0.0** | 0.0 | 0.0 | 0.0 | 0.0 | 0.0 | 0.0 | 0.0 | 0.0 |
| TSq-2e-R | $H_2$ | **26.1** | 29.7 | 30.8 | 27.9 | 24.2 | 44.5 | 22.5 | 28.1 | 22.5 |
| r-2e-R | EtOH + $H_2$ | **24.0** | 27.5 | 27.5 | 26.7 | 22.8 | 49.8 | 26.5 | 24.3 | 16.9 |
| TSs-2r-R | $H_2$ | **27.0** | 30.5 | 30.0 | 29.6 | 26.1 | 52.2 | 29.6 | 28.3 | 20.0 |
| t-2e-R | $H_2$ | **19.1** | 22.8 | 22.5 | 22.6 | 19.7 | 46.6 | 21.5 | 23.1 | 18.3 |
| TSu-2e-R | $H_2$ | **29.2** | 32.9 | 33.5 | 27.9 | 26.7 | 52.1 | 28.3 | 31.9 | 25.7 |
| Ru-OEt-e | EtOH + $H_2$ + MeCHO | **7.4** | 9.1 | 9.2 | 6.6 | 9.9 | 20.0 | 7.4 | 8.8 | 4.5 |
| **Ester Hydrogenolysis Alternative 1 (Hasanayn), Different Carbon Configuration (R Hemiacetaloxide), Fig. S13** | | | | | | | | | | |
| Ru-H | 2 EtOH + $H_2$ + EtOAc | **0.0** | 0.0 | 0.0 | 0.0 | 0.0 | 0.0 | 0.0 | 0.0 | 0.0 |
| TSq-S | 2 EtOH + $H_2$ | **28.1** | 28.4 | 27.7 | 26.9 | 25.8 | 36.3 | 22.6 | 26.6 | 23.5 |
| r-S | 2 EtOH + $H_2$ | **26.4** | 26.8 | 24.0 | 25.7 | 26.5 | 35.9 | 23.3 | 24.8 | 22.5 |
| TSs-S | 2 EtOH + $H_2$ | **32.7** | 33.2 | 28.2 | 31.4 | 31.4 | 45.8 | 31.9 | 32.2 | 25.3 |
| Ru-OEt-e | EtOH + $H_2$ + MeCHO | **7.4** | 9.1 | 9.2 | 6.6 | 9.9 | 20.0 | 7.4 | 8.8 | 4.5 |
| **Addition of Explicit EtOH to Key Species in the Above Pathway (Note that the slip and C-O cleavage steps are now separate)** | | | | | | | | | | |
| TSq-e-S | EtOH + $H_2$ | **26.0** | 27.9 | 28.2 | 25.7 | 22.3 | 39.5 | 22.5 | 26.1 | 22.7 |
| r-e-S | EtOH + $H_2$ | **23.3** | 25.2 | 24.9 | 24.6 | 24.9 | 39.1 | 22.1 | 23.1 | 19.9 |
| TSs-e-S | EtOH + $H_2$ | **29.0** | 31.0 | 29.0 | 29.9 | 27.6 | 48.8 | 30.1 | 29.4 | 21.6 |
| t-e-S | EtOH + $H_2$ | **23.3** | 25.2 | 23.6 | 25.6 | 28.1 | 42.7 | 23.3 | 25.9 | 20.9 |
| TSu-e-S | EtOH + $H_2$ | **24.2** | 26.1 | 26.1 | 21.6 | 22.6 | 40.6 | 23.1 | 24.2 | 20.0 |
| Ester Hydrogenolysis Alternative Pathways - Dearomatization | | | | | | | | | | |
| **Ester Hydrogenolysis Alternative 2 (Dearomatization), Different Carbon Configuration (S Hemiacetaloxide), Fig. S14** | | | | | | | | | | |
| Ru-H | 2 EtOH + $H_2$ + EtOAc | **0.0** | 0.0 | 0.0 | 0.0 | 0.0 | 0.0 | 0.0 | 0.0 | 0.0 |
| TSq-S | 2 EtOH + $H_2$ | **28.1** | 28.4 | 27.7 | 26.9 | 25.8 | 36.3 | 22.6 | 26.6 | 23.5 |
| r-S | 2 EtOH + $H_2$ | **26.4** | 26.8 | 24.0 | 25.7 | 26.5 | 35.9 | 23.3 | 24.8 | 22.5 |
| TSv-S | 2 EtOH + $H_2$ | **30.2** | 30.3 | 23.9 | 32.9 | 31.5 | 40.0 | 32.4 | 28.8 | 23.0 |
| w-S | 2 EtOH + $H_2$ | **10.0** | 10.3 | 10.3 | 12.1 | 11.5 | 25.0 | 12.3 | 13.6 | 11.0 |
| TSx-S | 2 EtOH + $H_2$ | **32.9** | 33.3 | 33.7 | 29.9 | 33.6 | 42.8 | 28.3 | 33.2 | 27.1 |
| Ru-de | 3 EtOH + $H_2$ + MeCHO | **13.4** | 11.8 | 11.8 | 11.0 | 16.8 | 9.5 | 13.3 | 8.5 | 2.6 |
| TSy | 2 EtOH + $H_2$ + MeCHO | **25.3** | 25.4 | 25.7 | 24.2 | 29.3 | 29.0 | 22.8 | 26.4 | 22.0 |
| Ru-OEt-e | 2 EtOH + $H_2$ | **7.4** | 9.1 | 9.2 | 6.6 | 9.9 | 20.0 | 7.4 | 8.8 | 4.5 |
| **Addition of Explicit EtOH to Key Species in the Above Pathway (Note that the C-O cleavage and proton transfer steps are now separate)** | | | | | | | | | | |
| TSq-e-S | EtOH + $H_2$ | **26.0** | 27.9 | 28.2 | 25.7 | 22.3 | 39.5 | 22.5 | 26.1 | 22.7 |
| r-e-S | EtOH + $H_2$ | **23.3** | 25.2 | 24.9 | 24.6 | 24.9 | 39.1 | 22.1 | 23.1 | 19.9 |
| TSv-e-S | EtOH + $H_2$ | **22.9** | 24.5 | 23.5 | 28.1 | 25.4 | 39.5 | 26.4 | 22.9 | 15.7 |
| TSx-e-S | EtOH + $H_2$ | **28.1** | 30.0 | 29.8 | 27.3 | 29.3 | 44.8 | 27.1 | 30.1 | 23.2 |
| ao-e-S | EtOH + $H_2$ | **27.9** | 29.7 | 29.4 | 26.6 | 28.5 | 43.9 | 26.1 | 29.9 | 22.7 |
| TSao-e-S | EtOH + $H_2$ | **29.7** | 31.6 | 32.0 | 28.3 | 29.8 | 45.0 | 26.9 | 31.8 | 24.2 |
| TSy-e | EtOH + $H_2$ + MeCHO | **21.0** | 22.8 | 23.2 | 21.2 | 27.2 | 31.4 | 17.8 | 22.7 | 15.3 |
| **Alternative Mechanism to Regenerate the Ruthenium-alkoxide Without Undergoing Dearomatization, Fig. S15** | | | | | | | | | | |
| ao-e-S | EtOH + $H_2$ | **27.9** | 29.7 | 29.4 | 26.6 | 28.5 | 43.9 | 26.1 | 29.9 | 22.7 |
| b | EtOH + $H_2$ + MeCHO | **25.7** | 27.3 | 26.3 | 24.7 | 28.0 | 35.0 | 24.8 | 24.4 | 14.3 |
| TSa | EtOH + $H_2$ + MeCHO | **26.8** | 28.4 | 27.3 | 25.8 | 29.3 | 36.6 | 26.0 | 25.6 | 15.5 |
| Ru-OEt-e | 2 EtOH + $H_2$ | **7.4** | 9.1 | 9.2 | 6.6 | 9.9 | 20.0 | 7.4 | 8.8 | 4.5 |

| | | ωB97M-V | | | toluene | | | | | |
|---|---|---|---|---|---|---|---|---|---|---|
| | | toluene | THF | ethanol | M06L | MN15L | TPSS | TPSS_D4 | M06 | B3LYP-D3 |
| **Ester Hydrogenolysis Alternative 2 (Dearomatization), Protonate to Initially Release Free Hemiacetal, Fig S16** | | | | | | | | | | |
| r-R | 2 EtOH + $H_2$ | **27.1** | 27.5 | 24.9 | 25.8 | 26.0 | 37.6 | 24.1 | 25.0 | 21.6 |
| TSap-R | 2 EtOH + $H_2$ | **30.4** | 30.8 | 29.5 | 31.9 | 31.3 | 42.2 | 28.8 | 30.2 | 22.0 |
| Ru-de | 2 EtOH + $H_2$ + hemiacetal | **14.3** | 13.1 | 13.7 | 17.5 | 19.2 | 15.7 | 16.4 | 11.6 | 6.9 |
| aq-R | 2 EtOH + $H_2$ | **20.6** | 21.0 | 21.7 | 24.0 | 21.0 | 36.1 | 23.9 | 21.5 | 17.9 |
| TSar-R | 2 EtOH + $H_2$ | **26.4** | 26.8 | 27.4 | 30.1 | 29.3 | 40.3 | 26.7 | 28.5 | 26.4 |
| w-R | 2 EtOH + $H_2$ | **8.4** | 8.7 | 8.6 | 11.1 | 10.7 | 22.4 | 10.6 | 12.0 | 9.6 |
| **Addition of Explicit EtOH to Key Species in the Above Pathway** | | | | | | | | | | |
| TSap-e-R | EtOH + $H_2$ | **30.9** | 32.9 | 30.8 | 33.5 | 30.4 | 47.9 | 30.8 | 31.5 | 23.0 |
| TSar-e-R | EtOH + $H_2$ | **28.9** | 31.0 | 32.0 | 33.3 | 29.8 | 49.5 | 30.5 | 32.4 | 28.9 |
| **Protonate to Initially Release Free Hemiacetal, Different Carbon Configuration (S Hemiacetaloxide), Fig. S17** | | | | | | | | | | |
| r-S | 2 EtOH + $H_2$ | **26.4** | 26.8 | 24.0 | 25.7 | 26.5 | 35.9 | 23.3 | 24.8 | 22.5 |
| TSap-S | 2 EtOH + $H_2$ | **31.5** | 31.9 | 25.7 | 32.9 | 31.8 | 42.0 | 31.6 | 31.2 | 23.5 |
| Ru-de | 2 EtOH + $H_2$ + hemiacetal | **14.3** | 13.1 | 13.7 | 17.5 | 19.2 | 15.7 | 16.4 | 11.6 | 6.9 |
| aq-S | 2 EtOH + $H_2$ | **18.0** | 18.5 | 19.3 | 21.4 | 19.1 | 34.4 | 21.7 | 19.4 | 17.2 |
| TSar-S | 2 EtOH + $H_2$ | **23.8** | 24.3 | 24.8 | 27.7 | 27.5 | 37.8 | 24.3 | 26.9 | 24.0 |
| w-S | 2 EtOH + $H_2$ | **10.0** | 10.3 | 10.3 | 12.1 | 11.5 | 25.0 | 12.3 | 13.6 | 11.0 |
| **Addition of Explicit EtOH to Key Species in the Above Pathway** | | | | | | | | | | |
| TSap-e-S | EtOH + $H_2$ | **29.5** | 31.3 | 31.3 | 33.9 | 31.1 | 44.5 | 30.2 | 30.3 | 21.3 |
| TSar-e-S | EtOH + $H_2$ | **26.6** | 28.7 | 29.7 | 30.9 | 28.1 | 46.5 | 28.0 | 29.7 | 26.8 |
| **Ester Hydrogenolysis Alternative 2 (Dearomatization), Deprotonate Hemiacetal to Release Aldehyde, Fig. S18** | | | | | | | | | | |
| Ru-de | 2 EtOH + $H_2$ + hemiacetal | **14.3** | 13.1 | 13.7 | 17.5 | 19.2 | 15.7 | 16.4 | 11.6 | 6.9 |
| as-R | 2 EtOH + $H_2$ | **23.4** | 23.9 | 24.6 | 26.0 | 22.4 | 40.4 | 27.2 | 22.7 | 17.3 |
| TSat-R | 2 EtOH + $H_2$ | **30.5** | 31.1 | 29.0 | 32.0 | 33.4 | 45.2 | 29.1 | 32.4 | 22.7 |
| Ru-OEt-e | EtOH + $H_2$ + MeCHO | **7.4** | 9.1 | 9.2 | 6.6 | 9.9 | 20.0 | 7.4 | 8.8 | 4.5 |
| **Addition of Explicit EtOH to Key Species in the Above Pathway (Note that the proton transfer and C-O cleavage steps are now separate)** | | | | | | | | | | |
| TSat-e-R | EtOH + $H_2$ | **29.5** | 31.6 | 31.4 | 33.1 | 33.1 | 49.8 | 29.5 | 32.2 | 22.0 |
| au-e-R | EtOH + $H_2$ | **24.6** | 26.6 | 25.2 | 26.8 | 28.9 | 45.0 | 25.2 | 27.1 | 18.0 |
| TSav-e-R | EtOH + $H_2$ | **26.4** | 28.4 | 28.6 | 23.4 | 24.6 | 44.4 | 24.7 | 27.3 | 22.2 |
| **Deprotonate Hemiacetal to Release Aldehyde, Different Carbon Configuration (S Hemiacetaloxide), Fig. S19** | | | | | | | | | | |
| Ru-de | 2 EtOH + $H_2$ + hemiacetal | **14.3** | 13.1 | 13.7 | 17.5 | 19.2 | 15.7 | 16.4 | 11.6 | 6.9 |
| as-S | 2 EtOH + $H_2$ | **22.4** | 22.9 | 23.4 | 25.3 | 24.7 | 40.5 | 24.6 | 23.3 | 16.5 |
| TSat-S | 2 EtOH + $H_2$ | **29.3** | 29.9 | 28.0 | 30.6 | 32.6 | 43.6 | 27.4 | 31.5 | 26.7 |
| Ru-OEt-e | EtOH + $H_2$ + MeCHO | **7.4** | 9.1 | 9.2 | 6.6 | 9.9 | 20.0 | 7.4 | 8.8 | 4.5 |
| **Addition of Explicit EtOH to Key Species in the Above Pathway (Note that the proton transfer and C-O cleavage steps are now separate)** | | | | | | | | | | |
| TSat-e-S | EtOH + $H_2$ | **28.3** | 30.5 | 30.5 | 31.5 | 32.2 | 48.4 | 27.7 | 31.3 | 25.6 |
| au-e-S | EtOH + $H_2$ | **22.5** | 24.5 | 23.2 | 24.2 | 25.5 | 43.4 | 23.1 | 25.3 | 20.9 |
| TSav-e-S | EtOH + $H_2$ | **26.8** | 28.7 | 29.0 | 23.5 | 25.7 | 43.1 | 24.8 | 27.6 | 22.5 |

| | | ωB97M-V | | | toluene | | | | | |
|---|---|---|---|---|---|---|---|---|---|---|
| | | toluene | THF | ethanol | M06L | MN15L | TPSS | TPSS_D4 | M06 | B3LYP-D3 |
| **Ester Hydrogenolysis Alternative 2 (Dearomatization), Use $NCH_2$ Linker, Protonate to Release Ethanol, Fig. S20** | | | | | | | | | | |
| r-R | 2 EtOH + $H_2$ | **27.1** | 27.5 | 24.9 | 25.8 | 26.0 | 37.6 | 24.1 | 25.0 | 21.6 |
| TSap-n-R | 2 EtOH + $H_2$ | **34.9** | 35.3 | 34.1 | 35.9 | 36.7 | 44.1 | 32.9 | 34.2 | 26.8 |
| Ru-de-n | 2 EtOH + $H_2$ + hemiacetal | **23.7** | 22.5 | 23.0 | 26.3 | 28.4 | 23.8 | 25.1 | 20.0 | 15.7 |
| aq-n-R | 2 EtOH + $H_2$ | **24.5** | 25.0 | 25.7 | 27.7 | 27.5 | 38.4 | 26.4 | 25.3 | 23.1 |
| TSar-n-R | 2 EtOH + $H_2$ | **25.9** | 26.3 | 27.2 | 29.8 | 30.4 | 37.6 | 25.2 | 27.9 | 24.9 |
| w-R | 2 EtOH + $H_2$ | **8.4** | 8.7 | 8.6 | 11.1 | 10.7 | 22.4 | 10.6 | 12.0 | 9.6 |
| TSx-n-R | 2 EtOH + $H_2$ | **35.6** | 36.0 | 36.4 | 33.0 | 39.3 | 41.5 | 29.2 | 34.8 | 29.6 |
| Ru-de-n | 3 EtOH + $H_2$ + MeCHO | **22.8** | 21.2 | 21.1 | 19.9 | 26.1 | 17.6 | 22.0 | 16.9 | 11.4 |
| **Addition of Explicit EtOH to Key Species in the Above Pathway** | | | | | | | | | | |
| TSap-n-e-R | EtOH + $H_2$ | **35.1** | 37.1 | 37.3 | 38.0 | 38.2 | 48.8 | 33.9 | 35.1 | 26.8 |
| TSar-n-e-R | EtOH + $H_2$ | **28.5** | 30.6 | 31.5 | 32.3 | 30.5 | 45.8 | 28.7 | 30.6 | 27.6 |
| TSx-n-e-R | EtOH + $H_2$ | **34.7** | 36.8 | 37.1 | 32.6 | 35.3 | 49.9 | 30.8 | 34.1 | 28.8 |
| **Use $NCH_2$ Linker, Protonate to Release Ethanol, Different Carbon Configuration (S Hemiacetaloxide), Fig S21** | | | | | | | | | | |
| r-S | 2 EtOH + $H_2$ | **26.4** | 26.8 | 24.0 | 25.7 | 26.5 | 35.9 | 23.3 | 24.8 | 22.5 |
| TSap-n-S | 2 EtOH + $H_2$ | **33.3** | 33.8 | 32.9 | 35.0 | 35.0 | 43.8 | 30.9 | 32.6 | 25.4 |
| Ru-de-n | 2 EtOH + $H_2$ + hemiacetal | **23.7** | 22.5 | 23.0 | 26.3 | 28.4 | 23.8 | 25.1 | 20.0 | 15.7 |
| aq-n-S | 2 EtOH + $H_2$ | **25.5** | 26.0 | 26.7 | 29.0 | 29.0 | 38.3 | 27.1 | 26.1 | 24.2 |
| TSar-n-S | 2 EtOH + $H_2$ | **26.4** | 26.8 | 27.4 | 30.0 | 30.5 | 37.9 | 25.9 | 27.9 | 25.4 |
| w-S | 2 EtOH + $H_2$ | **10.0** | 10.3 | 10.3 | 12.1 | 11.5 | 25.0 | 12.3 | 13.6 | 11.0 |
| TSx-n-S | 2 EtOH + $H_2$ | **36.1** | 36.5 | 36.5 | 32.5 | 36.9 | 43.5 | 30.5 | 35.7 | 30.0 |
| Ru-de-n | 3 EtOH + $H_2$ + MeCHO | **22.8** | 21.2 | 21.1 | 19.9 | 26.1 | 17.6 | 22.0 | 16.9 | 11.4 |
| **Addition of Explicit EtOH to Key Species in the Above Pathway** | | | | | | | | | | |
| TSap-n-e-S | EtOH + $H_2$ | **34.8** | 36.9 | 36.9 | 38.0 | 36.5 | 50.9 | 33.6 | 34.5 | 26.5 |
| TSar-n-e-S | EtOH + $H_2$ | **28.8** | 30.9 | 32.0 | 33.1 | 30.7 | 46.3 | 29.3 | 30.8 | 27.9 |
| TSx-n-e-S | EtOH + $H_2$ | **32.2** | 34.2 | 34.9 | 30.6 | 32.1 | 45.7 | 28.4 | 33.6 | 26.3 |
| **Ester Hydrogenolysis Alternative 2 (Dearomatization), Use $NCH_2$ Linker, Deprotonate to Release Aldehyde, Fig. S22** | | | | | | | | | | |
| Ru-de-n | 2 EtOH + $H_2$ + hemiacetal | **23.7** | 22.5 | 23.0 | 26.3 | 28.4 | 23.8 | 25.1 | 20.0 | 15.7 |
| as-n-R | 2 EtOH + $H_2$ | **28.9** | 29.4 | 29.8 | 31.5 | 31.6 | 44.6 | 29.8 | 29.1 | 23.8 |
| TSat-n-R | 2 EtOH + $H_2$ | **30.9** | 31.5 | 30.1 | 32.2 | 33.3 | 43.8 | 28.7 | 32.2 | 28.5 |
| au-n-R | 2 EtOH + $H_2$ | **24.8** | 25.3 | 22.1 | 23.2 | 25.3 | 37.5 | 22.4 | 26.7 | 22.5 |
| TSav-n-R | 2 EtOH + $H_2$ | **25.3** | 25.7 | 23.0 | 23.3 | 25.6 | 37.8 | 22.7 | 27.0 | 22.6 |
| Ru-OEt-e | EtOH + $H_2$ + MeCHO | **7.4** | 9.1 | 9.2 | 6.6 | 9.9 | 20.0 | 7.4 | 8.8 | 4.5 |
| **Addition of Explicit EtOH to Key Species in the Above Pathway** | | | | | | | | | | |
| TSat-n-e-R | EtOH + $H_2$ | **31.8** | 33.9 | 34.2 | 34.6 | 34.7 | 50.1 | 30.7 | 33.4 | 29.0 |
| au-n-e-R | EtOH + $H_2$ | **21.9** | 23.8 | 22.9 | 23.4 | 24.0 | 41.4 | 22.2 | 24.8 | 20.6 |
| TSav-n-e-R | EtOH + $H_2$ | **23.2** | 25.2 | 25.9 | 20.5 | 20.7 | 41.0 | 22.7 | 24.2 | 19.9 |
| **Use $NCH_2$ Linker, Deprotonate to Release Aldehyde, Different Carbon Configuration (S Hemiacetaloxide), Fig. S23** | | | | | | | | | | |
| Ru-de-n | 2 EtOH + $H_2$ + hemiacetal | **23.7** | 22.5 | 23.0 | 26.3 | 28.4 | 23.8 | 25.1 | 20.0 | 15.7 |
| as-n-S | 2 EtOH + $H_2$ | **29.7** | 30.3 | 30.6 | 33.4 | 33.1 | 46.0 | 31.2 | 29.2 | 22.1 |
| TSat-n-S | 2 EtOH + $H_2$ | **33.0** | 33.6 | 31.9 | 35.0 | 36.6 | 46.0 | 30.5 | 33.3 | 29.7 |
| Ru-OEt-e | EtOH + $H_2$ + MeCHO | **7.4** | 9.1 | 9.2 | 6.6 | 9.9 | 20.0 | 7.4 | 8.8 | 4.5 |
| **Addition of Explicit EtOH to Key Species in the Above Pathway (Note that the proton transfer and C-O cleavage steps are now separate)** | | | | | | | | | | |
| TSat-n-e-S | EtOH + $H_2$ | **33.7** | 35.8 | 36.2 | 37.4 | 37.6 | 52.3 | 32.4 | 34.4 | 30.1 |
| au-n-e-S | EtOH + $H_2$ | **22.6** | 24.6 | 23.7 | 25.3 | 26.6 | 41.6 | 22.4 | 24.7 | 20.3 |
| TSav-n-e-S | EtOH + $H_2$ | **22.9** | 24.8 | 25.6 | 21.2 | 23.8 | 38.7 | 21.7 | 24.3 | 19.4 |

| Aldehyde Reduction Alternative Pathways | | ωB97M-V | | | toluene | | | | | |
|---|---|---|---|---|---|---|---|---|---|---|
| | | toluene | THF | ethanol | M06L | MN15L | TPSS | TPSS_D4 | M06 | B3LYP-D3 |
| **Variation on the Hasanayn-type MEP Without Explicit EtOH, Fig. S24** | | | | | | | | | | |
| Ru-H | 3 EtOH + MeCHO | **5.4** | 3.7 | 3.3 | 4.9 | 10.5 | 6.7 | 8.7 | 5.8 | 4.8 |
| TSz | 3 EtOH | **21.3** | 20.0 | 18.4 | 19.3 | 24.6 | 26.4 | 19.5 | 20.8 | 16.8 |
| aa | 3 EtOH | **23.4** | 22.2 | 15.8 | 18.8 | 25.7 | 27.6 | 20.2 | 20.8 | 17.4 |
| TSab | 3 EtOH | **28.0** | 26.8 | 19.4 | 26.1 | 34.9 | 35.9 | 30.6 | 27.4 | 19.4 |
| Ru-OEt-e | 2 EtOH | **-1.5** | -1.2 | -1.0 | 0.4 | 6.9 | 16.6 | 3.3 | 1.5 | -1.9 |
| **Dechelation/1,2-Insertion Pathway with Aldehyde Oxygen Trans to Hydride (Wang), Fig. S25** | | | | | | | | | | |
| Ru-H | 3 EtOH + MeCHO | **5.4** | 3.7 | 3.3 | 4.9 | 10.5 | 6.7 | 8.7 | 5.8 | 4.8 |
| TSae | 3 EtOH + MeCHO | **29.4** | 27.8 | 27.4 | 23.0 | 32.4 | 28.4 | 32.9 | 22.8 | 20.9 |
| af | 3 EtOH + MeCHO | **25.1** | 23.5 | 22.8 | 21.5 | 33.2 | 22.3 | 30.6 | 19.1 | 17.7 |
| aw | 3 EtOH | **13.9** | 12.5 | 12.5 | 12.8 | 19.3 | 16.6 | 18.9 | 14.1 | 13.6 |
| TSax | 3 EtOH | **17.0** | 15.6 | 15.1 | 17.3 | 24.0 | 21.4 | 23.6 | 17.8 | 17.5 |
| o | 3 EtOH | **11.3** | 9.9 | 9.2 | 7.5 | 19.2 | 13.1 | 14.3 | 8.2 | 4.0 |
| TSp | 3 EtOH | **16.4** | 15.0 | 14.4 | 10.4 | 19.8 | 21.7 | 18.5 | 12.9 | 8.3 |
| Ru-OEt-e | 2 EtOH | **-1.5** | -1.2 | -1.0 | 0.4 | 6.9 | 16.6 | 3.3 | 1.5 | -1.9 |
| **Dechelation/1,2-Insertion Pathway with Aldehyde Oxygen Trans to Phosphorus (Zhang), Fig. S26** | | | | | | | | | | |
| Ru-H | 3 EtOH + MeCHO | **5.4** | 3.7 | 3.3 | 4.9 | 10.5 | 6.7 | 8.7 | 5.8 | 4.8 |
| TSe | 3 EtOH + MeCHO | **31.6** | 29.9 | 29.3 | 27.3 | 35.3 | 31.9 | 36.2 | 29.2 | 25.0 |
| f | 3 EtOH + MeCHO | **25.2** | 23.5 | 22.9 | 21.4 | 29.9 | 21.8 | 28.7 | 22.1 | 18.3 |
| ay | 3 EtOH | **21.3** | 19.9 | 19.1 | 20.2 | 26.8 | 23.3 | 25.1 | 22.6 | 21.5 |
| TSaz | 3 EtOH | **20.9** | 19.5 | 18.6 | 20.7 | 27.6 | 23.9 | 25.6 | 22.5 | 21.6 |
| o | 3 EtOH | **11.3** | 9.9 | 9.2 | 7.5 | 19.2 | 13.1 | 14.3 | 8.2 | 4.0 |
| TSp | 3 EtOH | **16.4** | 15.0 | 14.4 | 10.4 | 19.8 | 21.7 | 18.5 | 12.9 | 8.3 |
| Ru-OEt-e | 2 EtOH | **-1.5** | -1.2 | -1.0 | 0.4 | 6.9 | 16.6 | 3.3 | 1.5 | -1.9 |
| **Hydride Attack Followed by Proton Transfer from PCH2 Linker with Dearomatization, Fig. S27** | | | | | | | | | | |
| Ru-H | 3 EtOH + MeCHO | **5.4** | 3.7 | 3.3 | 4.9 | 10.5 | 6.7 | 8.7 | 5.8 | 4.8 |
| TSz | 3 EtOH | **21.3** | 20.0 | 18.4 | 19.3 | 24.6 | 26.4 | 19.5 | 20.8 | 16.8 |
| aa | 3 EtOH | **23.4** | 22.2 | 15.8 | 18.8 | 25.7 | 27.6 | 20.2 | 20.8 | 17.4 |
| TSba | 3 EtOH | **27.7** | 26.5 | 22.6 | 26.1 | 34.7 | 34.2 | 26.8 | 26.3 | 18.2 |
| Ru-de | 4 EtOH | **4.5** | 1.6 | 1.5 | 4.8 | 13.9 | 6.1 | 9.1 | 1.3 | -3.8 |
| **Addition of Explicit EtOH to Key Species in the Above Pathway** | | | | | | | | | | |
| aa-e | 2 EtOH | **15.7** | 15.9 | 13.3 | 13.7 | 19.9 | 27.3 | 15.4 | 15.3 | 10.9 |
| TSba-e | 2 EtOH | **23.6** | 23.9 | 22.7 | 23.8 | 31.0 | 35.5 | 25.3 | 24.1 | 14.0 |
| Ru-de | 4 EtOH | **4.5** | 1.6 | 1.5 | 4.8 | 13.9 | 6.1 | 9.1 | 1.3 | -3.8 |

| Recalculation of the thermodynamics with hexyl hexanoate | | ωB97M-V | | | toluene | | | | | |
|---|---|---|---|---|---|---|---|---|---|---|
| **Organic Thermodynamics** | **Mass balance** | **toluene** | **THF** | **ethanol** | **M06L** | **MN15L** | **TPSS** | **TPSS_D4** | **M06** | **B3LYP-D3** |
| hexyl_hexanoate | 2 $H_2$ | **0.0** | 0.0 | 0.0 | 0.0 | 0.0 | 0.0 | 0.0 | 0.0 | 0.0 |
| hexanal | EtOH + H2 | **4.0** | 2.4 | 1.9 | 3.1 | 8.8 | 5.0 | 7.3 | 4.0 | 3.3 |
| hexanol | EtOH | **-4.7** | -7.6 | -8.1 | -2.5 | 6.4 | 1.8 | 3.4 | -2.9 | -2.8 |

**Recalculation of the energetic span using hexyl hexanoate**

| | | | | | | | | | | |
|---|---|---|---|---|---|---|---|---|---|---|
| Ru-H | 2 EtOH + $H_2$ + EtOAc | **0.0** | 0.0 | 0.0 | 0.0 | 0.0 | 0.0 | 0.0 | 0.0 | 0.0 |
| TSh-S_hexyl | 2 EtOH + $H_2$ | **26.0** | 26.3 | 26.6 | 28.2 | 23.1 | 41.8 | 29.7 | 29.4 | 28.3 |

## Alternative Pathways for Hydrogen Activation

Figure S1 estimates the barrier for hydrogen activation in an analogous manner to the MEP shown in Figure 7, without an explicit molecule of ethanol. Although a transition state for cleavage of the H-H bond was not located, the barrier for hydrogen activation without explicit ethanol, must be as least as high as that of the intermediate **c-e** at 35.9 kcal/mol which is much higher than the barrier of 21.4 in the corresponding pathway with explicit ethanol shown in Figure 7.

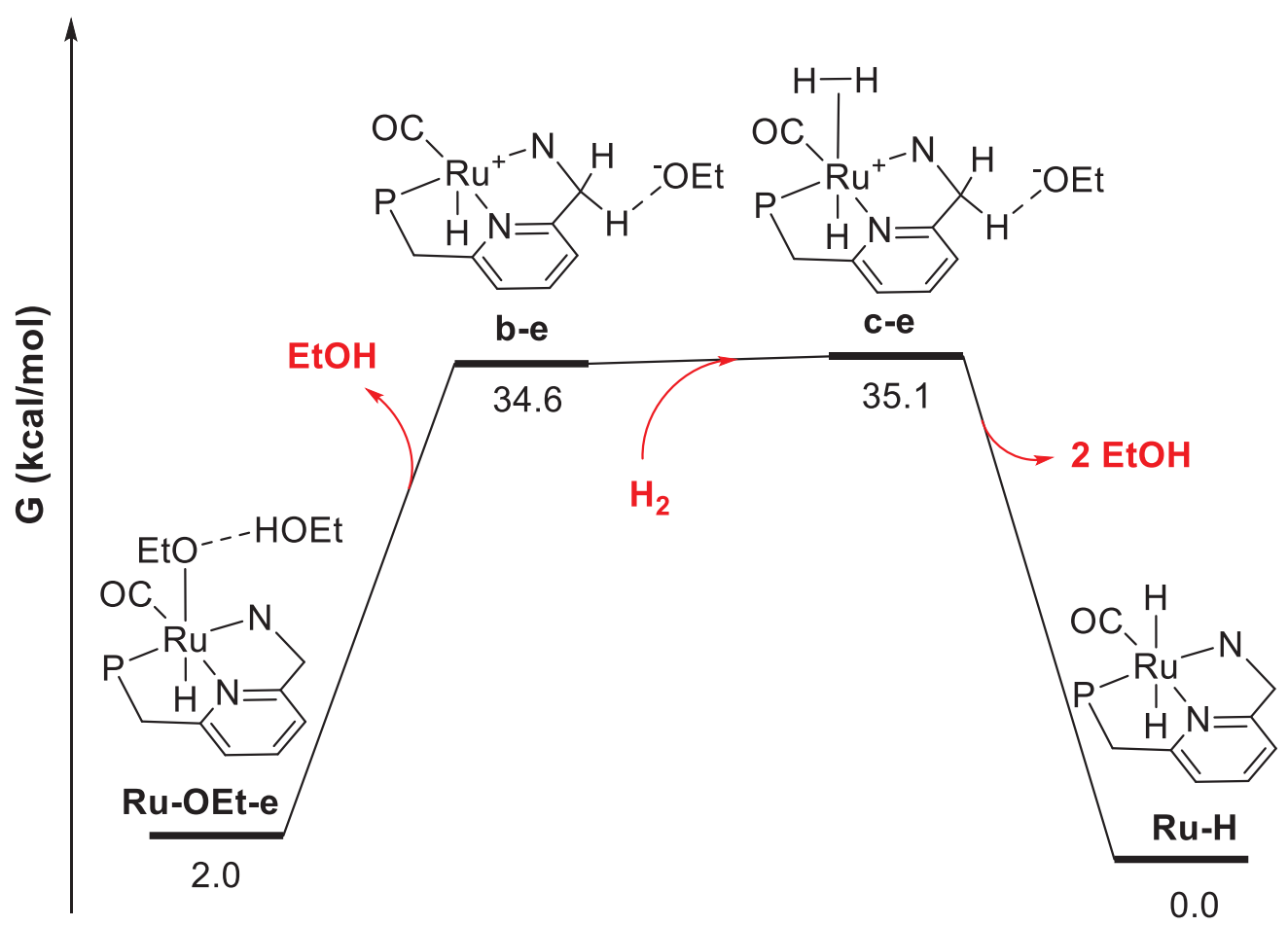


**Figure S1.** Deprotonation of $H_2$ by Ethoxide - No Explicit EtOH

Figure S2 shows an alternative pathway for hydrogen activation where the dearomatized complex **Ru-de** acts to cooperatively cleave the H-H bond. Solid black lines show the sequence with no explicit ethanol, and the dotted lines in teal show the pathway with an ethanol molecule added as a proton shuttle. This mechanism proceeds with a barrier of at least 29.1 kcal/mol, much higher than the barrier of 21.4 in the MEP shown in Figure 7.

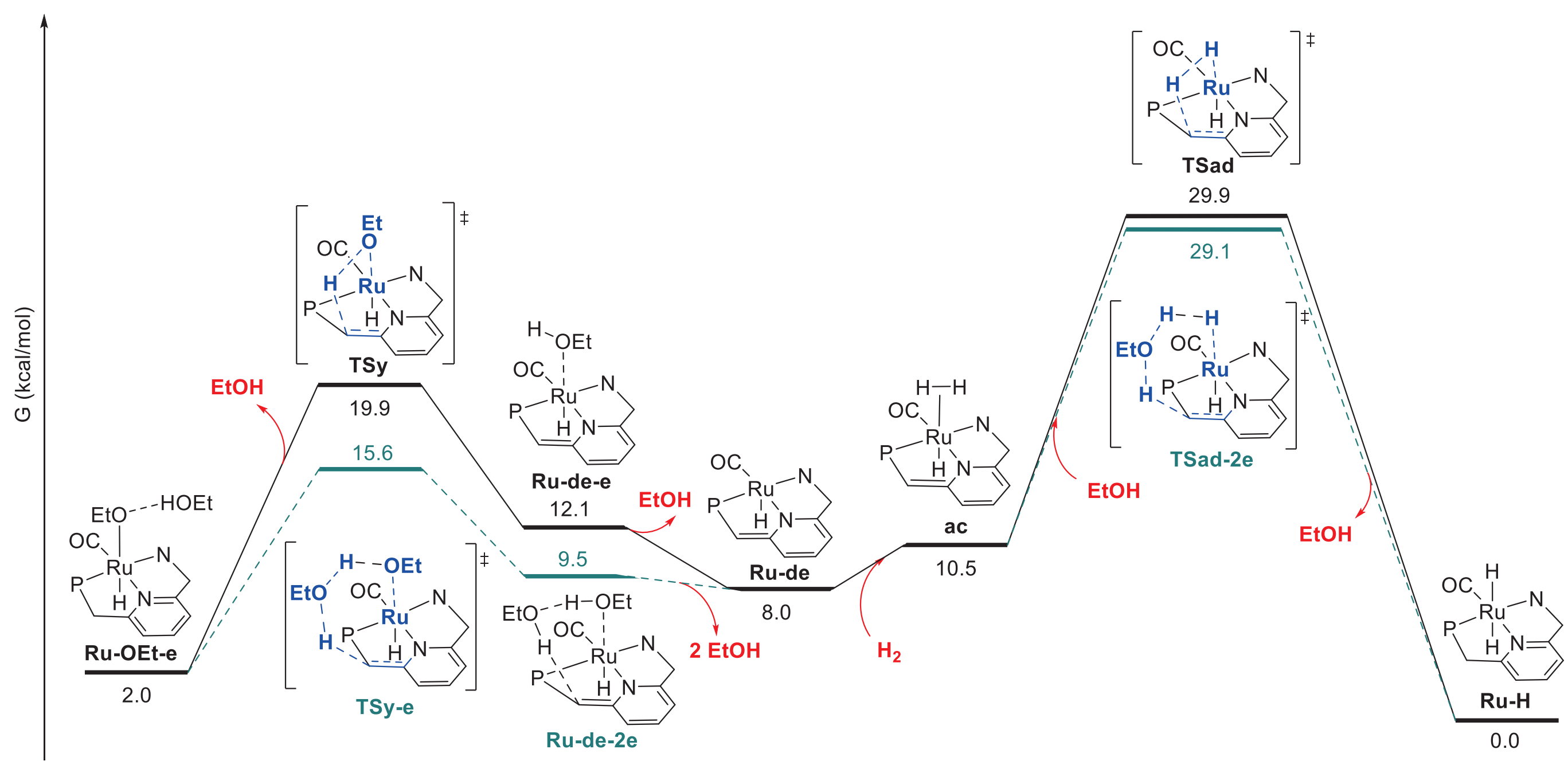


**Figure S2.** Metal-ligand-cooperative hydrogen activation mediated by the dearomatized complex **Ru-de**.

Figure S3 shows an alternative pathway for hydrogen activation where the $NCH_2$ linker is initially deprotonated instead of the $PCH_2$ linker. Although the barrier for H-H bond cleavage is reduced to 27.9 kcal/mol in this pathway, this is still significantly higher than the barrier of 21.4 kcal/mol in the MEP shown in Figure 7.

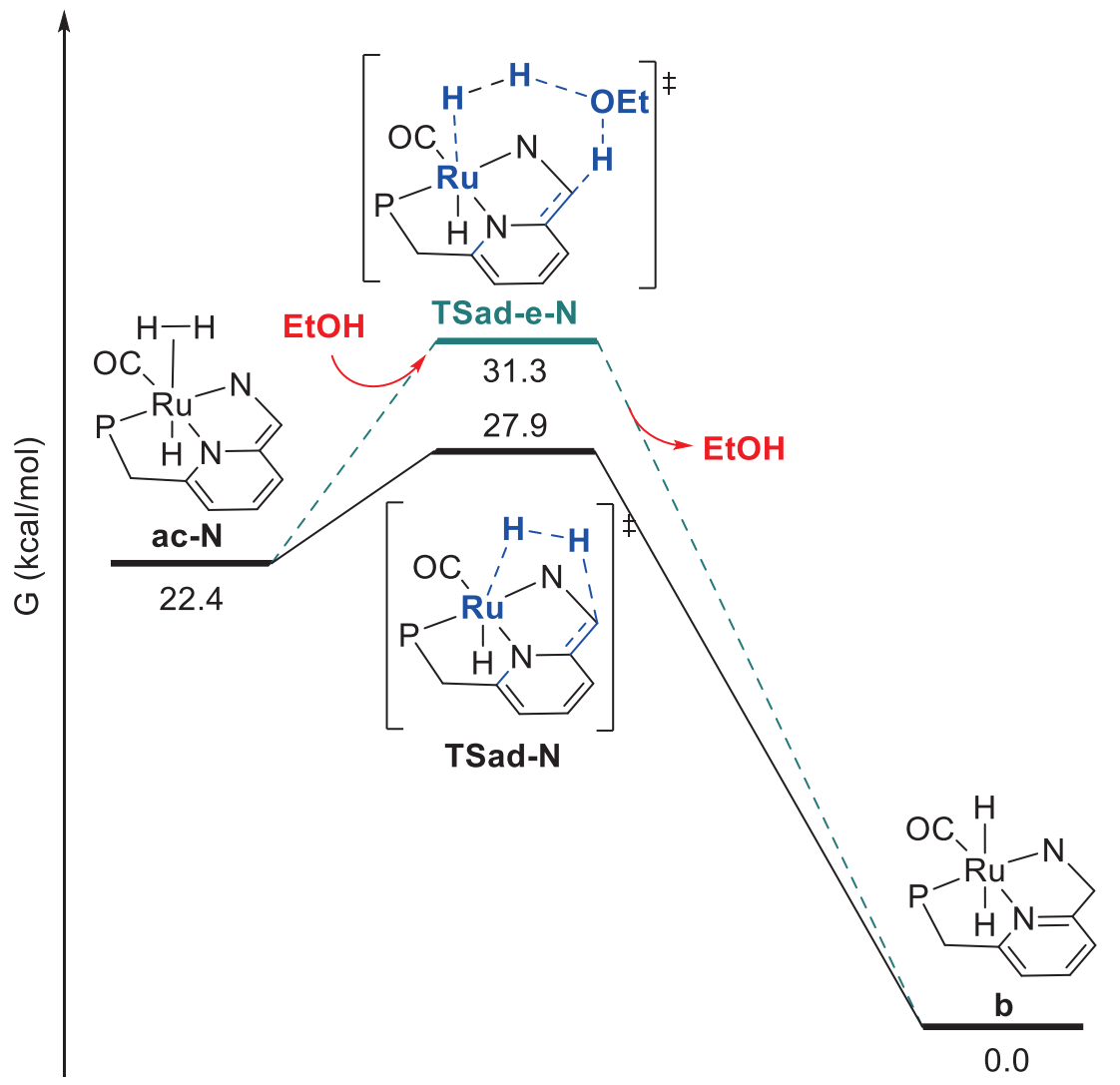


**Figure S3.** Metal-ligand-cooperative hydrogen activation via the $NCH_2$ linker.

## Alternative Pathways for Ester Hydrogenolysis Involving Dechelation and Inner-sphere Insertion

Figure S4 shows an alternative mechanism for hydride insertion in the ester hydrogenolysis pathway, which is the same as the MEP shown in Figure 8 except for the stereochemical configuration at ruthenium: here, the ester oxygen is trans to the ruthenium hydride instead of phosphorus. This pathway has a slightly higher barrier of 28.3 kcal/mol, compared to 26.5 for the MEP in Figure 8.

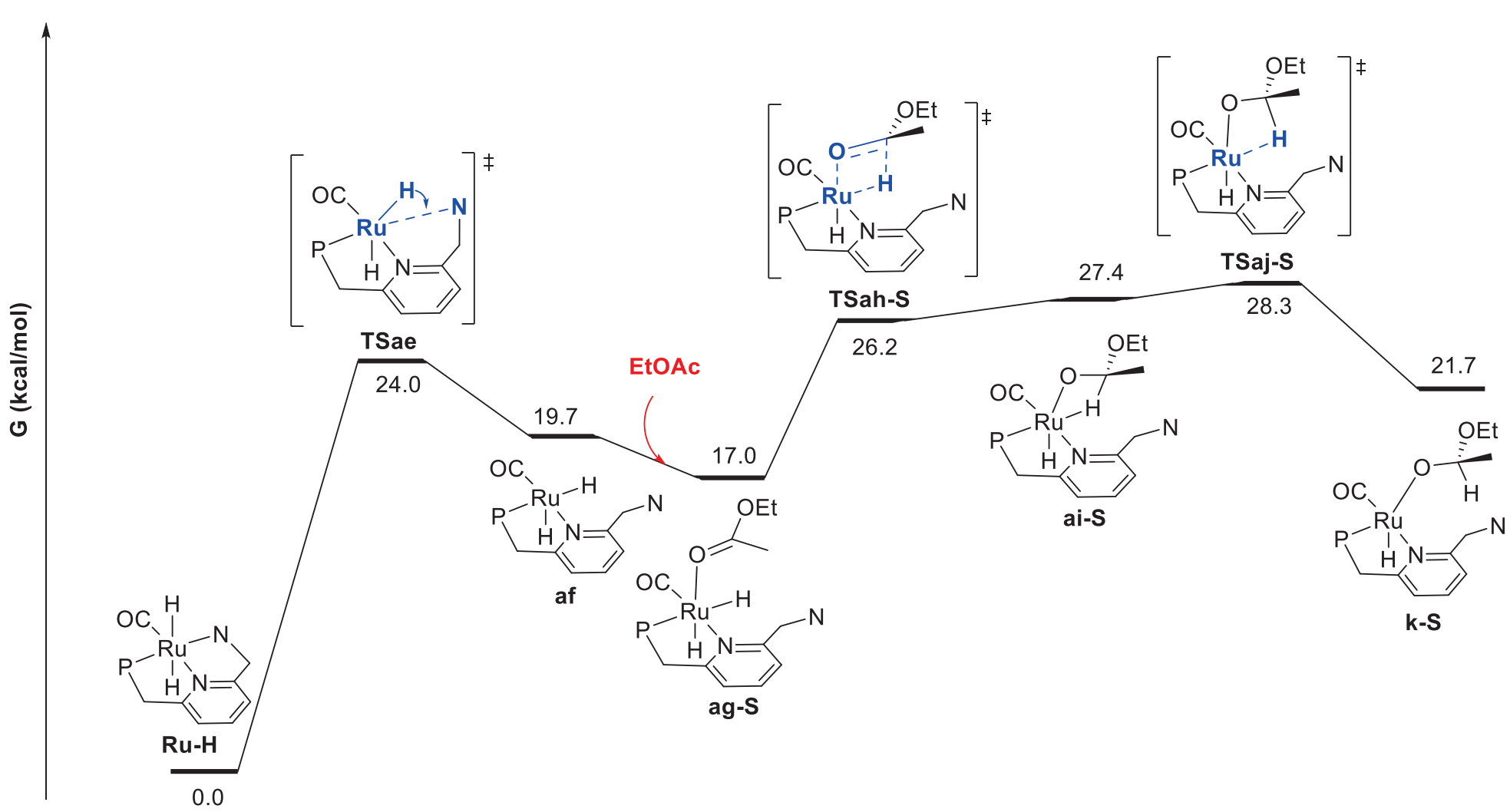


**Figure S4.** Inner-sphere hydride insertion with a different Ru configuration (O trans to hydride).

Figure S5 shows an alternative mechanism for hydride insertion in the ester hydrogenolysis pathway, which is the same as the MEP shown in Figure 8 except for the stereochemical configuration at the stereogenic carbon center. Here, the hemiacetaloxide carbon has an R configuration, while it is S in the MEP in Figure 8. This pathway has a slightly higher overall barrier of 27.0 kcal/mol, compared with 26.5 in the MEP.

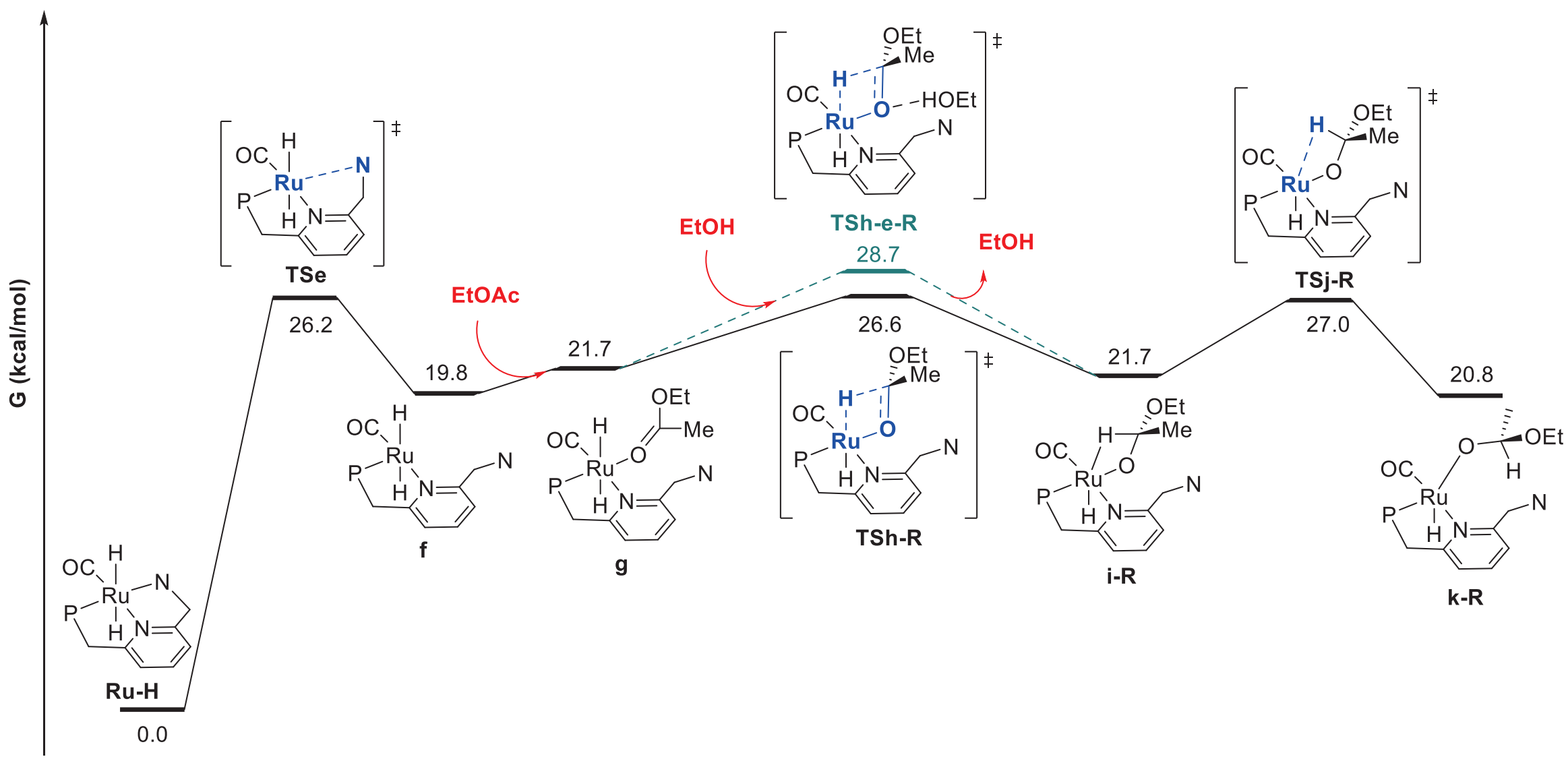


**Figure S5.** Inner-sphere hydride insertion with a different carbon configuration.

Figure S6 shows alternative mechanism for hydride insertion in the ester hydrogenolysis pathway, where the configurations at Ru and C are both different than those in the MEP in Figure 8. The overall barrier is 26.6 kcal/mol, almost the same the barrier of 26.5 kcal/mol as in the MEP.

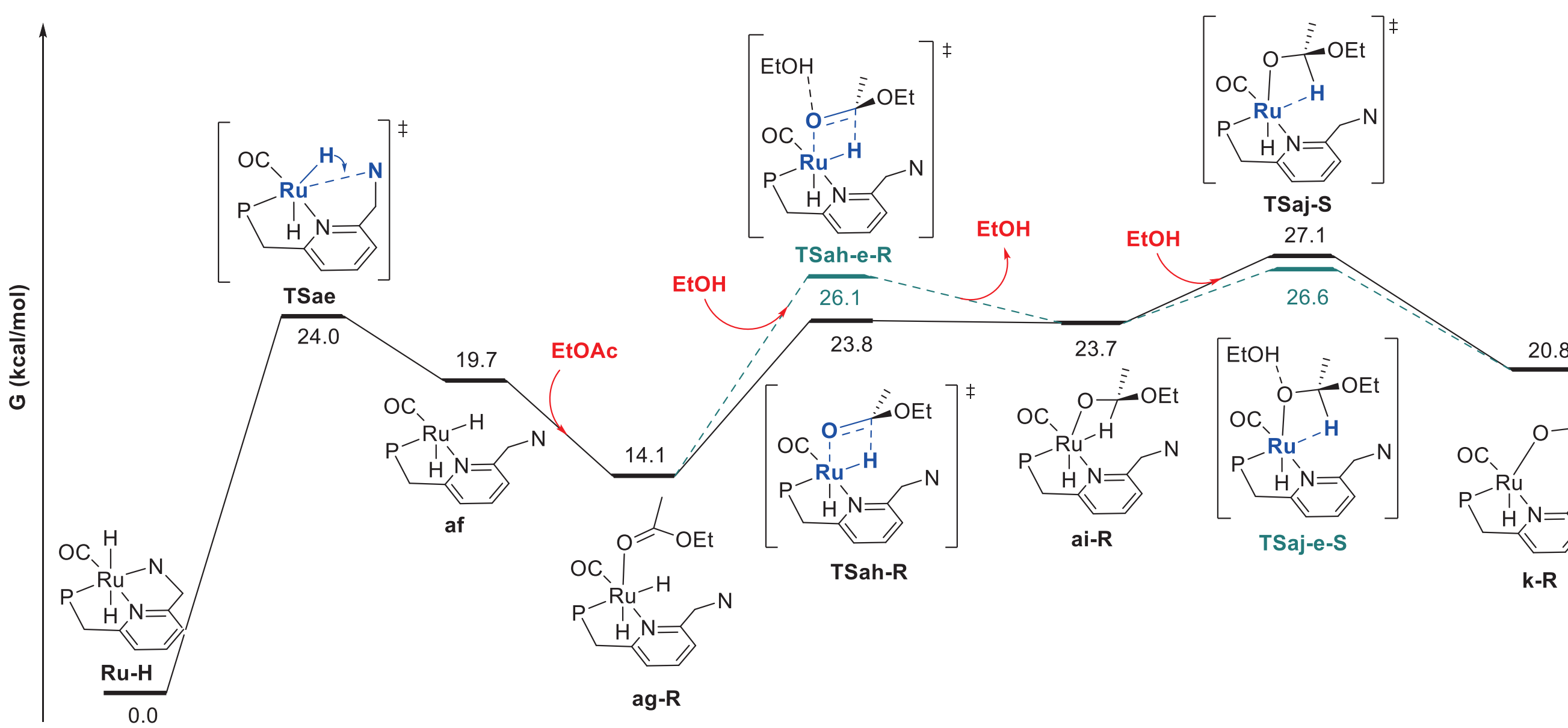


**Figure S6.** Inner-sphere hydride insertion with a different configurations at both ruthenium and carbon.

Figure S7 shows an alternative mechanism for C-O cleavage in the ester hydrogenolysis pathway shown in Figure 8, where the configuration at ruthenium is different. Here, the ethoxy group is trans to the hydride instead of phosphorus. This pathway has an overall barrier of 29.1 kcal/mol, higher than the barrier of 25.3 kcal/mol for C-O cleavage in the MEP in Figure 8.

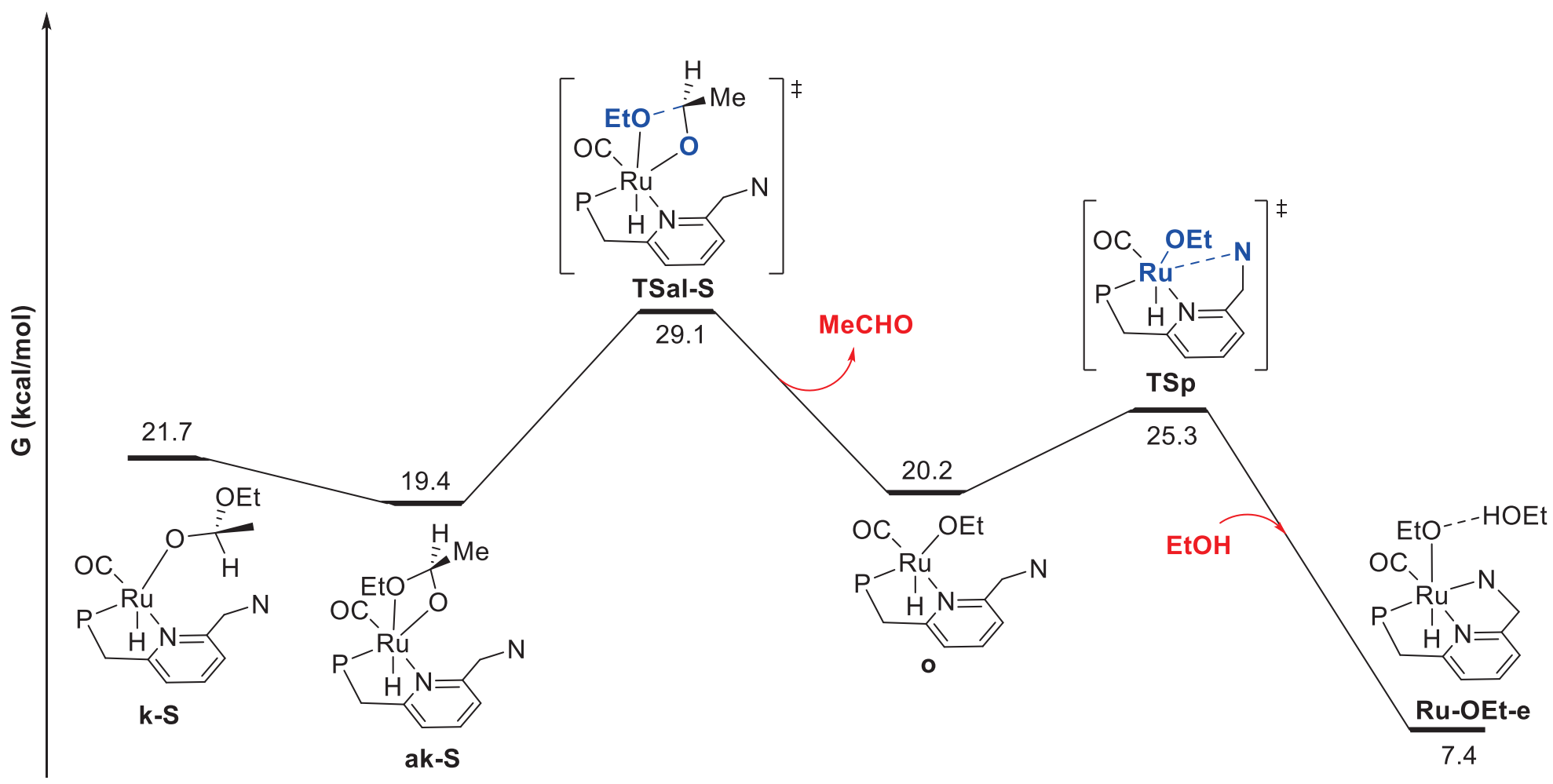


**Figure S7.** C-O bond cleavage with a different configuration at ruthenium (OEt trans to hydride).

Figure S8 shows an alternative mechanism for C-O cleavage in the ester hydrogenolysis pathway shown in Figure 8, where the configuration at the stereogenic carbon is R instead of S. This pathway has the same barrier of 25.3 kcal/mol compared with the C-O cleavage portion of the MEP shown in Figure 8.

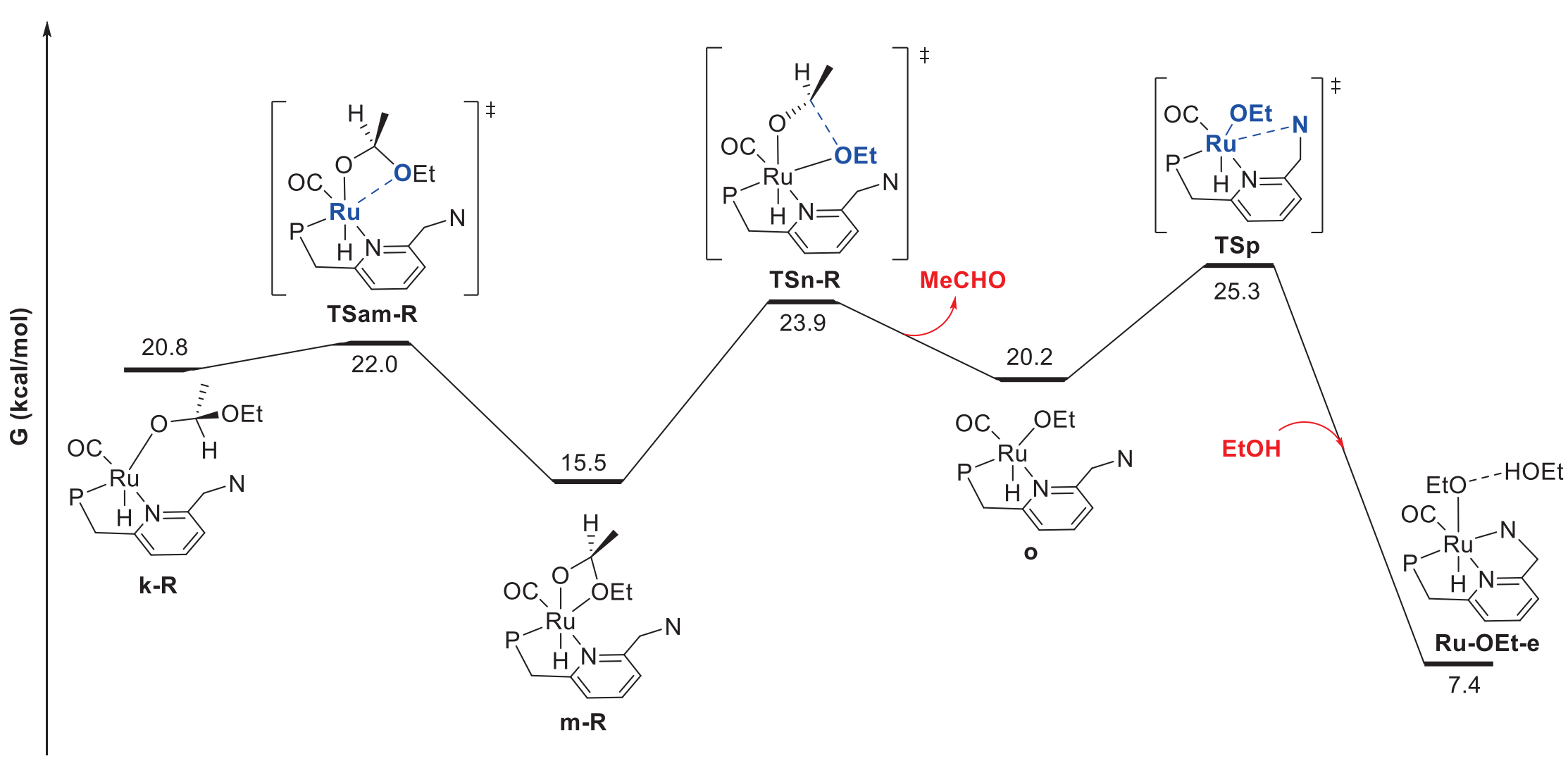


**Figure S8.** C-O bond cleavage with an R configuration at carbon.

Figure S9 shows an alternative mechanism for C-O cleavage in the ester hydrogenolysis pathway shown in Figure 8, where the configuration is different at both ruthenium and carbon. Because this pathway has a much higher barrier of 33.0 kcal/mol compared with 25.3 in the MEP in Figure 8, we did not seek to locate the transition state connecting **k-R** and **ak-R**, where the ethoxy oxygen binds to ruthenium.

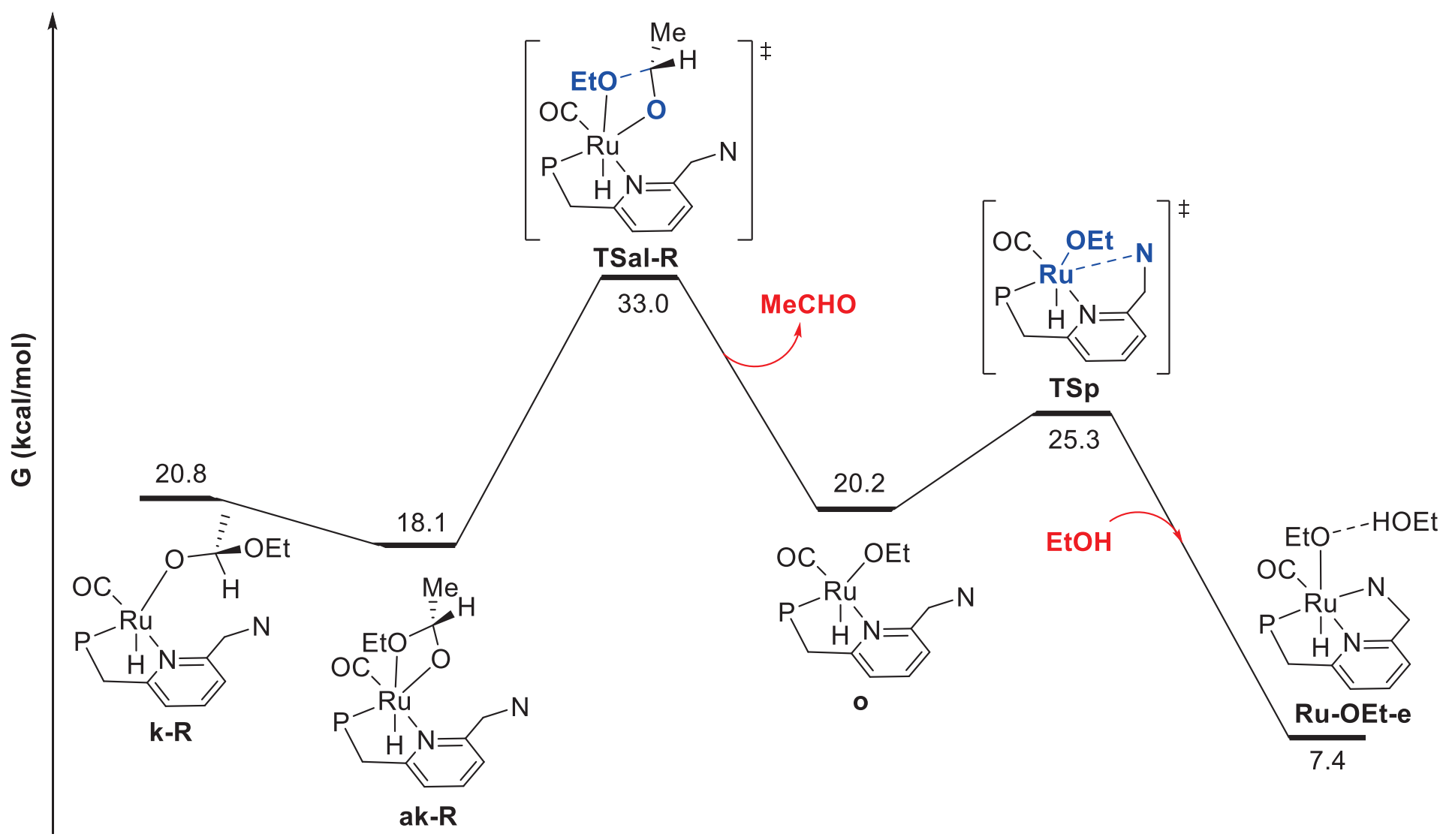


**Figure S9.** C-O bond with a different configurations at both ruthenium and carbon.

In addition to the above pathways where the C-O bond is cleaved with both hemiacetal oxygens remaining bound to ruthenium, we identified an alternative pathway where the aldehyde dissociation from Ru is concerted with C-O bond cleavage. This pathway, shown in Figure S10, had a higher overall barrier for C-O cleavage of 26.7 kcal/mol, compared with 25.3 kcal/mol in the MEP shown in Figure 8.

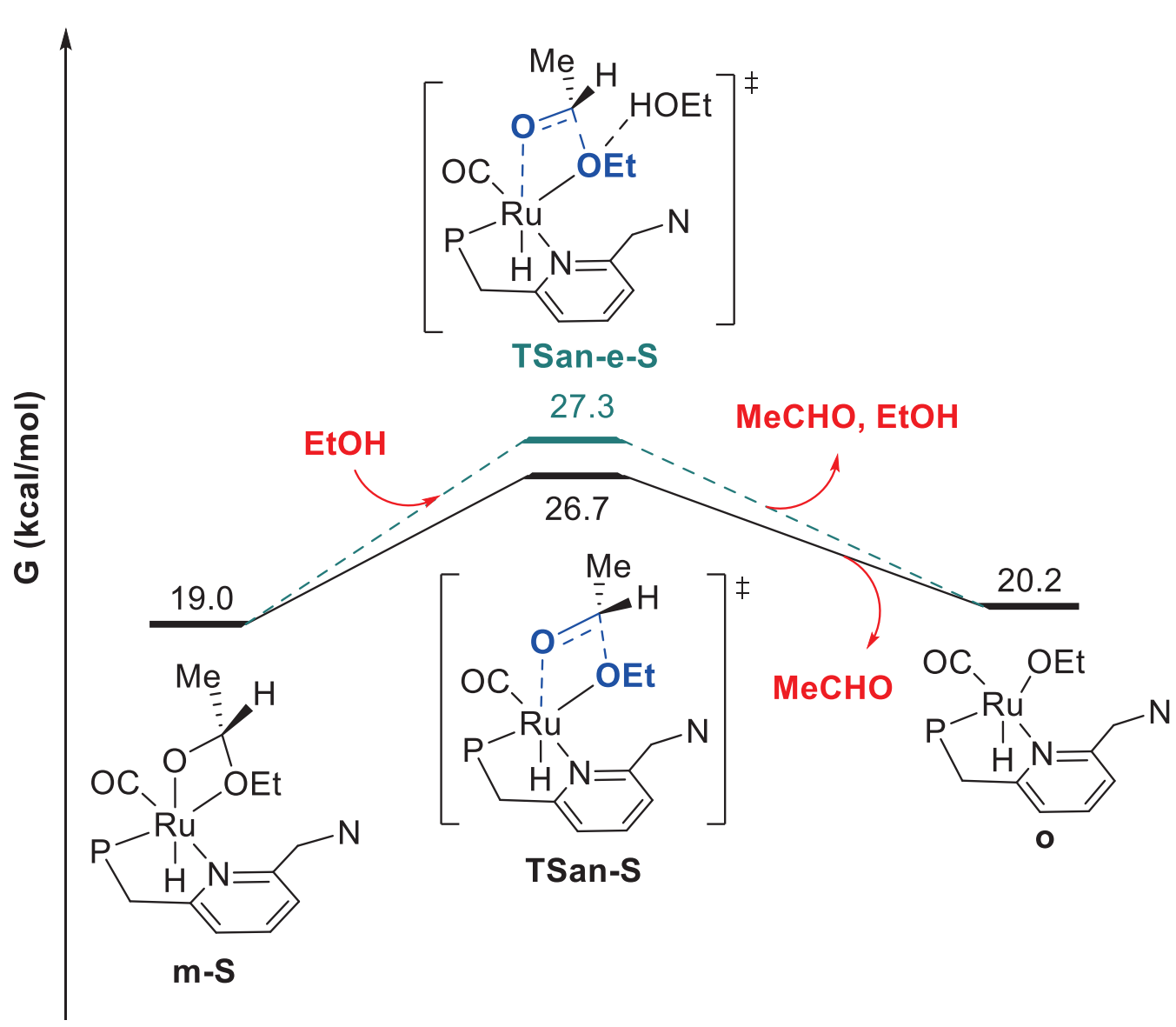


**Figure S10.** C-O bond cleavage concerted with aldehyde dissociation, S configuration at carbon.

Figure S11 shows a diastereomer of the pathway in Figure S10, where the carbon configuration is R instead of S. This pathway also has a higher overall barrier of 27.0 kcal/mol compared with the C-O cleavage barrier of 25.3 kcal/mol in the MEP shown in Figure 8.

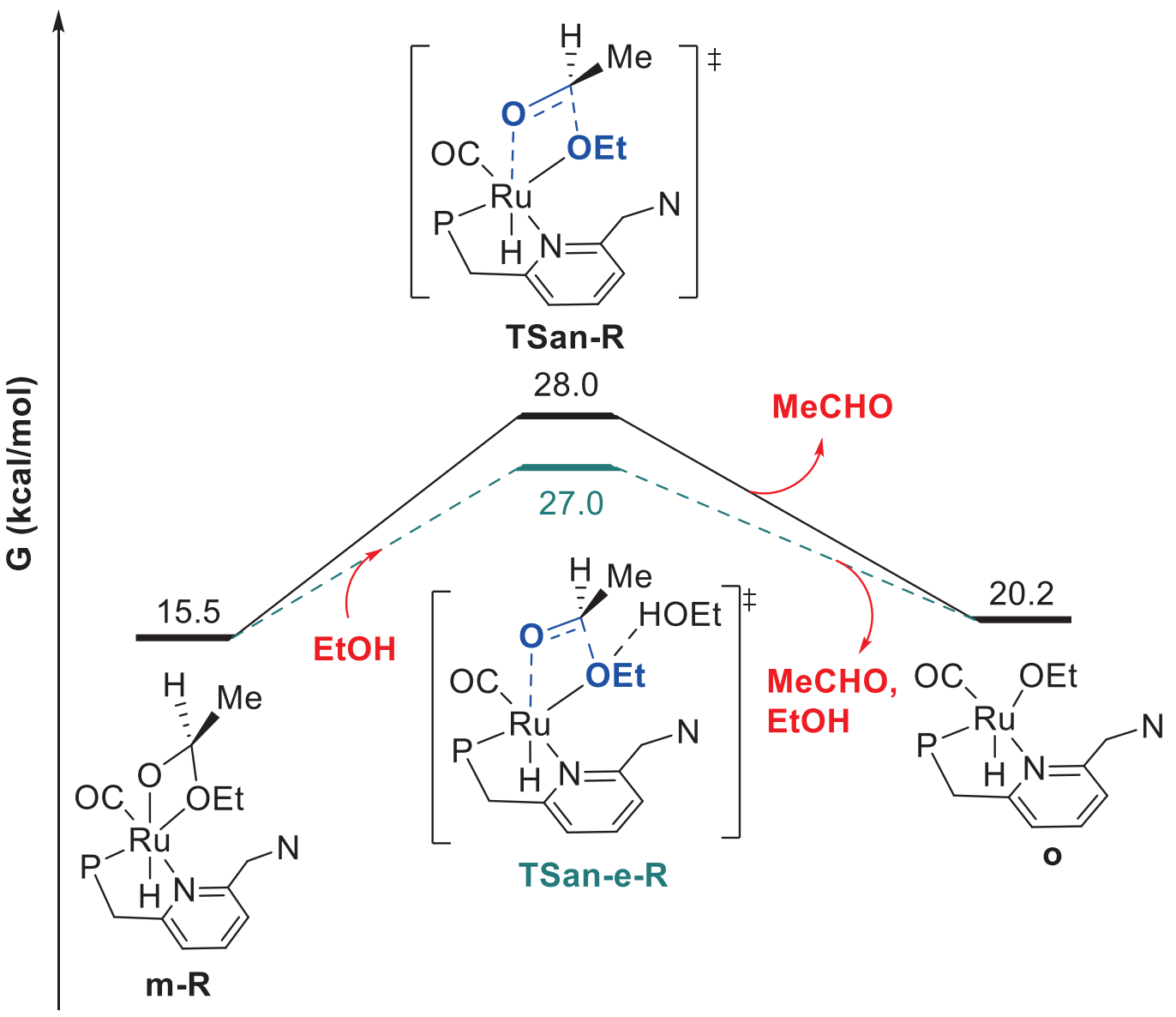


**Figure S11.** C-O bond cleavage concerted with aldehyde dissociation, R configuration at carbon.

## Alternative Pathways for Ester Hydrogenolysis Involving CH/OR Metathesis as Proposed by Hasanayn

Figure S12 shows the same pathway for ester hydrogenolysis shown in Figure 9, except that two ethanol molecules have been included instead of one or zero. With two ethanols, the overall barrier is 29.2 kcal/mol, compared with 27.5 kcal/mol with only one ethanol molecule.

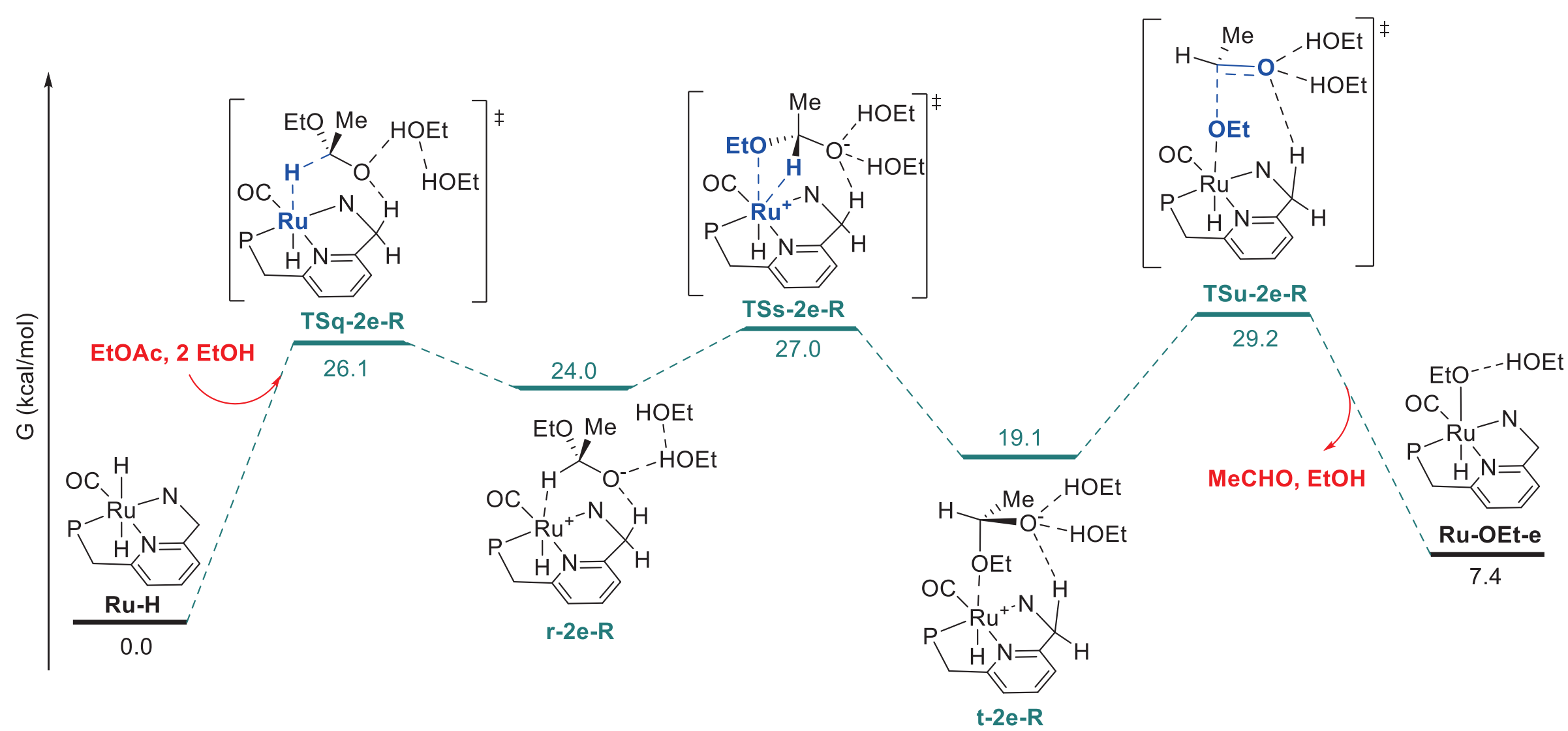


**Figure S12.** CH/OR metathesis pathway with two explicit ethanol molecules.

Figure S13 shows an analogous CH/OR metathesis pathway for ester hydrogenolysis, except that the configuration at the stereogenic carbon is S instead of R. The overall barrier of 29.0 is slightly higher than the barrier of 27.5 for the diastereomeric pathway shown in Figure 9.

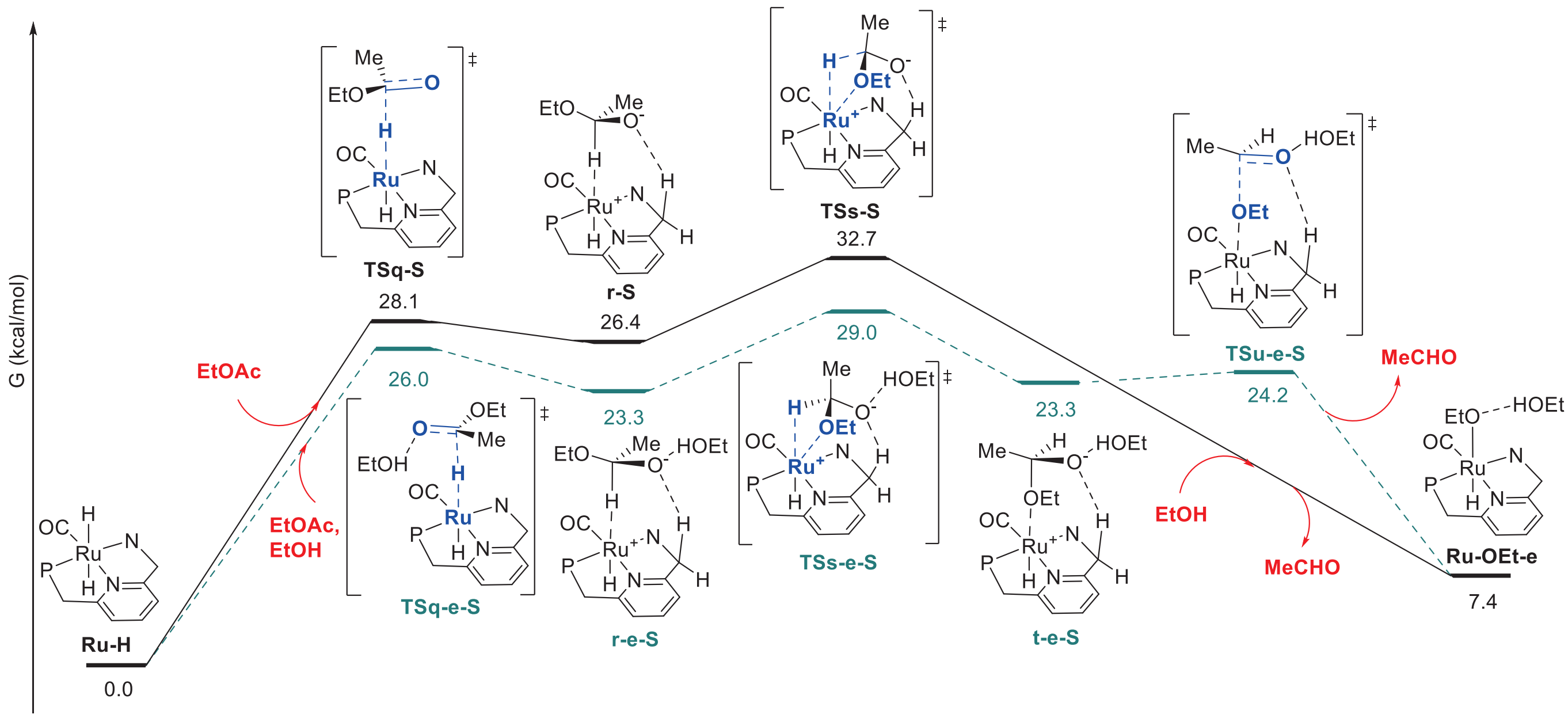


**Figure S13.** CH/OR metathesis pathway with a different configuration at carbon.

### Alternative Pathways for Ester Hydrogenolysis Involving Dearomatization of the Pincer Ligand

Figure S14 shows an alternative pathway for ester hydrogenolysis, which is the same as that shown in Figure 10 except that the configuration at the stereogenic carbon is S instead of R. This pathway has an overall barrier of 28.1 kcal/mol, compared with 28.0 for the pathway in Figure 10.

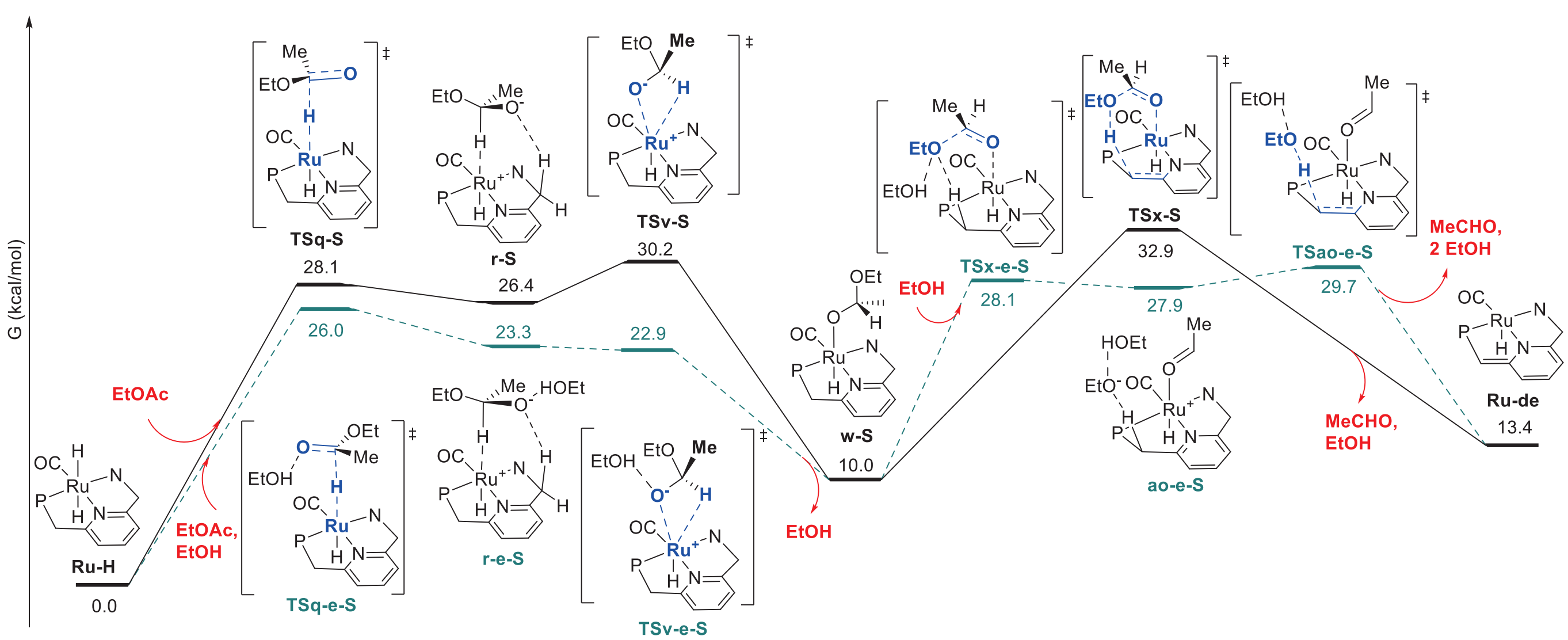


**Figure S14.** Metal-ligand-cooperative pathway for ester hydrogenolysis with a different configuration at carbon.

Although it is possible to identify mechanisms where the cleavage of the C-O bond is promoted by proton transfer from the ligand $CH_2$ linkers as in **TSx-S** above, it is also possible with one explicit ethanol molecule for C-O cleavage to occur without deprotonation of the pincer ligand. In this case, the aldehyde can dissociate and **Ru-OEt-e** can be regenerated without any need for ligand dearomatization. Figure S15 shows this mechanism, which has a slightly lower barrier than the path in Figure S14.

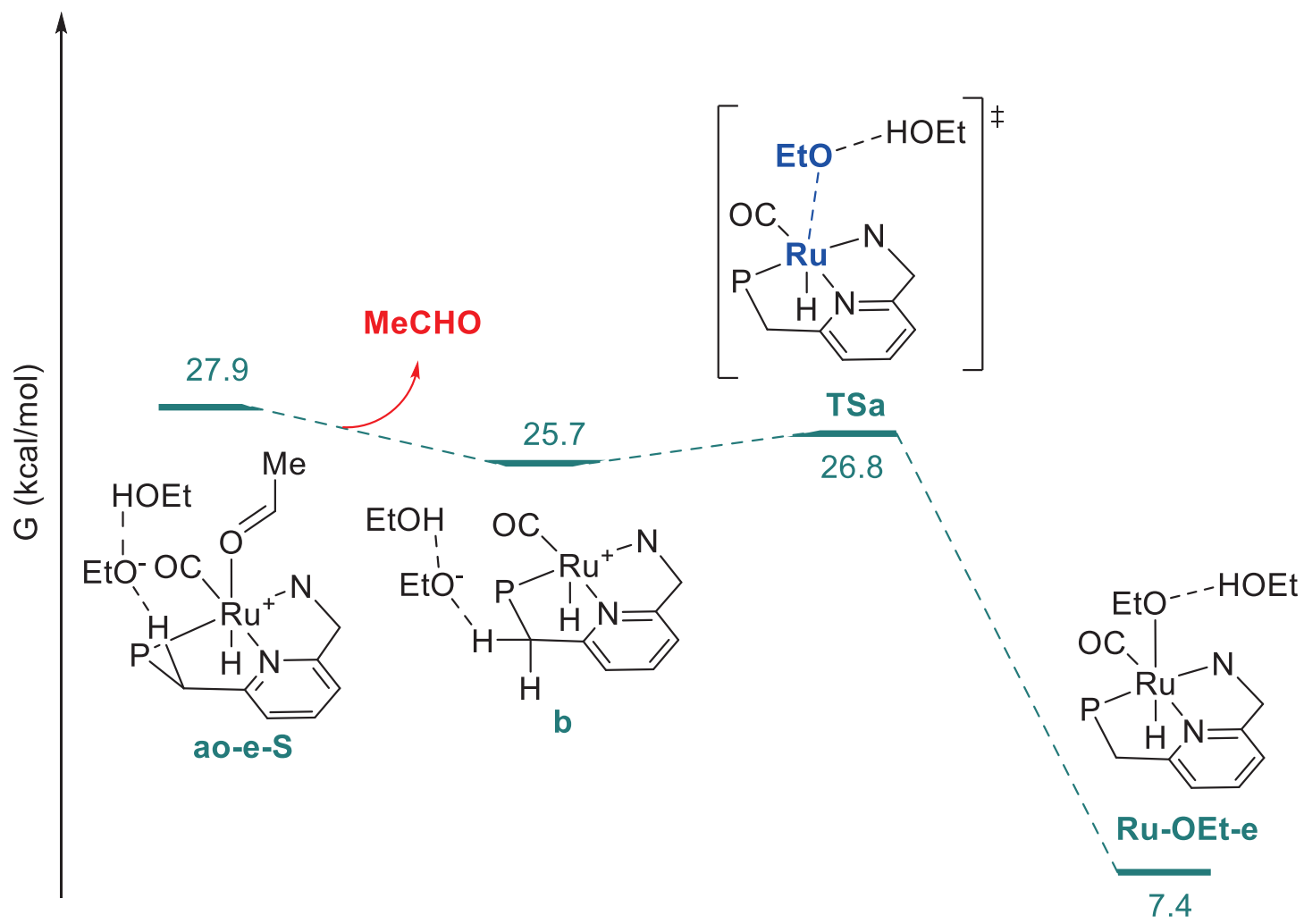


**Figure S15.** Alternative mechanism to regenerate **Ru-OEt-e** from **TSx-S** without ligand dearomatization.

Figure S16 shows an alternative pathway to generate the O-bound hemiacetaloxide complex **w-S** from the where the anionic oxygen is initially protonated through **TSap-S** to release the free hemiacetal, which then rebinds through the hydroxyl group. Deprotonation of the hydroxyl group by the pincer ligand through **TSar-S** then generates **w-S**. This pathway has a higher barrier of 30.4 kcal/mol compared with the barrier of 26.3 kcal/mol connecting **r-e-R** and **w-R** in Figure 10.

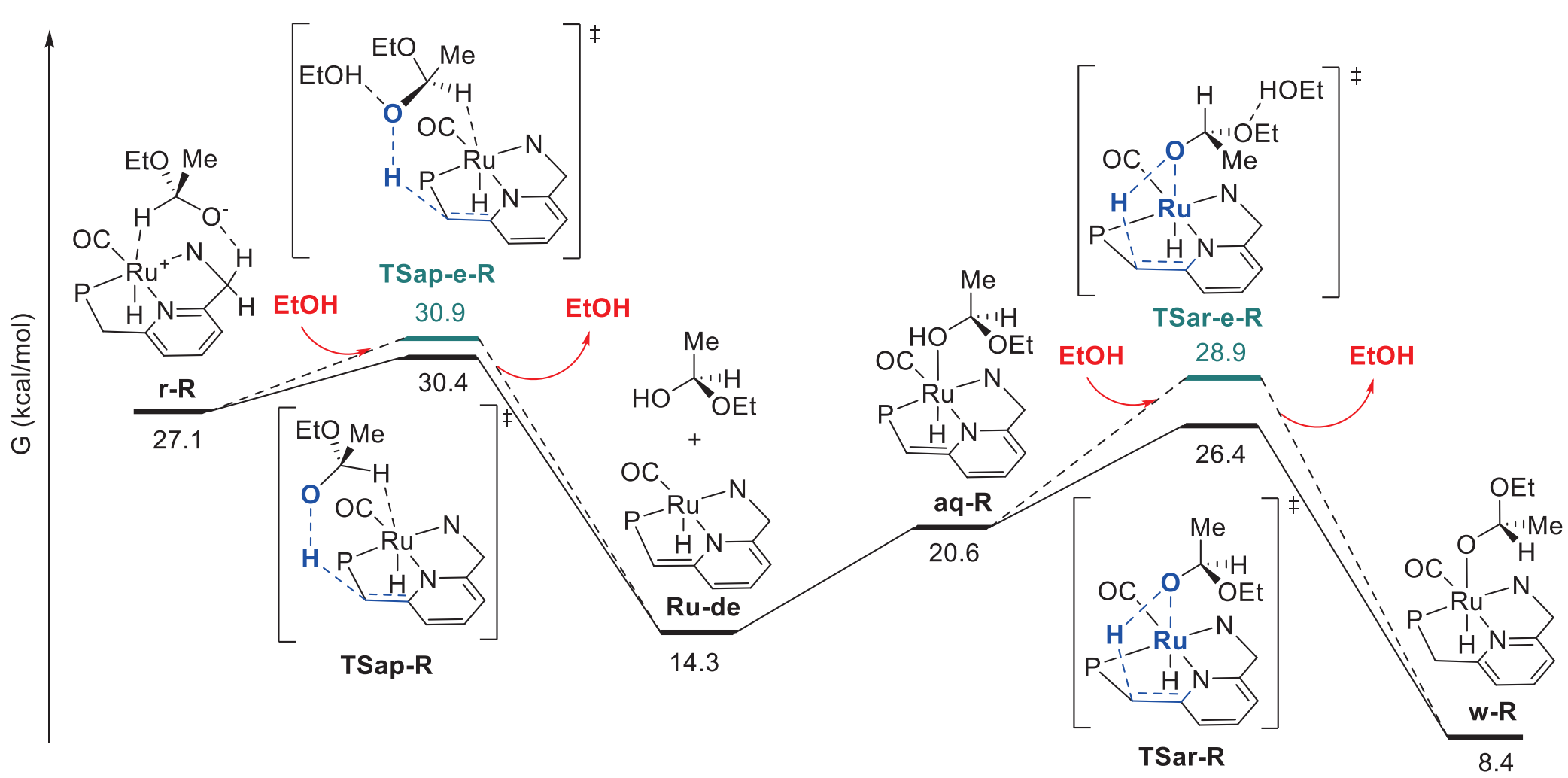


**Figure S16.** Protonation of oxygen to initially release the free hemiacetal, R configuration at carbon.

Figure S17 shows a diastereomeric mechanism for formation of a ruthenium-hemiacetaloxide complex, where the configuration at carbon is S instead of R as in Figure S16. This pathway has an overall barrier of 29.5 kcal/mol, higher than the barrier of 26.3 kcal/mol in Figure 10.

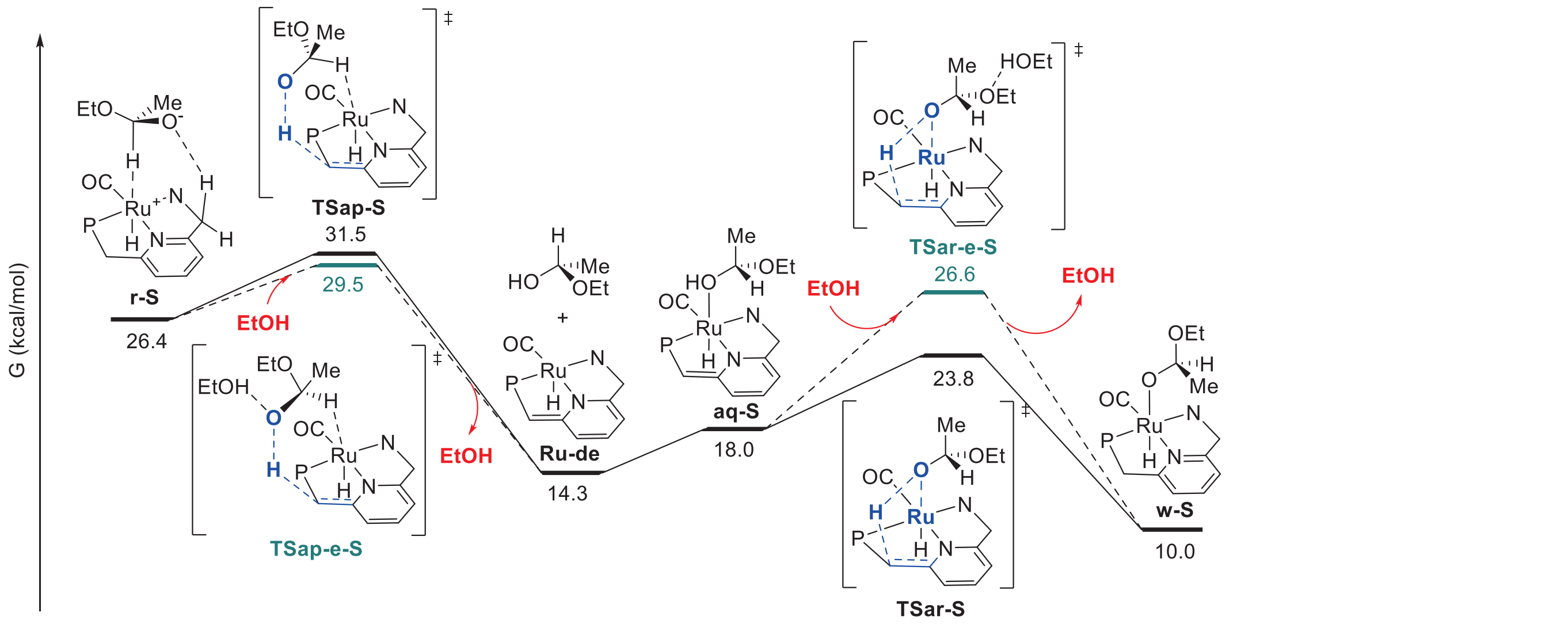


**Figure S17.** Protonation of oxygen to initially release the free hemiacetal, S configuration at carbon.

Figure S18 shows an alternative metal-ligand-cooperative mechanism for C-O cleavage, where the hemiacetal oxygen is deprotonated by the pincer ligand to release acetaldehyde. This pathway has a higher barrier of 29.5 kcal/mol compared to the barrier of 28.0 kcal/mol for C-O cleavage through **TSx-e-R** in Figure 10. Note that without explicit ethanol, C-O cleavage is concerted with proton transfer in **TSat-R**, while this process is stepwise with explicit ethanol.

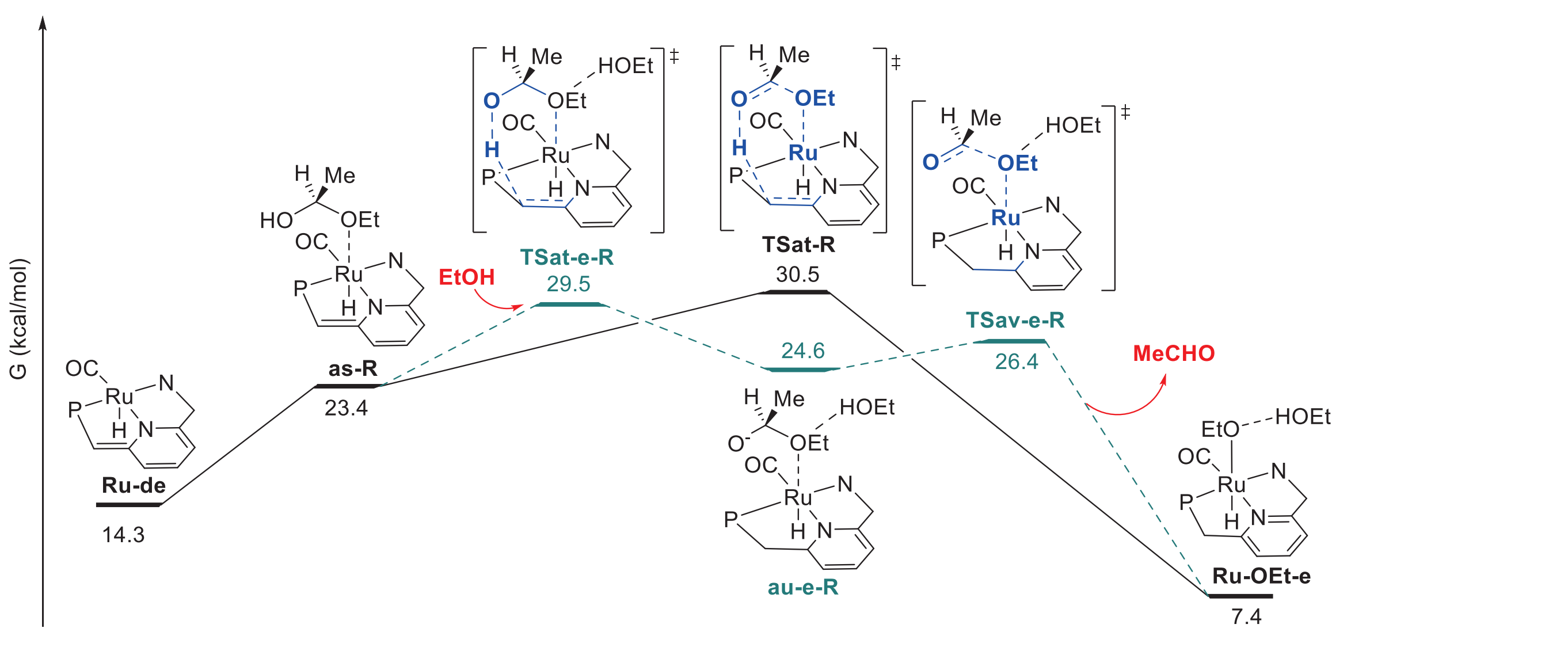


**Figure S18.** C-O cleavage by deprotonation of the hemiacetal to release acetaldehyde, R configuration at carbon.

Figure S19 shows a diastereomeric mechanism vs. that in Figure S18 for the release of the aldehyde, where the configuration at the hemiacetal carbon is S instead of R. This pathway has a higher barrier of 28.3 kcal/mol compared to the barrier of 28.0 kcal/mol for C-O cleavage through **TSx-e-R** in Figure 10. Note that without explicit ethanol, C-O cleavage is concerted with proton transfer in **TSat-S**, while this process is stepwise with explicit ethanol.

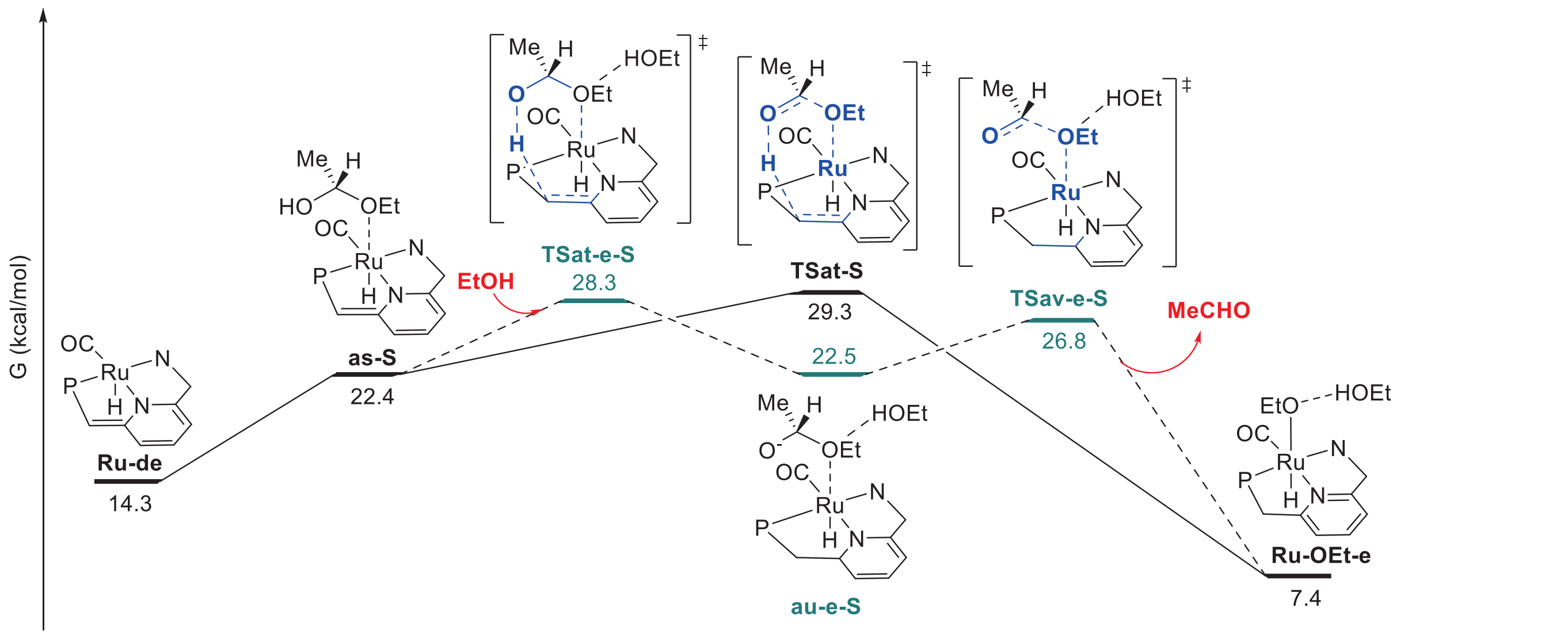


**Figure S19.** C-O cleavage by deprotonation of the hemiacetal to release acetaldehyde, S configuration at carbon.

Figure S20 shows a pathway for C-O cleavage involving the $NCH_2$ linker instead of the $PCH_2$ linker as shown in Figure 10. The overall barrier of 35.1 kcal/mol is higher here compared to 28.0 in the pathway in Figure 10.

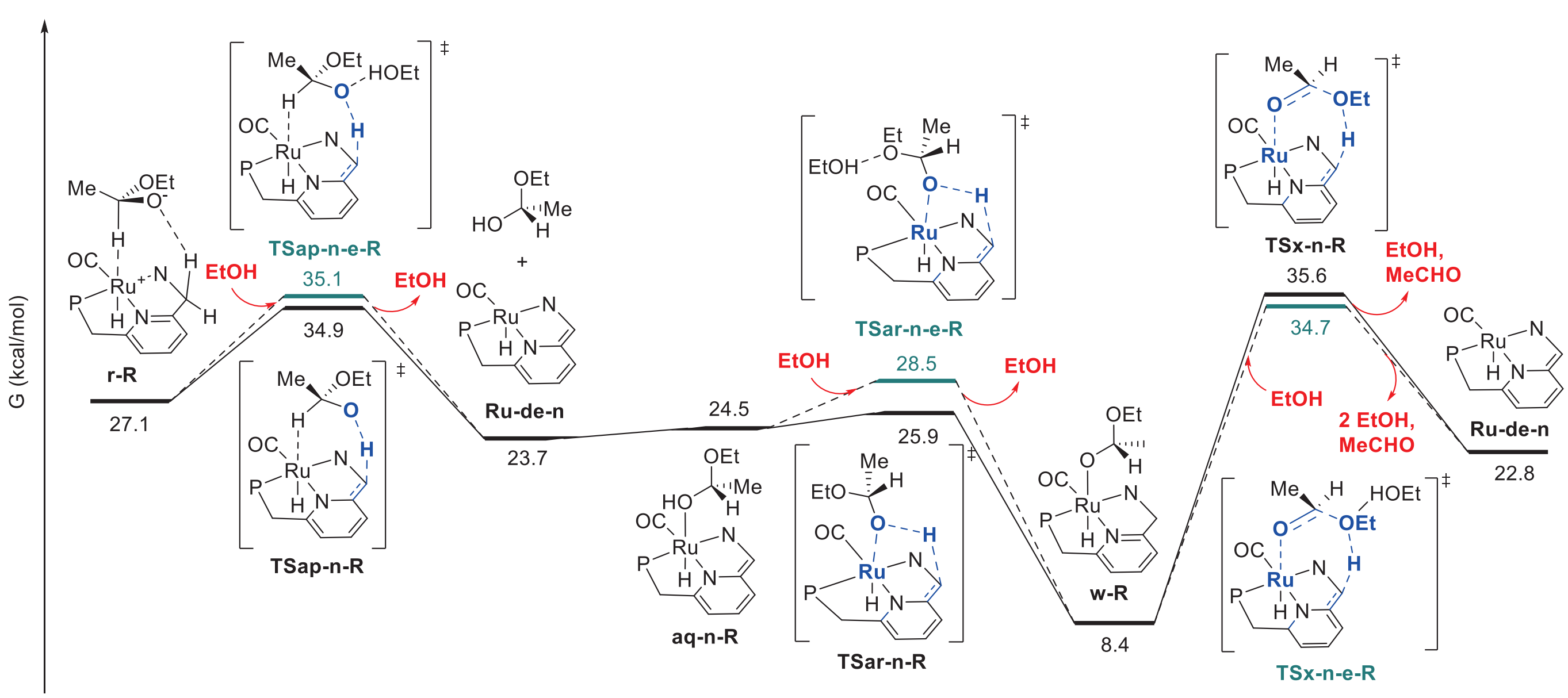


**Figure S20.** C-O cleavage via protonation of the ethoxy group by the pincer $NCH_2$ linker, R configuration at carbon.

Figure S21 shows a diastereomeric pathway to that in Figure S20, where the hemiacetal has an S configuration instead of R. The overall barrier of 34.8 kcal/mol is higher here compared to 28.0 in the pathway in Figure 10.

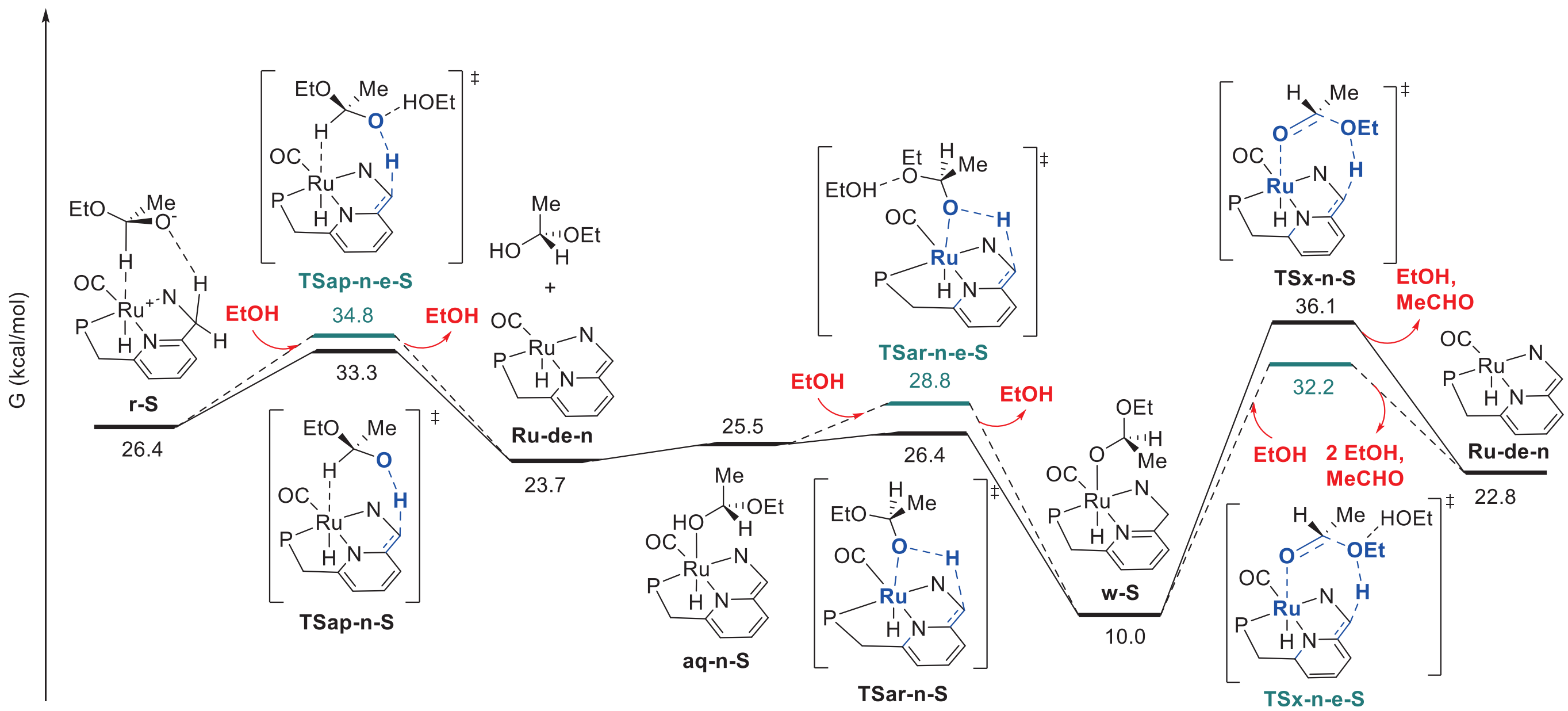


**Figure S21.** C-O cleavage via protonation of the ethoxy group by the pincer $NCH_2$ linker, S configuration at carbon.

Figure S22 shows a pathway for C-O cleavage where the pincer ligand linker adjacent to nitrogen deprotonates the hemiacetal oxygen, resulting in release of the aldehyde. This pathway has an overall barrier of 31.8 kcal/mol compared to 28.0 in the pathway in Figure 10.

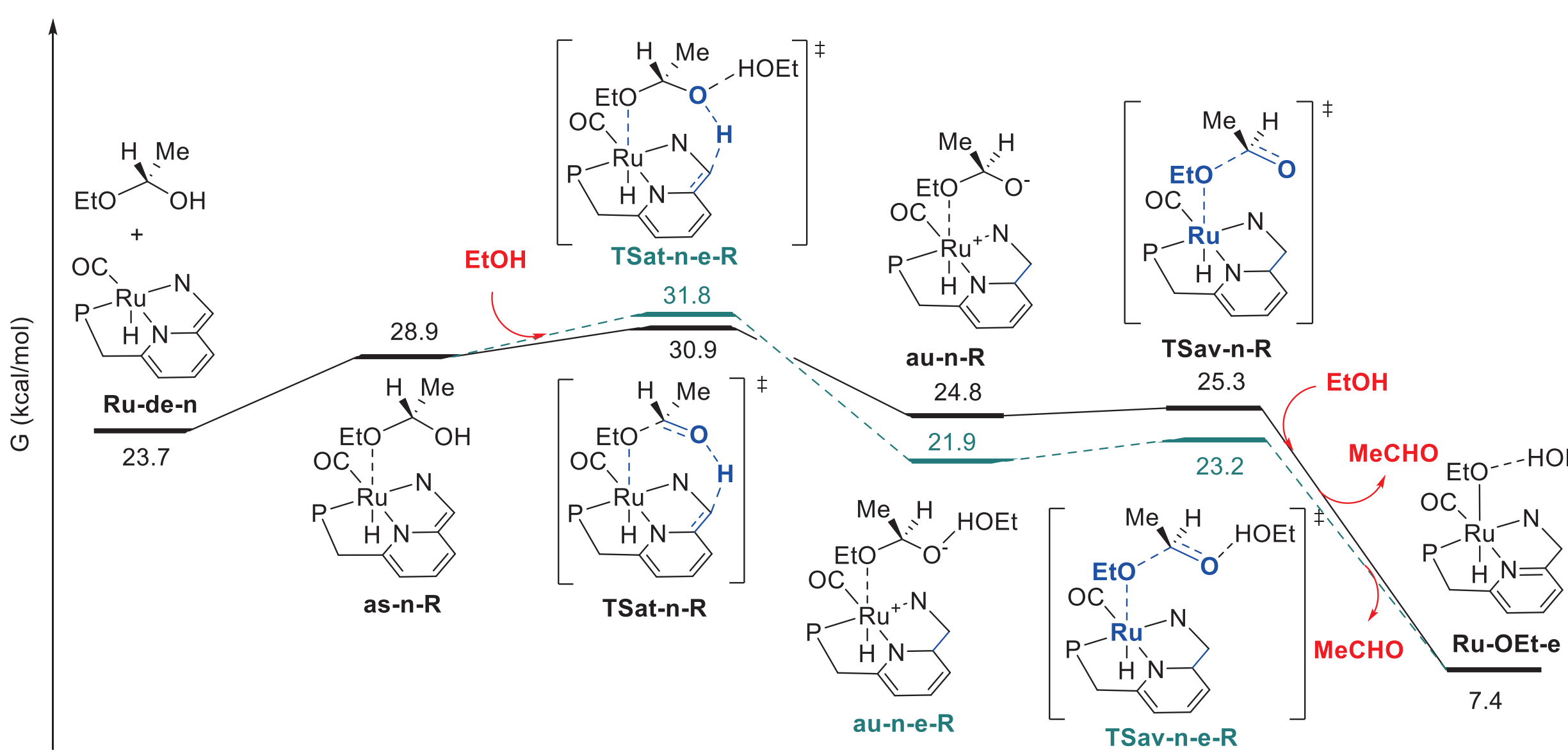


**Figure S22.** C-O cleavage via deprotonation of the hemiacetal to release acetaldehyde, R configuration at carbon.

Figure S23 shows a diastereomeric pathway to that in Figure S22, where the hemiacetal has an S configuration instead of R. This pathway has an overall barrier of 33.0 kcal/mol compared to 28.0 in the pathway in Figure 10.

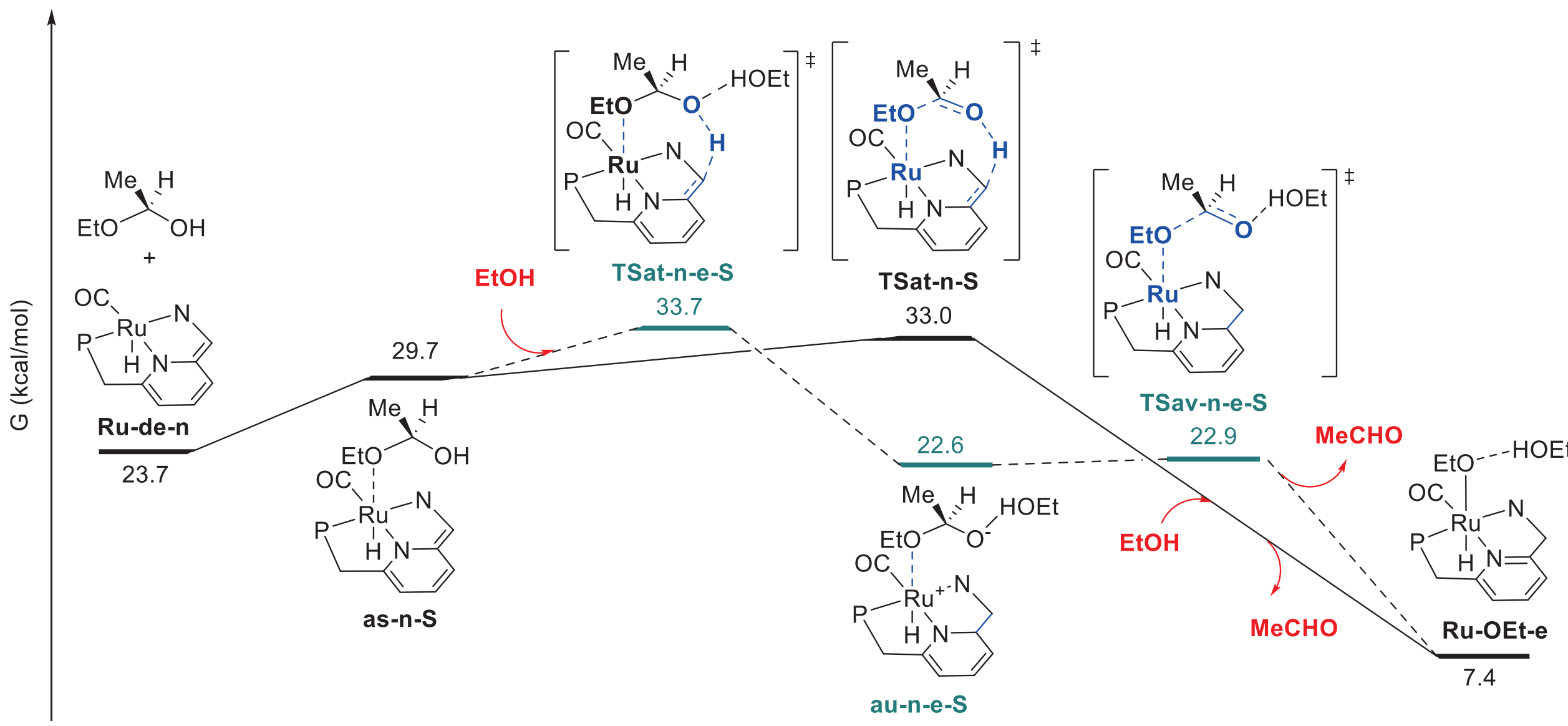


**Figure S23.** C-O cleavage via deprotonation of the hemiacetal to release acetaldehyde, S configuration at carbon.

### Alternative Pathways for Aldehyde Hydrogenation

Figure S24 shows a variation of the MEP shown in Figure 11, where the explicit ethanol molecule is not included. Without explicit ethanol, the barrier increases from 19.4 kcal/mol (Figure 11) to 28.0 kcal/mol.

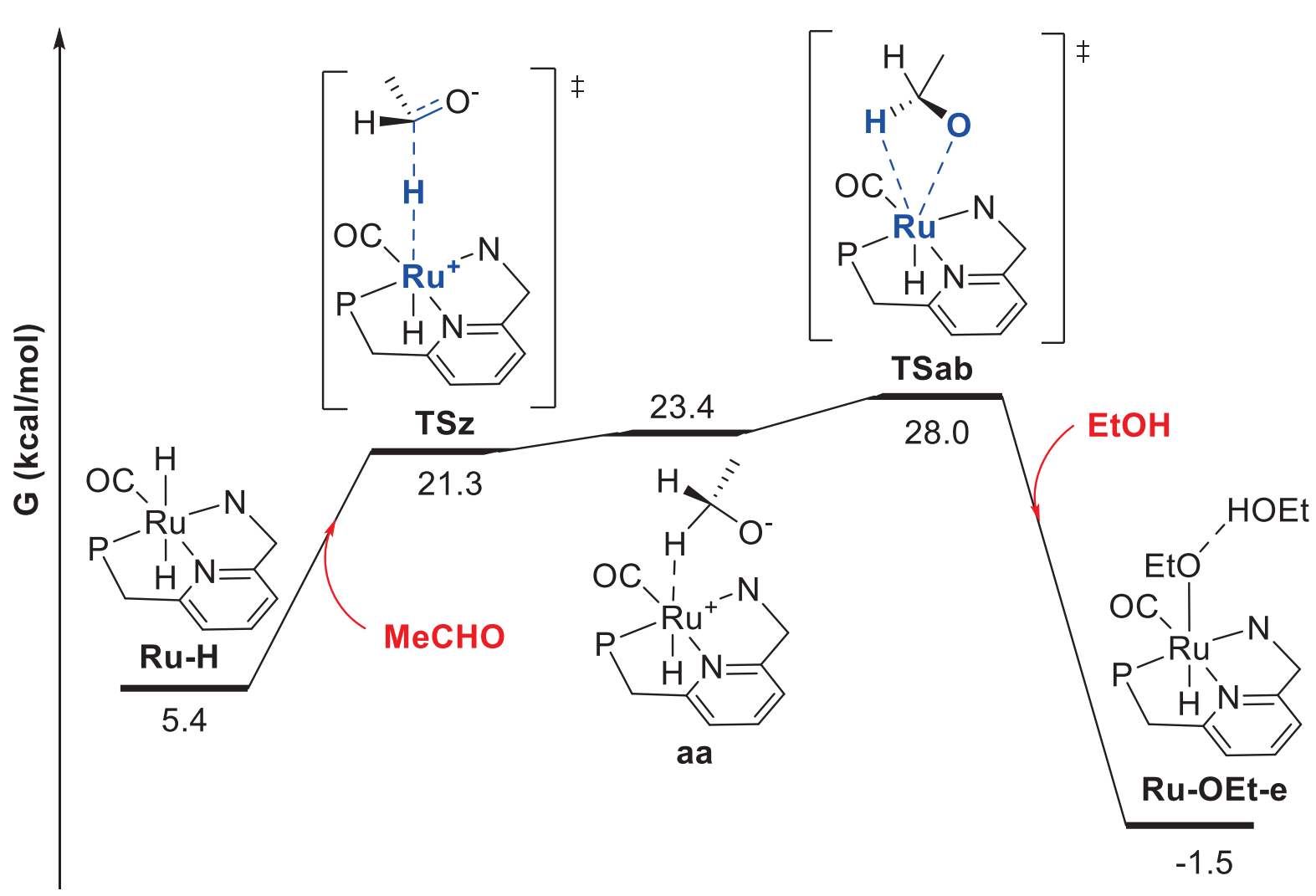


**Figure S24.** Aldehyde hydrogenation without explicit ethanol.

Figure S25 shows an alternative pathway for aldehyde hydrogenation involving dechelation of the $NEt_2$ group followed by inner-sphere 1,2-insertion of the C=O bond into Ru-H. This pathway has a higher barrier of 29.4 kcal/mol compared with the barrier of 19.4 kcal/mol for the MEP shown in Figure 11.

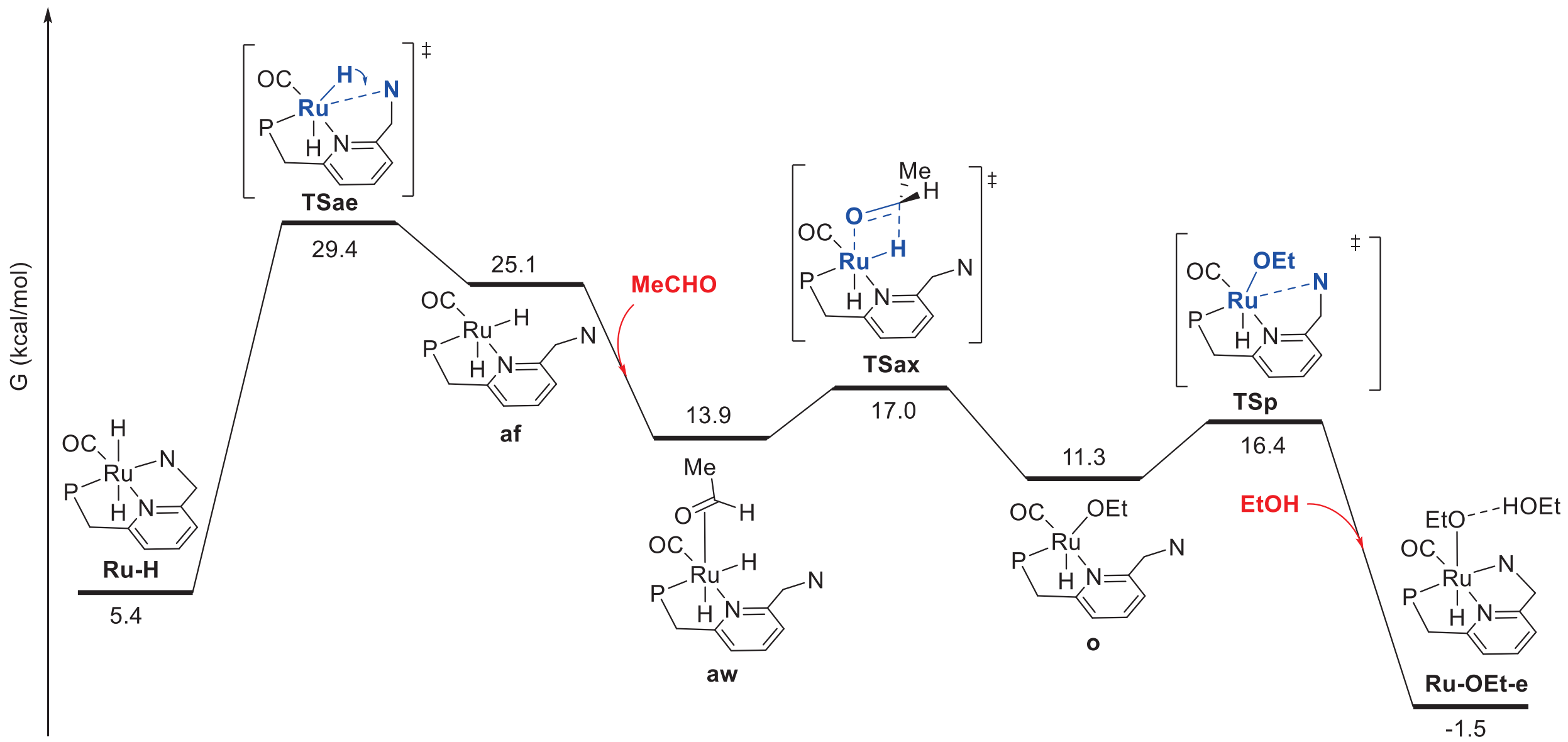


**Figure S25.** Aldehyde hydrogenation via dechelation and inner-sphere insertion, O trans to the hydride.

Figure S26 shows a pathway for aldehyde hydrogenation analogous to that shown in Figure S25, with a different configuration at ruthenium. Here, the aldehyde oxygen is trans to phosphorus rather than the ruthenium hydride. This pathway has a higher barrier of 31.6 compared with the barrier of 19.4 kcal/mol for the MEP shown in Figure 11.

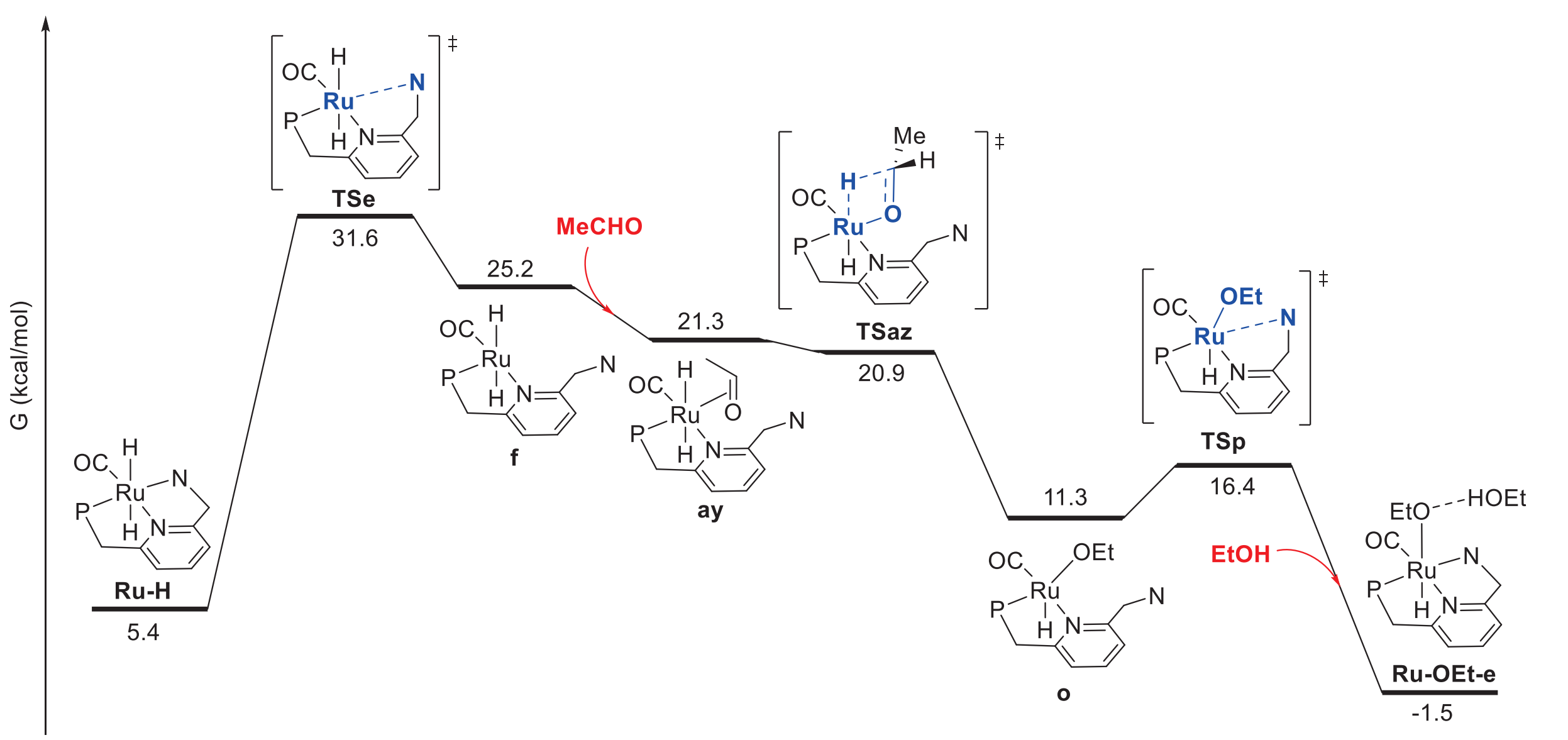


**Figure S26.** Aldehyde hydrogenation via dechelation and inner-sphere insertion, O trans to P.

Figure S27 shows a pathway for aldehyde hydrogenation where hydride attack through **TSz** as shown in the MEP in Figure 11 is followed by proton transfer from the ligand $PCH_2$ linker to the ethoxide oxygen. This pathway has a higher barrier of 23.6 kcal/mol compared to 19.4 in the MEP in Figure 11.

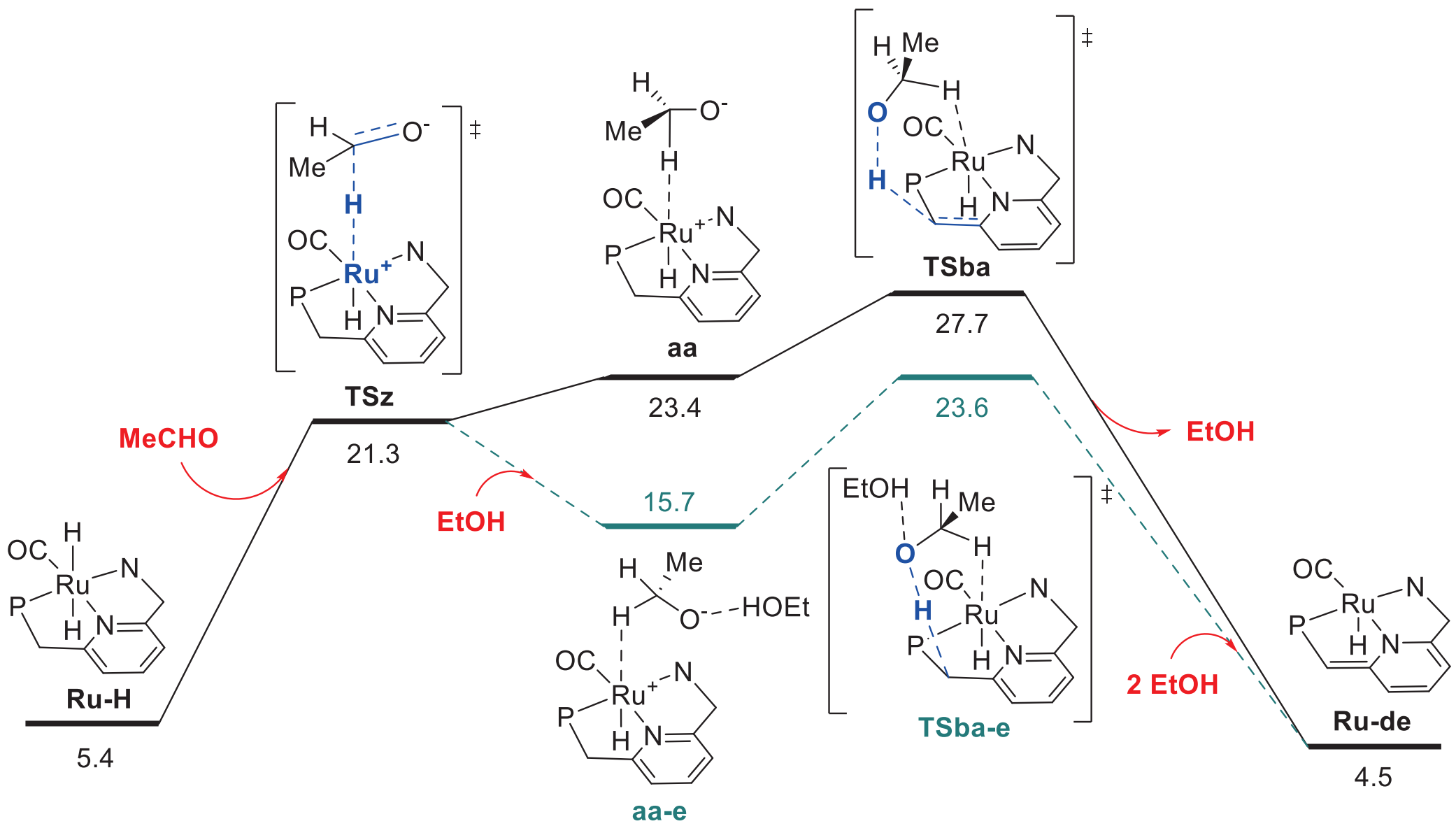


**Figure S27**. Aldehyde hydrogenation by the bifunctional double hydrogen transfer mechanism.

**Recalculation of the Energetic Span using Hexyl Hexanoate**

Because we used the ester hexyl hexanoate in our experimental studies and we used the truncated model ethyl acetate in our DFT calculations, we considered the possibility that this truncation could lead to significant differences in the calculated free energies. To assess this possibility, we recalculated the energetic span for the rate-determining ester hydrogenolysis sequence (**Ru-H** to **TSh-S**, see Figure 8 in the main text). As Figure S28 below shows, this energy difference is calculated to be 26.0 kcal/mol with hexyl hexanoate as the ester. This value is very close to the calculated energetic span of 26.5 kcal/mol using ethyl acetate as the substrate.

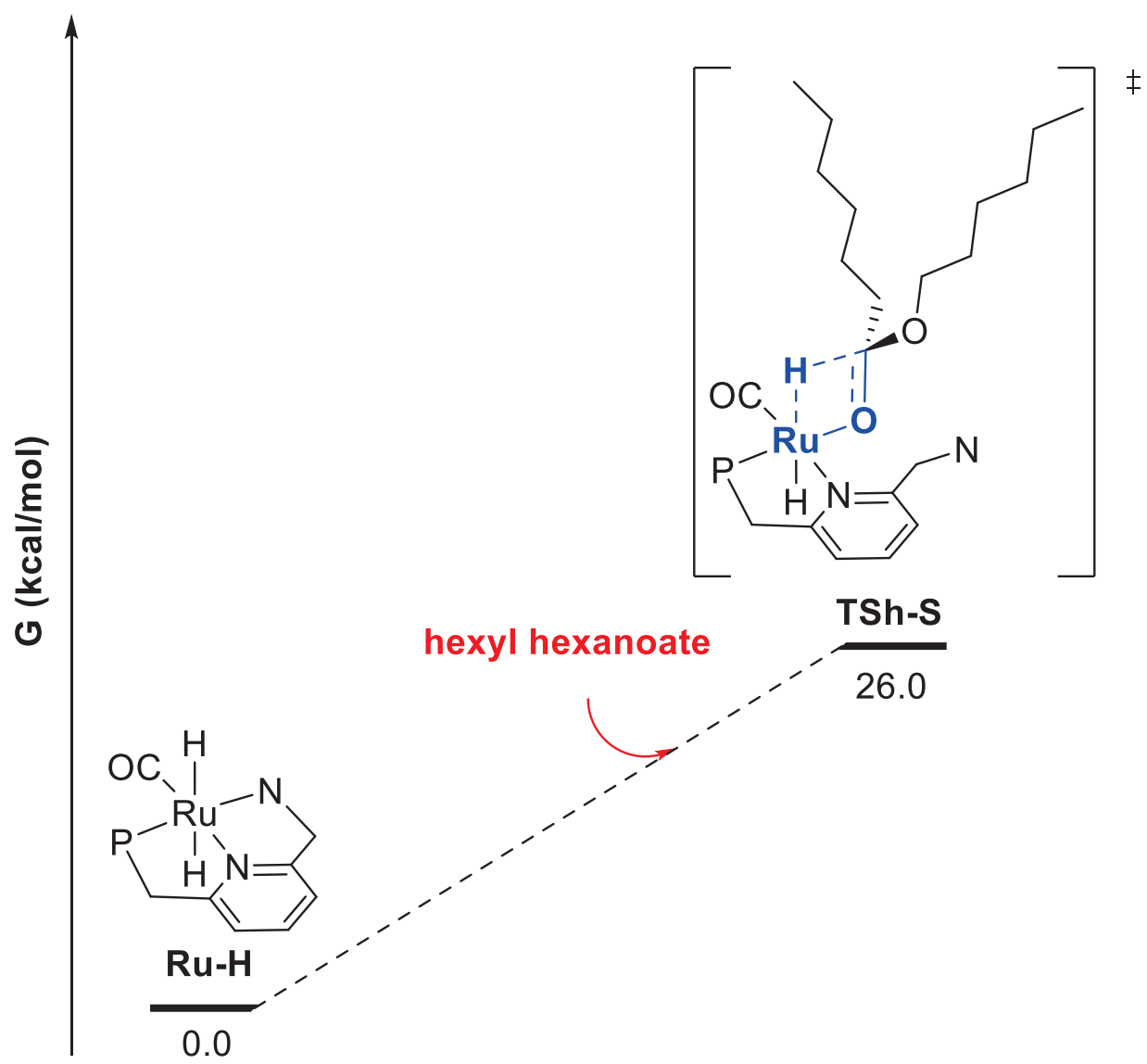


**Figure S28**. The calculated energetic span using the experimental ester hexyl hexanoate.

**Variation of the Specific Minimum-Energy Pathway by Functional Within Each Class of Mechanism**

Because multiple diastereomeric pathways and other alternatives were considered for each class of mechanism as shown above, the specific MEP varies depending on the functional. Below, we list the specific MEP for each class of mechanism with each functional. The overall barriers for these paths are presented in the main text in Table 1.

**Hydrogen activation**

Ethoxide pathways using ωB97M-V
Minimum-energy Path: Ru-OEt-e (1.98) -> TSa (21.42) -> b (20.31) -> c (19.20) -> TSd (20.15) -> Ru-H (0.00)
Highest Energy Peak on Path: 21.42 kcal/mol

Ethoxide pathways without explicit ethanol using ωB97M-V
Minimum-energy Path: Ru-OEt-e (1.98) -> b-e (34.62) -> c-e (35.07) -> Ru-H (0.00)
Highest Energy Peak on Path: 35.07 kcal/mol

Dearomatization pathways using ωB97M-V
Minimum-energy Path: Ru-OEt-e (1.98) -> ac-N (22.42) -> TSad-N (27.88) -> Ru-H (0.00)

Highest Energy Peak on Path: 27.88 kcal/mol

Dearomatization pathways without explicit ethanol using ωB97M-V
Minimum-energy Path: Ru-OEt-e (1.98) -> ac-N (22.42) -> TSad-N (27.88) -> Ru-H (0.00)
Highest Energy Peak on Path: 27.88 kcal/mol

Ethoxide pathways using M06L
Minimum-energy Path: Ru-OEt-e (1.75) -> TSa (20.87) -> b (19.82) -> c (20.08) -> TSd (21.59) -> Ru-H (0.00)
Highest Energy Peak on Path: 21.59 kcal/mol

Ethoxide pathways without explicit ethanol using M06L
Minimum-energy Path: Ru-OEt-e (1.75) -> b-e (30.54) -> c-e (32.31) -> Ru-H (0.00)
Highest Energy Peak on Path: 32.31 kcal/mol

Dearomatization pathways using M06L
Minimum-energy Path: Ru-OEt-e (1.75) -> ac-N (20.59) -> TSad-N (23.19) -> Ru-H (0.00)
Highest Energy Peak on Path: 23.19 kcal/mol

Dearomatization pathways without explicit ethanol using M06L
Minimum-energy Path: Ru-OEt-e (1.75) -> ac-N (20.59) -> TSad-N (23.19) -> Ru-H (0.00)
Highest Energy Peak on Path: 23.19 kcal/mol

Ethoxide pathways using MN15L
Minimum-energy Path: Ru-OEt-e (-0.66) -> TSa (18.75) -> b (17.45) -> c (22.68) -> TSd (24.00) -> Ru-H (0.00)
Highest Energy Peak on Path: 24.0 kcal/mol

Ethoxide pathways without explicit ethanol using MN15L
Minimum-energy Path: Ru-OEt-e (-0.66) -> b-e (31.90) -> c-e (35.93) -> Ru-H (0.00)
Highest Energy Peak on Path: 35.93 kcal/mol

Dearomatization pathways using MN15L
Minimum-energy Path: Ru-OEt-e (-0.66) -> ac-N (23.09) -> TSad-N (26.00) -> Ru-H (0.00)
Highest Energy Peak on Path: 26.0 kcal/mol

Dearomatization pathways without explicit ethanol using MN15L
Minimum-energy Path: Ru-OEt-e (-0.66) -> ac-N (23.09) -> TSad-N (26.00) -> Ru-H (0.00)
Highest Energy Peak on Path: 26.0 kcal/mol

Ethoxide pathways using TPSS
Minimum-energy Path: Ru-OEt-e (13.33) -> TSa (29.89) -> b (28.32) -> c (28.76) -> TSd (30.03) -> Ru-H (0.00)
Highest Energy Peak on Path: 30.03 kcal/mol

Ethoxide pathways without explicit ethanol using TPSS
Minimum-energy Path: Ru-OEt-e (13.33) -> b-e (29.22) -> c-e (33.67) -> Ru-H (0.00)
Highest Energy Peak on Path: 33.67 kcal/mol

Dearomatization pathways using TPSS
Minimum-energy Path: Ru-OEt-e (13.33) -> ac-N (19.41) -> TSad-N (22.26) -> Ru-H (0.00)
Highest Energy Peak on Path: 22.26 kcal/mol

Dearomatization pathways without explicit ethanol using TPSS
Minimum-energy Path: Ru-OEt-e (13.33) -> ac-N (19.41) -> TSad-N (22.26) -> Ru-H (0.00)
Highest Energy Peak on Path: 22.26 kcal/mol

Ethoxide pathways using TPSS-D4
Minimum-energy Path: Ru-OEt-e (-1.23) -> TSa (17.35) -> b (16.17) -> c (18.15) -> TSd (19.43) -> Ru-H (0.00)
Highest Energy Peak on Path: 19.43 kcal/mol

Ethoxide pathways without explicit ethanol using TPSS-D4
Minimum-energy Path: Ru-OEt-e (-1.23) -> b-e (27.18) -> c-e (31.04) -> Ru-H (0.00)
Highest Energy Peak on Path: 31.04 kcal/mol

Dearomatization pathways using TPSS-D4
Minimum-energy Path: Ru-OEt-e (-1.23) -> ac-N (18.69) -> TSad-N (19.88) -> Ru-H (0.00)
Highest Energy Peak on Path: 19.88 kcal/mol

Dearomatization pathways without explicit ethanol using TPSS-D4
Minimum-energy Path: Ru-OEt-e (-1.23) -> ac-N (18.69) -> TSad-N (19.88) -> Ru-H (0.00)
Highest Energy Peak on Path: 19.88 kcal/mol

Ethoxide pathways using M06
Minimum-energy Path: Ru-OEt-e (3.02) -> TSa (19.87) -> b (18.66) -> c (20.28) -> TSd (21.59) -> Ru-H (0.00)
Highest Energy Peak on Path: 21.59 kcal/mol

Ethoxide pathways without explicit ethanol using M06
Minimum-energy Path: Ru-OEt-e (3.02) -> b-e (30.50) -> c-e (33.52) -> Ru-H (0.00)
Highest Energy Peak on Path: 33.52 kcal/mol

Dearomatization pathways using M06
Minimum-energy Path: Ru-OEt-e (3.02) -> ac-N (18.40) -> TSad-N (23.51) -> Ru-H (0.00)
Highest Energy Peak on Path: 23.51 kcal/mol

Dearomatization pathways without explicit ethanol using M06
Minimum-energy Path: Ru-OEt-e (3.02) -> ac-N (18.40) -> TSad-N (23.51) -> Ru-H (0.00)
Highest Energy Peak on Path: 23.51 kcal/mol

Ethoxide pathways using B3LYP-D3
Minimum-energy Path: Ru-OEt-e (-0.32) -> TSa (10.69) -> b (9.54) -> c (15.51) -> TSd (16.64) -> Ru-H (0.00)
Highest Energy Peak on Path: 16.64 kcal/mol

Ethoxide pathways without explicit ethanol using B3LYP-D3
Minimum-energy Path: Ru-OEt-e (-0.32) -> b-e (23.48) -> c-e (30.88) -> Ru-H (0.00)
Highest Energy Peak on Path: 30.88 kcal/mol

Dearomatization pathways using B3LYP-D3
Minimum-energy Path: Ru-OEt-e (-0.32) -> ac-N (18.75) -> TSad-N (22.83) -> Ru-H (0.00)
Highest Energy Peak on Path: 22.83 kcal/mol

Dearomatization pathways without explicit ethanol using B3LYP-D3
Minimum-energy Path: Ru-OEt-e (-0.32) -> ac-N (18.75) -> TSad-N (22.83) -> Ru-H (0.00)

Highest Energy Peak on Path: 22.83 kcal/mol

**Ester Hydrogenolysis**

Dechelation pathways using wB97M-V
Minimum-energy Path: Ru-H (0.00) -> TSe (26.21) -> f (19.81) -> g (21.71) -> TSh-S (26.47) -> i-S (22.77) -> TSj-e-S (25.92) -> k-S (21.71) -> TSl-S (25.34) -> m-S (19.01) -> TSn-S (25.01) -> o (20.17) -> TSp (25.28) -> Ru-OEt-e (7.39)
Highest Energy Peak on Path: 26.47 kcal/mol

Dechelation pathways without explicit ethanol using wB97M-V
Minimum-energy Path: Ru-H (0.00) -> TSe (26.21) -> f (19.81) -> g (21.71) -> TSh-S (26.47) -> i-S (22.77) -> TSj-S (26.48) -> k-S (21.71) -> TSl-S (25.34) -> m-S (19.01) -> TSn-S (25.01) -> o (20.17) -> TSp (25.28) -> Ru-OEt-e (7.39)
Highest Energy Peak on Path: 26.48 kcal/mol

CH/OR metathesis pathways using wB97M-V
Minimum-energy Path: Ru-H (0.00) -> TSq-e-R (23.76) -> r-e-R (22.88) -> TSs-e-R (27.55) -> t-e-R (23.69) -> TSu-e-R (26.48) -> Ru-OEt-e (7.39)
Highest Energy Peak on Path: 27.55 kcal/mol

CH/OR metathesis pathways without explicit ethanol using wB97M-V
Minimum-energy Path: Ru-H (0.00) -> TSq-S (28.09) -> r-S (26.42) -> TSs-S (32.71) -> Ru-OEt-e (7.39)
Highest Energy Peak on Path: 32.71 kcal/mol

Dearomatization pathways using wB97M-V
Minimum-energy Path: Ru-H (0.00) -> TSq-e-R (23.76) -> r-e-R (22.88) -> TSv-e-R (26.32) -> w-R (8.44) -> TSx-e-R (28.03) -> Ru-de (13.40) -> Ru-OEt-e (7.39)
Highest Energy Peak on Path: 28.03 kcal/mol

Dearomatization pathways without explicit ethanol using wB97M-V
Minimum-energy Path: Ru-H (0.00) -> TSq-S (28.09) -> r-S (26.42) -> TSv-S (30.15) -> w-S (10.02) -> TSar-S (23.81) -> aq-S (18.03) -> Ru-de-S (13.40) -> as-S (22.36) -> TSat-S (29.29) -> Ru-OEt-e (7.39)
Highest Energy Peak on Path: 30.15 kcal/mol

Dechelation pathways using M06L
Minimum-energy Path: Ru-H (0.00) -> TSae (18.12) -> af (16.57) -> ag-R (12.11) -> TSah-R (25.32) -> ai-R (23.84) -> TSaj-e-S (27.56) -> k-R (20.21) -> ak-R (18.43) -> TSal-R (27.39) -> o (13.77) -> TSp (16.68) -> Ru-OEt-e (6.65)
Highest Energy Peak on Path: 27.56 kcal/mol

Dechelation pathways without explicit ethanol using M06L
Minimum-energy Path: Ru-H (0.00) -> TSe (22.42) -> f (16.48) -> g (19.45) -> TSh-S (27.58) -> i-S (22.60) -> TSj-S (26.60) -> k-S (21.16) -> ak-S (19.57) -> TSal-S (24.22) -> o (13.77) -> TSp (16.68) -> Ru-OEt-e (6.65)
Highest Energy Peak on Path: 27.58 kcal/mol

CH/OR metathesis pathways using M06L
Minimum-energy Path: Ru-H (0.00) -> TSq-e-R (24.51) -> r-e-R (22.80) -> TSs-e-R (29.11) -> t-e-R (25.87) -> TSu-e-R (25.34) -> Ru-OEt-e (6.65)
Highest Energy Peak on Path: 29.11 kcal/mol

CH/OR metathesis pathways without explicit ethanol using M06L
Minimum-energy Path: Ru-H (0.00) -> TSq-R (28.53) -> r-R (25.85) -> TSs-R (31.34) -> Ru-OEt-e (6.65)
Highest Energy Peak on Path: 31.34 kcal/mol

Dearomatization pathways using M06L
Minimum-energy Path: Ru-H (0.00) -> TSq-e-S (25.67) -> r-e-S (24.64) -> TSv-e-S (28.09) -> w-S (12.11) -> TSx-e-S (27.25) -> ao-e-S (26.57) -> TSao-e-S (28.25) -> Ru-de (11.03) -> Ru-OEt-e (6.65)
Highest Energy Peak on Path: 28.25 kcal/mol

Dearomatization pathways without explicit ethanol using M06L
Minimum-energy Path: Ru-H (0.00) -> TSq-R (28.53) -> r-R (25.85) -> TSap-R (31.85) -> Ru-de-R (11.03) -> aq-R (23.97) -> TSar-R (30.07) -> w-R (11.08) -> TSx-R (27.06) -> Ru-de (11.03) -> Ru-OEt-e (6.65)
Highest Energy Peak on Path: 31.85 kcal/mol

Dechelation pathways using MN15L
Minimum-energy Path: Ru-H (0.00) -> TSe (24.81) -> f (19.38) -> g (20.42) -> TSh-e-S (23.68) -> i-S (22.37) -> TSj-e-S (24.95) -> k-S (25.11) -> TSl-S (24.16) -> m-S (21.72) -> TSan-e-S (21.37) -> o (22.12) -> TSp (22.71) -> Ru-OEt-e (9.87)
Highest Energy Peak on Path: 25.11 kcal/mol

Dechelation pathways without explicit ethanol using MN15L
Minimum-energy Path: Ru-H (0.00) -> TSe (24.81) -> f (19.38) -> g (20.42) -> TSh-S (25.25) -> i-S (22.37) -> TSj-S (27.81) -> k-S (25.11) -> TSl-S (24.16) -> m-S (21.72) -> TSn-S (27.54) -> o (22.12) -> TSp (22.71) -> Ru-OEt-e (9.87)
Highest Energy Peak on Path: 27.81 kcal/mol

CH/OR metathesis pathways using MN15L
Minimum-energy Path: Ru-H (0.00) -> TSq-2e-R (24.15) -> r-2e-R (22.76) -> TSs-2r-R (26.09) -> t-2e-R (19.75) -> TSu-2e-R (26.65) -> Ru-OEt-e (9.87)
Highest Energy Peak on Path: 26.65 kcal/mol

CH/OR metathesis pathways without explicit ethanol using MN15L
Minimum-energy Path: Ru-H (0.00) -> TSq-S (25.77) -> r-S (26.55) -> TSs-S (31.40) -> Ru-OEt-e (9.87)
Highest Energy Peak on Path: 31.4 kcal/mol

Dearomatization pathways using MN15L
Minimum-energy Path: Ru-H (0.00) -> TSq-e-R (21.32) -> r-e-R (20.88) -> TSv-e-R (27.60) -> w-R (10.68) -> TSx-e-R (28.78) -> Ru-de (16.84) -> Ru-OEt-e (9.87)
Highest Energy Peak on Path: 28.78 kcal/mol

Dearomatization pathways without explicit ethanol using MN15L
Minimum-energy Path: Ru-H (0.00) -> TSq-R (27.00) -> r-R (25.96) -> TSap-R (31.33) -> Ru-de-R (16.84) -> aq-R (20.95) -> TSar-R (29.29) -> w-R (10.68) -> TSx-R (30.29) -> Ru-de (16.84) -> Ru-OEt-e (9.87)
Highest Energy Peak on Path: 31.33 kcal/mol

Dechelation pathways using TPSS
Minimum-energy Path: Ru-H (0.00) -> TSe (25.21) -> f (15.12) -> g (30.21) -> TSh-R (33.36) -> i-R (28.71) -> TSj-R (33.24) -> k-R (25.82) -> TSam-R (28.17) -> m-R (26.38) -> TSn-R (31.98) -> o (16.52) -> TSp (25.18) -> Ru-OEt-e (20.03)
Highest Energy Peak on Path: 33.36 kcal/mol

Dechelation pathways without explicit ethanol using TPSS
Minimum-energy Path: Ru-H (0.00) -> TSe (25.21) -> f (15.12) -> g (30.21) -> TSh-R (33.36) -> i-R (28.71) -> TSj-R (33.24) -> k-R (25.82) -> TSam-R (28.17) -> m-R (26.38) -> TSn-R (31.98) -> o (16.52) -> TSp (25.18) -> Ru-OEt-e (20.03)
Highest Energy Peak on Path: 33.36 kcal/mol

CH/OR metathesis pathways using TPSS
Minimum-energy Path: Ru-H (0.00) -> TSq-R (37.46) -> r-R (37.58) -> TSs-R (44.07) -> Ru-OEt-e (20.03)
Highest Energy Peak on Path: 44.07 kcal/mol

CH/OR metathesis pathways without explicit ethanol using TPSS
Minimum-energy Path: Ru-H (0.00) -> TSq-R (37.46) -> r-R (37.58) -> TSs-R (44.07) -> Ru-OEt-e (20.03)
Highest Energy Peak on Path: 44.07 kcal/mol

Dearomatization pathways using TPSS
Minimum-energy Path: Ru-H (0.00) -> TSq-R (37.46) -> r-R (37.58) -> TSap-R (42.17) -> Ru-de-R (9.52) -> aq-R (36.06) -> TSar-R (40.31) -> w-R (22.39) -> TSx-R (37.59) -> Ru-de (9.52) -> Ru-OEt-e (20.03)
Highest Energy Peak on Path: 42.17 kcal/mol

Dearomatization pathways without explicit ethanol using TPSS
Minimum-energy Path: Ru-H (0.00) -> TSq-R (37.46) -> r-R (37.58) -> TSap-R (42.17) -> Ru-de-R (9.52) -> aq-R (36.06) -> TSar-R (40.31) -> w-R (22.39) -> TSx-R (37.59) -> Ru-de (9.52) -> Ru-OEt-e (20.03)
Highest Energy Peak on Path: 42.17 kcal/mol

Dechelation pathways using TPSS-D4
Minimum-energy Path: Ru-H (0.00) -> TSe (27.53) -> f (20.04) -> g (24.34) -> TSh-S (29.31) -> i-S (24.04) -> TSj-S (28.56) -> k-S (22.30) -> ak-S (21.32) -> TSal-S (28.08) -> o (18.47) -> TSp (22.70) -> Ru-OEt-e (7.44)
Highest Energy Peak on Path: 29.31 kcal/mol

Dechelation pathways without explicit ethanol using TPSS-D4
Minimum-energy Path: Ru-H (0.00) -> TSe (27.53) -> f (20.04) -> g (24.34) -> TSh-S (29.31) -> i-S (24.04) -> TSj-S (28.56) -> k-S (22.30) -> ak-S (21.32) -> TSal-S (28.08) -> o (18.47) -> TSp (22.70) -> Ru-OEt-e (7.44)
Highest Energy Peak on Path: 29.31 kcal/mol

CH/OR metathesis pathways using TPSS-D4
Minimum-energy Path: Ru-H (0.00) -> TSq-e-R (19.87) -> r-e-R (20.47) -> TSs-e-R (28.70) -> t-e-R (25.29) -> TSu-e-R (25.23) -> Ru-OEt-e (7.44)
Highest Energy Peak on Path: 28.7 kcal/mol

CH/OR metathesis pathways without explicit ethanol using TPSS-D4
Minimum-energy Path: Ru-H (0.00) -> TSq-R (24.15) -> r-R (24.06) -> TSs-R (31.87) -> Ru-OEt-e (7.44)
Highest Energy Peak on Path: 31.87 kcal/mol

Dearomatization pathways using TPSS-D4
Minimum-energy Path: Ru-H (0.00) -> TSq-e-S (22.52) -> r-e-S (22.14) -> TSv-e-S (26.41) -> w-S (12.25) -> TSx-e-S (27.10) -> ao-e-S (26.06) -> TSao-e-S (26.94) -> Ru-de (13.26) -> Ru-OEt-e (7.44)
Highest Energy Peak on Path: 27.1 kcal/mol

Dearomatization pathways without explicit ethanol using TPSS-D4
Minimum-energy Path: Ru-H (0.00) -> TSq-R (24.15) -> r-R (24.06) -> TSap-R (28.82) -> Ru-de-R (13.26) -> aq-R (23.94) -> TSar-R (26.72) -> w-R (10.58) -> TSx-R (23.26) -> Ru-de (13.26) -> Ru-OEt-e (7.44)
Highest Energy Peak on Path: 28.82 kcal/mol

Dechelation pathways using M06
Minimum-energy Path: Ru-H (0.00) -> TSae (17.01) -> af (13.33) -> ag-R (12.90) -> TSah-R (24.40) -> ai-R (23.62) -> TSaj-e-S (26.92) -> k-R (19.25) -> TSam-R (21.04) -> m-R (17.20) -> TSn-R (23.43) -> o (15.42) -> TSp (20.17) -> Ru-OEt-e (8.78)
Highest Energy Peak on Path: 26.92 kcal/mol

Dechelation pathways without explicit ethanol using M06
Minimum-energy Path: Ru-H (0.00) -> TSae (17.01) -> af (13.33) -> ag-S (15.68) -> TSah-S (26.82) -> ai-S (26.95) -> TSaj-S (27.45) -> k-S (20.27) -> TSl-S (24.30) -> m-S (21.00) -> TSn-S (24.16) -> o (15.42) -> TSp (20.17) -> Ru-OEt-e (8.78)
Highest Energy Peak on Path: 27.45 kcal/mol

CH/OR metathesis pathways using M06
Minimum-energy Path: Ru-H (0.00) -> TSq-e-R (23.76) -> r-e-R (21.73) -> TSs-e-R (28.51) -> t-e-R (26.39) -> TSu-e-R (29.21) -> Ru-OEt-e (8.78)
Highest Energy Peak on Path: 29.21 kcal/mol

CH/OR metathesis pathways without explicit ethanol using M06
Minimum-energy Path: Ru-H (0.00) -> TSq-R (28.37) -> r-R (25.01) -> TSs-R (32.09) -> Ru-OEt-e (8.78)
Highest Energy Peak on Path: 32.09 kcal/mol

Dearomatization pathways using M06
Minimum-energy Path: Ru-H (0.00) -> TSq-e-R (23.76) -> r-e-R (21.73) -> TSv-e-R (27.31) -> w-R (11.99) -> TSx-R (29.38) -> Ru-de (8.55) -> Ru-OEt-e (8.78)
Highest Energy Peak on Path: 29.38 kcal/mol

Dearomatization pathways without explicit ethanol using M06
Minimum-energy Path: Ru-H (0.00) -> TSq-R (28.37) -> r-R (25.01) -> TSap-R (30.21) -> Ru-de-R (8.55) -> aq-R (21.50) -> TSar-R (28.53) -> w-R (11.99) -> TSx-R (29.38) -> Ru-de (8.55) -> Ru-OEt-e (8.78)
Highest Energy Peak on Path: 30.21 kcal/mol

Dechelation pathways using B3LYP-D3
Minimum-energy Path: Ru-H (0.00) -> TSae (16.07) -> af (12.90) -> ag-R (13.59) -> TSah-R (24.54) -> ai-R (23.77) -> TSaj-S (22.03) -> k-S (15.37) -> TSl-S (19.74) -> m-S (19.25) -> TSn-S (21.17) -> o (10.36) -> TSp (14.64) -> Ru-OEt-e (4.47)
Highest Energy Peak on Path: 24.54 kcal/mol

Dechelation pathways without explicit ethanol using B3LYP-D3
Minimum-energy Path: Ru-H (0.00) -> TSae (16.07) -> af (12.90) -> ag-R (13.59) -> TSah-R (24.54) -> ai-R (23.77) -> TSaj-S (22.03) -> k-S (15.37) -> TSl-S (19.74) -> m-S (19.25) -> TSn-S (21.17) -> o (10.36) -> TSp (14.64) -> Ru-OEt-e (4.47)
Highest Energy Peak on Path: 24.54 kcal/mol

CH/OR metathesis pathways using B3LYP-D3
Minimum-energy Path: Ru-H (0.00) -> TSq-e-S (22.68) -> r-e-S (19.87) -> TSs-e-S (21.64) -> t-e-S (20.90) -> TSu-e-S (19.97) -> Ru-OEt-e (4.47)
Highest Energy Peak on Path: 22.68 kcal/mol

CH/OR metathesis pathways without explicit ethanol using B3LYP-D3
Minimum-energy Path: Ru-H (0.00) -> TSq-R (25.01) -> r-R (21.55) -> TSs-R (25.10) -> Ru-OEt-e (4.47)
Highest Energy Peak on Path: 25.1 kcal/mol

Dearomatization pathways using B3LYP-D3
Minimum-energy Path: Ru-H (0.00) -> TSq-e-R (19.98) -> r-e-R (19.12) -> r-R (21.55) -> TSap-R (21.98) -> Ru-de-R (2.59) -> TSat-e-R (21.95) -> au-e-R (17.99) -> TSav-e-R (22.21) -> Ru-OEt-e (4.47)
Highest Energy Peak on Path: 22.21 kcal/mol

Dearomatization pathways without explicit ethanol using B3LYP-D3
Minimum-energy Path: Ru-H (0.00) -> TSq-R (25.01) -> r-R (21.55) -> TSap-R (21.98) -> Ru-de-R (2.59) -> as-R (17.28) -> TSat-R (22.74) -> Ru-OEt-e (4.47)
Highest Energy Peak on Path: 25.01 kcal/mol

**Aldehyde Hydrogenation**

Hydride attack/slippage pathways using ωB97M-V
Minimum-energy Path: Ru-H (5.41) -> TSz-e (17.57) -> aa-e (15.69) -> TSab-e (19.36) -> Ru-OEt-e (-1.52)
Highest Energy Peak on Path: 19.36 kcal/mol

Hydride attack/slippage pathways without explicit ethanol using ωB97M-V
Minimum-energy Path: Ru-H (5.41) -> TSz (21.34) -> aa (23.44) -> TSab (27.96) -> Ru-OEt-e (-1.52)
Highest Energy Peak on Path: 27.96 kcal/mol

Dechelation pathways using ωB97M-V
Minimum-energy Path: Ru-H (5.41) -> TSae (29.43) -> af (25.06) -> aw (13.90) -> TSax (16.98) -> o (11.27) -> TSp (16.38) -> Ru-OEt-e (-1.52)
Highest Energy Peak on Path: 29.43 kcal/mol

Dechelation pathways without explicit ethanol using ωB97M-V
Minimum-energy Path: Ru-H (5.41) -> TSae (29.43) -> af (25.06) -> aw (13.90) -> TSax (16.98) -> o (11.27) -> TSp (16.38) -> Ru-OEt-e (-1.52)
Highest Energy Peak on Path: 29.43 kcal/mol

Dearomatization pathways using ωB97M-V
Minimum-energy Path: Ru-H (5.41) -> TSz-e (17.57) -> aa-e (15.69) -> TSba-e (23.59) -> Ru-de (4.50) -> Ru-OEt-e (-1.52)
Highest Energy Peak on Path: 23.59 kcal/mol

Dearomatization pathways without explicit ethanol using ωB97M-V
Minimum-energy Path: Ru-H (5.41) -> TSz (21.34) -> aa (23.44) -> TSba (27.71) -> Ru-de (4.50) -> Ru-OEt-e (-1.52)
Highest Energy Peak on Path: 27.71 kcal/mol

Hydride attack/slippage pathways using M06L
Minimum-energy Path: Ru-H (4.90) -> TSz-e (17.17) -> aa-e (13.72) -> TSab-e (20.34) -> Ru-OEt-e (0.41)
Highest Energy Peak on Path: 20.34 kcal/mol

Hydride attack/slippage pathways without explicit ethanol using M06L
Minimum-energy Path: Ru-H (4.90) -> TSz (19.34) -> aa (18.76) -> TSab (26.13) -> Ru-OEt-e (0.41)
Highest Energy Peak on Path: 26.13 kcal/mol

Dechelation pathways using M06L
Minimum-energy Path: Ru-H (4.90) -> TSae (23.02) -> af (21.46) -> aw (12.82) -> TSax (17.30) -> o (7.53) -> TSp (10.44) -> Ru-OEt-e (0.41)
Highest Energy Peak on Path: 23.02 kcal/mol

Dechelation pathways without explicit ethanol using M06L
Minimum-energy Path: Ru-H (4.90) -> TSae (23.02) -> af (21.46) -> aw (12.82) -> TSax (17.30) -> o (7.53) -> TSp (10.44) -> Ru-OEt-e (0.41)
Highest Energy Peak on Path: 23.02 kcal/mol

Dearomatization pathways using M06L
Minimum-energy Path: Ru-H (4.90) -> TSz-e (17.17) -> aa-e (13.72) -> TSba-e (23.81) -> Ru-de (4.79) -> Ru-OEt-e (0.41)
Highest Energy Peak on Path: 23.81 kcal/mol

Dearomatization pathways without explicit ethanol using M06L
Minimum-energy Path: Ru-H (4.90) -> TSz (19.34) -> aa (18.76) -> TSba (26.09) -> Ru-de (4.79) -> Ru-OEt-e (0.41)
Highest Energy Peak on Path: 26.09 kcal/mol

Hydride attack/slippage pathways using MN15L
Minimum-energy Path: Ru-H (10.52) -> TSz-e (19.80) -> aa-e (19.95) -> TSab-e (27.02) -> Ru-OEt-e (6.93)
Highest Energy Peak on Path: 27.02 kcal/mol

Hydride attack/slippage pathways without explicit ethanol using MN15L
Minimum-energy Path: Ru-H (10.52) -> TSz (24.62) -> aa (25.71) -> TSab (34.87) -> Ru-OEt-e (6.93)
Highest Energy Peak on Path: 34.87 kcal/mol

Dechelation pathways using MN15L
Minimum-energy Path: Ru-H (10.52) -> TSae (32.40) -> af (33.24) -> aw (19.27) -> TSax (24.00) -> o (19.19) -> TSp (19.77) -> Ru-OEt-e (6.93)
Highest Energy Peak on Path: 33.24 kcal/mol

Dechelation pathways without explicit ethanol using MN15L
Minimum-energy Path: Ru-H (10.52) -> TSae (32.40) -> af (33.24) -> aw (19.27) -> TSax (24.00) -> o (19.19) -> TSp (19.77) -> Ru-OEt-e (6.93)
Highest Energy Peak on Path: 33.24 kcal/mol

Dearomatization pathways using MN15L
Minimum-energy Path: Ru-H (10.52) -> TSz-e (19.80) -> aa-e (19.95) -> TSba-e (31.01) -> Ru-de (13.91) -> Ru-OEt-e (6.93)
Highest Energy Peak on Path: 31.01 kcal/mol

Dearomatization pathways without explicit ethanol using MN15L
Minimum-energy Path: Ru-H (10.52) -> TSz (24.62) -> aa (25.71) -> TSba (34.68) -> Ru-de (13.91) -> Ru-OEt-e (6.93)
Highest Energy Peak on Path: 34.68 kcal/mol

Hydride attack/slippage pathways using TPSS
Minimum-energy Path: Ru-H (6.70) -> TSz-e (28.91) -> aa-e (27.26) -> TSab-e (34.32) -> Ru-OEt-e (16.57)
Highest Energy Peak on Path: 34.32 kcal/mol

Hydride attack/slippage pathways without explicit ethanol using TPSS
Minimum-energy Path: Ru-H (6.70) -> TSz (26.38) -> aa (27.59) -> TSab (35.95) -> Ru-OEt-e (16.57)
Highest Energy Peak on Path: 35.95 kcal/mol

Dechelation pathways using TPSS
Minimum-energy Path: Ru-H (6.70) -> TSae (28.37) -> af (22.35) -> aw (16.65) -> TSax (21.39) -> o (13.06) -> TSp (21.72) -> Ru-OEt-e (16.57)
Highest Energy Peak on Path: 28.37 kcal/mol

Dechelation pathways without explicit ethanol using TPSS
Minimum-energy Path: Ru-H (6.70) -> TSae (28.37) -> af (22.35) -> aw (16.65) -> TSax (21.39) -> o (13.06) -> TSp (21.72) -> Ru-OEt-e (16.57)
Highest Energy Peak on Path: 28.37 kcal/mol

Dearomatization pathways using TPSS
Minimum-energy Path: Ru-H (6.70) -> TSz (26.38) -> aa (27.59) -> TSba (34.16) -> Ru-de (6.05) -> Ru-OEt-e (16.57)
Highest Energy Peak on Path: 34.16 kcal/mol

Dearomatization pathways without explicit ethanol using TPSS
Minimum-energy Path: Ru-H (6.70) -> TSz (26.38) -> aa (27.59) -> TSba (34.16) -> Ru-de (6.05) -> Ru-OEt-e (16.57)
Highest Energy Peak on Path: 34.16 kcal/mol

Hydride attack/slippage pathways using TPSS-D4
Minimum-energy Path: Ru-H (8.67) -> TSz-e (18.58) -> aa-e (15.44) -> TSab-e (24.08) -> Ru-OEt-e (3.26)
Highest Energy Peak on Path: 24.08 kcal/mol

Hydride attack/slippage pathways without explicit ethanol using TPSS-D4
Minimum-energy Path: Ru-H (8.67) -> TSz (19.55) -> aa (20.23) -> TSab (30.62) -> Ru-OEt-e (3.26)
Highest Energy Peak on Path: 30.62 kcal/mol

Dechelation pathways using TPSS-D4
Minimum-energy Path: Ru-H (8.67) -> TSae (32.90) -> af (30.65) -> aw (18.85) -> TSax (23.63) -> o (14.29) -> TSp (18.52) -> Ru-OEt-e (3.26)
Highest Energy Peak on Path: 32.9 kcal/mol

Dechelation pathways without explicit ethanol using TPSS-D4
Minimum-energy Path: Ru-H (8.67) -> TSae (32.90) -> af (30.65) -> aw (18.85) -> TSax (23.63) -> o (14.29) -> TSp (18.52) -> Ru-OEt-e (3.26)
Highest Energy Peak on Path: 32.9 kcal/mol

Dearomatization pathways using TPSS-D4
Minimum-energy Path: Ru-H (8.67) -> TSz-e (18.58) -> aa-e (15.44) -> TSba-e (25.31) -> Ru-de (9.07) -> Ru-OEt-e (3.26)
Highest Energy Peak on Path: 25.31 kcal/mol

Dearomatization pathways without explicit ethanol using TPSS-D4
Minimum-energy Path: Ru-H (8.67) -> TSz (19.55) -> aa (20.23) -> TSba (26.81) -> Ru-de (9.07) -> Ru-OEt-e (3.26)
Highest Energy Peak on Path: 26.81 kcal/mol

Hydride attack/slippage pathways using M06
Minimum-energy Path: Ru-H (5.76) -> TSz-e (19.13) -> aa-e (15.30) -> TSab-e (20.32) -> Ru-OEt-e (1.55)
Highest Energy Peak on Path: 20.32 kcal/mol

Hydride attack/slippage pathways without explicit ethanol using M06
Minimum-energy Path: Ru-H (5.76) -> TSz (20.81) -> aa (20.78) -> TSab (27.43) -> Ru-OEt-e (1.55)
Highest Energy Peak on Path: 27.43 kcal/mol

Dechelation pathways using M06
Minimum-energy Path: Ru-H (5.76) -> TSae (22.77) -> af (19.09) -> aw (14.08) -> TSax (17.78) -> o (8.18) -> TSp (12.94) -> Ru-OEt-e (1.55)
Highest Energy Peak on Path: 22.77 kcal/mol

Dechelation pathways without explicit ethanol using M06
Minimum-energy Path: Ru-H (5.76) -> TSae (22.77) -> af (19.09) -> aw (14.08) -> TSax (17.78) -> o (8.18) -> TSp (12.94) -> Ru-OEt-e (1.55)
Highest Energy Peak on Path: 22.77 kcal/mol

Dearomatization pathways using M06
Minimum-energy Path: Ru-H (5.76) -> TSz-e (19.13) -> aa-e (15.30) -> TSba-e (24.12) -> Ru-de (1.31) -> Ru-OEt-e (1.55)
Highest Energy Peak on Path: 24.12 kcal/mol

Dearomatization pathways without explicit ethanol using M06
Minimum-energy Path: Ru-H (5.76) -> TSz (20.81) -> aa (20.78) -> TSba (26.25) -> Ru-de (1.31) -> Ru-OEt-e (1.55)
Highest Energy Peak on Path: 26.25 kcal/mol

Hydride attack/slippage pathways using B3LYP-D3
Minimum-energy Path: Ru-H (4.79) -> TSz-e (14.94) -> aa-e (10.88) -> TSab-e (11.34) -> Ru-OEt-e (-1.91)
Highest Energy Peak on Path: 14.94 kcal/mol

Hydride attack/slippage pathways without explicit ethanol using B3LYP-D3
Minimum-energy Path: Ru-H (4.79) -> TSz (16.82) -> aa (17.38) -> TSab (19.38) -> Ru-OEt-e (-1.91)
Highest Energy Peak on Path: 19.38 kcal/mol

Dechelation pathways using B3LYP-D3
Minimum-energy Path: Ru-H (4.79) -> TSae (20.86) -> af (17.69) -> aw (13.63) -> TSax (17.51) -> o (3.98) -> TSp (8.26) -> Ru-OEt-e (-1.91)
Highest Energy Peak on Path: 20.86 kcal/mol

Dechelation pathways without explicit ethanol using B3LYP-D3
Minimum-energy Path: Ru-H (4.79) -> TSae (20.86) -> af (17.69) -> aw (13.63) -> TSax (17.51) -> o (3.98) -> TSp (8.26) -> Ru-OEt-e (-1.91)
Highest Energy Peak on Path: 20.86 kcal/mol

Dearomatization pathways using B3LYP-D3
Minimum-energy Path: Ru-H (4.79) -> TSz-e (14.94) -> aa-e (10.88) -> TSba-e (13.99) -> Ru-de (-3.79) -> Ru-OEt-e (-1.91)
Highest Energy Peak on Path: 14.94 kcal/mol

Dearomatization pathways without explicit ethanol using B3LYP-D3
Minimum-energy Path: Ru-H (4.79) -> TSz (16.82) -> aa (17.38) -> TSba (18.19) -> Ru-de (-3.79) -> Ru-OEt-e (-1.91)
Highest Energy Peak on Path: 18.19 kcal/mol

**References**

(1) He, T.; Buttner, J. C.; Reynolds, E. F.; Pham, J.; Malek, J. C.; Keith, J. M.; Chianese, A. R. Dehydroalkylative Activation of CNN- and PNN-Pincer Ruthenium Catalysts for Ester Hydrogenation. *J. Am. Chem. Soc.* **2019,** *141*, 17404-17413.
